# One-pot organocatalysis of diacrylates to functional polyesters for toughening polylactide

**DOI:** 10.1093/nsr/nwaf437

**Published:** 2025-10-13

**Authors:** Yifan Zhao, Wentao Meng, Eugene Y-X Chen, Xiaoyan Tang

**Affiliations:** Beijing National Laboratory for Molecular Sciences, Key Laboratory of Polymer Chemistry and Physics of Ministry of Education, Center for Soft Matter Science and Engineering, College of Chemistry and Molecular Engineering, Peking University, Beijing 100871, China; Beijing National Laboratory for Molecular Sciences, Key Laboratory of Polymer Chemistry and Physics of Ministry of Education, Center for Soft Matter Science and Engineering, College of Chemistry and Molecular Engineering, Peking University, Beijing 100871, China; Department of Chemistry, Colorado State University, Fort Collins, CO 80523-1872, USA; Beijing National Laboratory for Molecular Sciences, Key Laboratory of Polymer Chemistry and Physics of Ministry of Education, Center for Soft Matter Science and Engineering, College of Chemistry and Molecular Engineering, Peking University, Beijing 100871, China

**Keywords:** diacrylates, hydrogen-transfer polymerization, unsaturated polyesters, one-pot reaction, PLLA toughening

## Abstract

Conventional chain-growth polymerization of acrylic monomers or diacrylics leads to non-degradable vinyl polymers or crosslinked networks, while existing step-growth polymerization affords saturated or main-chain unsaturated polyesters, hindering post-functionalization. Here, we introduce a hydrogen-transfer polymerization (HTP) strategy via the trialkylphosphine-catalyzed head-to-tail C–C coupling between the *α*- and *β*-positions of two vinyl groups of diacrylates with exclusive regioselectivity, producing unsaturated polyesters with side-chain double bonds that can undergo the subsequent thiol-Michael click reaction in a one-pot fashion with the carryover phosphine from the HTP step. The resulting hydroxyl-functionalized polyesters serve as macroinitiators for the efficient synthesis of densely grafted poly(l-lactide) (PLLA) bottlebrush polymers. Such bottlebrush polymers markedly toughen the otherwise brittle PLLA (by ∼10×), while not only uniquely preserving high melting temperature and crystallinity of PLLA but also synergistically increasing the PLLA crystallization rate.

## Teaser

This study introduces a hydrogen-transfer polymerization strategy transforming diacrylates into unsaturated polyesters, which are functionalized into hydroxyl-containing macroinitiators for bottlebrush polymers that enhance toughness and crystallization of biodegradable poly(L-lactide).

## INTRODUCTION

**Figure 1.**
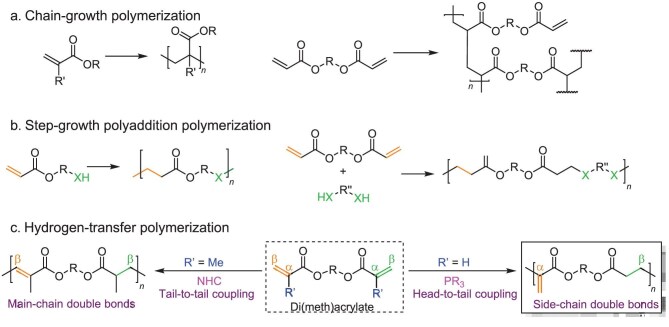
Polymerization strategies for (di)acrylates and (di)methacrylates. (a) Chain-growth polymerization of (meth)acrylates and di(meth)acrylates to linear poly(meth)acrylates and crosslinked thermosets, respectively. (b) Step-growth polyaddition polymerization of (di)acrylate monomers. (c) Step-growth HTP of diacrylates to unsaturated polyesters with double bonds being placed along the main-chain backbone or on the side chains. Z = H or CH_3_, R′, R″ = alkyl or aryl; X = O, S.

Acrylates, as low-cost, readily available, and industrially important monomers, have long been playing an important role in various areas of the polymer industry, including rubber, coatings, biomedical materials, adhesives, and textiles [1,2]. Owing to their highly reactive electron-deficient C═C double bonds, acrylates are amenable to a wide range of polymerization methods. Free radical polymerization is commonly employed for (di)acrylate polymerization (Fig. 1a). For example, copolymerization of acrylates with diacrylates (DAs) can produce hydrogels through the formation of crosslinking networks [3–5]. Additionally, living and controlled polymerization protocols [6], including anionic polymerization [7], zwitterionic polymerization [8], and group transfer polymerization [9], as well as the reversible-deactivation radical polymerization strategies, such as reversible addition-fragmentation chain transfer polymerization [10] and atom transfer radical polymerization [11], have been extensively developed for the polymerization of acrylic monomers to produce well-defined polyacrylates with tailored chemical sequences, molar masses, and dispersities. In the context of end-of-life options, these traditional polymerization strategies create all-carbon main chains through the addition reaction of C═C double bonds to form inert main-chain C–C bonds via the vinyl-addition polymerization (VAP) pathway, inhibiting degradability of the resulting vinyl polymers.

As *α,β*-unsaturated esters, acrylates can also serve as substrates for Michael-addition reactions, which enables step-growth polyaddition (co)polymerization of A-A or A-B type (di)acrylic monomers. In this context, oxa- [12,13] and thia-Michael [14,15] addition reactions have been reported to prepare polyesters with ether [16–19], thioester [20], or thiourethane [21] groups in their main chains or networks (Fig. 1b). These known step-growth polymerization methods typically result in the formation of saturated polyesters, lacking post-functionalization or modification options.

**Figure 2.**
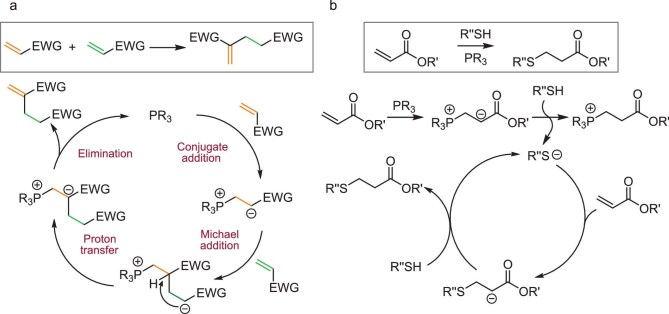
Proposed reaction mechanisms. (a) A proposed mechanism of Rauhut–Currier reaction. (b) A proposed mechanism of alkylphosphine-mediated thiol-Michael addition click reaction. EWG = electron withdrawing group; R = alkyl or aryl; R′, R″ = alkyl.

To not only gain polymer degradability by placing the ester bonds within the polymer main chains but also endow the polymers with desired post-functionalizability by installing double bonds on the side chains, we conceived a new hydrogen-transfer polymerization (HTP) pathway based on the Rauhut–Currier (R-C) reaction (Fig. 2a) to convert common DA monomers into unsaturated polyesters with pendent double bonds (Fig. 1b). The R-C reaction was first disclosed in 1963, in the form of the phosphine-catalyzed dimerization of activated alkenes, including acrylonitrile and ethyl acrylate [22]. Despite difficulties in controlling the selectivity of cross-coupling substrates that have limited the widespread application of the R-C reaction in organic synthesis, it remains an effective method for constructing carbon–carbon bonds between the *α*- and *β*-positions of two alkenes. A typical R-C reaction is proposed to proceed via the mechanism shown in Fig. 2a [23,24]. This process begins with the reversible conjugate addition of a nucleophile (e.g. trialkylphosphine) to the double bond, generating a zwitterionic species. This species then undergoes a Michael reaction with another equivalent of an electron-deficient alkene, forming a new zwitterionic intermediate. The intermediate subsequently undergoes a hydrogen-transfer process, followed by the elimination (regeneration) of the phosphine catalyst, ultimately yielding the head-to-tail coupling product. We hypothesized that such C–C coupling reaction could be utilized to polymerize DA monomers in a step-growth fashion, constructing unsaturated polyesters with main-chain ester bonds and side-chain double bonds, thus offering a variety of post-modification possibilities (Fig. 1c). Although the DABCO-catalyzed R-C polymerization of divalent vinyl ketones has been recently reported, it was noted that this approach is not applicable to diacrylate monomers [25].

It should be noted we previously reported *N*-heterocyclic carbene (NHC)-catalyzed HTP of dimethacrylates (Fig. 1c) [26,27], but it is likewise ineffective for polymerization of DAs. More importantly, the NHC-HTP system polymerizes dimethacrylates via tail-to-tail (or *β, β*) C–C bond coupling, leading to the corresponding unsaturated polyesters with double bonds being installed along the main-chain backbone. This double-bond placement hinders post-functionalization, particularly via a grafting-from approach to densely functionalized bottlebrush polymers, a major purpose of this work.

The thiol–ene click reaction is most desirable when considering the post-functionalization of unsaturated polymers. Given the advantages of rapid reaction rate, high selectivity, and mild conditions, the formation of C–S bonds proceeds via either free radical or Michael-addition pathways [28]. Nucleophilic alkylphosphines are simple yet effective for initiating the thiol-Michael addition click reaction between thiol and electron-deficient double bonds via an anionic mechanism (Fig. 2b). This mechanism inspired us to leverage the pre-existing (or carryover) trialkylphosphine employed in catalyzing the R-C reaction-based HTP step to mediate the post-functionalization of the acquired unsaturated polyester in a one-pot strategy. This type of one-pot organocatalytic approach [29] not only eliminates the need for additional labor-intensive and waste-generating quenching and purification steps but also provides a more coherent and design-driven strategy for utilizing the phosphine in both catalyzing polymerization and initiating post-functionalization processes.

Herein, based on the R-C reaction, we present a novel trialkylphosphine-catalyzed HTP method that differs from traditional acrylic polymerization systems. This method converts a wide range of DAs, each with varying functional linker groups connecting the two acrylate moieties, into unsaturated polyesters in pendent side-chain double bonds, instead of forming typical polyacrylates with all-carbon main chains or saturated polyesters that lack post-functionalization potential. Moreover, through the facile thiol-Michael addition click reaction, the resulting unsaturated polyesters can be readily modified in a one-pot process by simply adding thiols into the reaction mixture of the post-HTP reaction. Notably, the hydroxyl-modified polyesters are macroinitiators for the efficient synthesis of densely grafted poly(l-lactide) (PLLA) bottlebrush polymers that not only significantly toughen the otherwise brittle PLLA but also synergistically increase the PLLA crystallization rate.

## RESULTS AND DISCUSSION

### Catalyst-dependent competing VAP and HTP pathways

**Figure 3.**
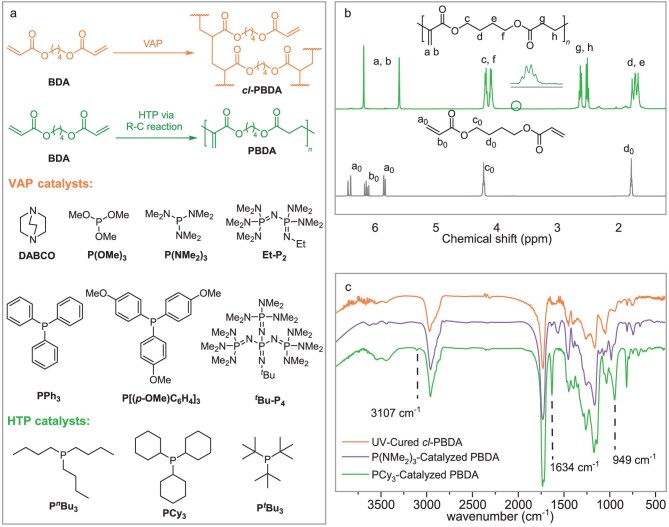
Polymerization of BDA as a model monomer and the characterization of the resulting polymer. (a) HTP and VAP pathways mediated by the nucleophilic catalysts employed in this work. (b) Overlaid ^1^H NMR (400 MHz, CDCl_3_) spectra of BDA monomer and PBDA polymer. (c) Overlaid FTIR spectra of PCy_3_-catalyzed PBDA, P(NMe_2_)_3_-catalyzed PBDA, and UV-cured *cl*-PBDA.

Earlier studies showed that catalysts such as PCy_3_ [30], P*^n^*Bu_3_ [31], P(NMe_2_)_3_ [32], PPh_3_ [31], and DABCO [33] were capable of catalyzing the dimerization of electron-deficient alkenes. These catalysts were therefore employed in our study to mediate the polymerization of 1,4-butanediol diacrylate (BDA), which was selected as the model monomer for optimizing the polymerization conditions. However, during this catalyst screening process, we found that, when P(OMe)_3_, PPh_3_, P[(*p*-OMe)C_6_H_4_]_3_, or DABCO was used as a nucleophile (Fig. 3a), no desired unsaturated polyester was obtained (Fig. S16A). Instead, the reaction after 2–6 h led to an insoluble solid within, indicative of a crosslinked product via the VAP pathway (Fig. 3a). Similar results were observed when a strong organophosphazene base, such as Et-P_2_ or *^t^*Bu-P_4_, was employed. Encouragingly, when P(NMe_2_)_3_ was used as the catalyst, a small amount of unsaturated polyester was detected by ^1^H NMR (Fig. S16B), consistent with the weak resonance of the pendent double bond protons at 5.61 and 6.19 ppm upon 5% BDA conversion in 2 h. However, the reaction mixture also turned into an insoluble solid after 6 h of reaction. Hence, while a minor fraction of BDA underwent HTP via the R-C reaction mechanism, the majority of BDA followed the VAP pathway, resulting in the formation of an insoluble crosslinked polymer (*vide infra*). Notably, when switching to trialkylphosphines, such as P*^n^*Bu_3_, PCy_3_, or P*^t^*Bu_3_ (with [BDA]: [P*^n^*Bu_3_] feeding molar ratio of 20:1; Entries 1, 9, and 14, Table 1), we successfully obtained soluble polymer PBDA with the desired pendent methylene groups through the HTP pathway, as evidenced by its ^1^H NMR spectrum with signals at 5.61 and 6.19 ppm (Fig. 3b, Figs S16C and S17). The structure of the resulting PBDA was further determined by ^13^C, ^1^H-^1^H COSY, and ^1^H-^13^C HSQC NMR spectra (Figs S18–S20). It is noteworthy that the ^13^C NMR signals at 172 and 166 ppm, corresponding to the ester carbonyl carbons, appear as singlets. This observation indicates a uniform ‘unsaturated–saturated’ head-to-tail connection of the repeating units; otherwise, the presence of ‘unsaturated–unsaturated’ or ‘saturated–saturated’ regioisomers would have led to signal splitting of the carbonyl resonances [25].

**Table 1.** Results of polymerization of BDA with different phosphines and solvents.^a^

Entry	PR_3_	Solvent	[BDA]:[PR_3_]	Time (h)	Conv. (%)^b^	*M*_n_ (kDa)^c^	*Ð* ^c^
1	P*^n^*Bu_3_	Toluene	20:1	7	94	3.9	1.87
2	P*^n^*Bu_3_	Toluene	50:1	36	63	2.1	1.51
3	P*^n^*Bu_3_	DCM	20:1	1	93	3.0	2.10
4	P*^n^*Bu_3_	DCE	20:1	1	88	2.9	1.85
5	P*^n^*Bu_3_	THF	20:1	33	91	2.7	1.73
6	P*^n^*Bu_3_	DOX	20:1	7.5	90	2.8	1.86
7	P*^n^*Bu_3_	MeCN	20:1		N.R.		
8	P*^n^*Bu_3_	*^t^* BuOH	20:1		N.R.		
9	PCy_3_	Toluene	20:1	6.5	96	5.8	1.90
10	PCy_3_	DCM	20:1	0.5	91	5.1	2.12
11	PCy_3_	DCE	20:1	3.5	87	4.9	1.64
12	PCy_3_	THF	20:1	3.5	92	5.1	1.80
13	PCy_3_	DOX	20:1	3	84	5.1	2.19
14	P*^t^*Bu_3_	Toluene	20:1	6	13		
15^d^	P*^t^*Bu_3_	Toluene	20:1	12	33		

a Conditions: [BDA] = 1.5 mol/L, 0.5 mmol scale, room temperature. ^b^Conversion of BDA was measured by ^1^H NMR spectroscopy. ^c^Number-average molar mass (*M*_n_) and dispersity (*Đ* = *M*_w_/*M*_n_) determined by size exclusion chromatography (SEC) at 35°C in THF relative to polystyrene standards. ^d^Temperature = 60°C. DCE = 1,2-dichloroethane, DCM = dichloromethane, DOX = 1,4-dioxane, THF = tetrahydrofuran, N.R. = no reaction.

To verify the structure of the crosslinked polymer obtained above, we synthesized a comparative crosslinked polymer via UV-curing of BDA using 2,2-dimethoxy-2-phenylacetophenone (DMPA) as a free radical photo-initiator. The Fourier transform infrared (FTIR) spectrum (Fig. 3c) of PBDA obtained from the PCy_3_-catalyzed polymerization (Entry 9, Table 1) showed absorbance at wavenumbers 3107, 1634, and 949 cm^−1^, which can be assigned to ═C(H)–H stretching, C═C stretching, and ═C(H)–H wagging vibration of the pendent double bonds formed via HTP, respectively. In contrast, the characteristic IR absorbance of the double bonds was absent in the UV-cured crosslinked PBDA (*cl*-PBDA) product, as they were completely consumed by the DMPA-initiated free radical crosslinking VAP process. Furthermore, the P(NMe_2_)_3_-catalyzed polymerization of BDA led to a mixture of VAP and HTP products.

With the three trialkylphosphine catalysts (PR_3_ = P*^n^*Bu_3_, PCy_3_, and P*^t^*Bu_3_) achieving the desired HTP of BDA in hand, we proceeded to optimize the polymerization conditions. When the [BDA]:[P*^n^*Bu_3_] ratio was 20:1, the conversion of BDA reached 94% in 7 h, producing PBDA with a low number-average molar mass (*M*_n_) of 3.9 kDa and a dispersity (*Ð*) of 1.87 (Fig. S40), whereas a lower catalyst loading ([BDA]:[P*^n^*Bu_3_] = 50:1) resulted in a much slower polymerization rate (63% conversion in 36 h) and a lower *M*_n_ of 2.1 kDa (Entries 1–2, Table 1). Based on these results, the [BDA]:[PR_3_] ratio was set to 20:1 for subsequent polymerization studies. It is important to note that the reaction mixture eventually turned into an insoluble gel as the conversion of BDA approached slightly below 100%, which is attributable to the formation of insoluble networks by the further reaction between phosphine with pendent double bonds of the resulting PBDA after nearly complete conversion of BDA (Fig. S22). Hence, to obtain soluble unsaturated polymers, we quenched the polymerizations before the reaction mixture turned into an insoluble gel, i.e. before reaching 100% monomer conversion. Therefore, the conversion data of BDA monomer in Table 1 reflects the conversions at the time the reactions were quenched, rather than the ultimate conversion that could be achieved at equilibrium.

Among these catalysts, PCy_3_ outperformed P*^n^*Bu_3_, exhibiting a higher catalytic rate with 96% conversion of BDA after 6.5 h and affording PBDA in an isolated yield of 92% with a higher *M*_n_ of 5.8 kDa (Entries 1 vs 9, Table 1; Figs S41 and S42). Notably, the HTP of BDA catalyzed by PCy_3_ can be readily scaled up to at least 20 g, thanks to the accessibility of both the BDA monomer and PCy_3_ catalyst. At this larger scale, the HTP still achieved high monomer conversion (93%, 7.5 h) and afforded PBDA with a molecular weight (*M*_n_ of 5.8 kDa; Fig. S43) comparable to that obtained from the small scale run, demonstrating the practical scalability of the process. In comparison, the BDA conversion in the P*^t^*Bu_3_-catalyzed reaction remained low, even at the elevated temperature of 60°C (Entries 14–15, Table 1), presumably due to the bulkiness of the *tert*-butyl groups, which hinder the nucleophilic attack of phosphorus atoms at the double bond. PCy_3_ and P*^n^*Bu_3_ were then tested in different solvents for optimization. The polymerization of BDA exhibited the highest activity in dichloromethane (DCM) for both catalysts (Entries 1, 3–6, and 9–13, Table 1), while no HTP product was formed in MeCN and *^t^*BuOH (Entries 7–8, Table 1). It is worth mentioning that the faster polymerizations in DCM led to premature gelation; hence, we selected toluene as the solvent to slow down the reaction, allowing for timely quenching at higher conversions but before gelation and consequently achieving higher molecular weights of the polymers.

### Monomer scope and thermal properties of resulting polyesters

With the aim of verifying the generalizability of the phosphine-catalyzed HTP method and obtaining polymers with varied properties, we extended the HTP of BDA to diacrylates bearing different linker groups, including 1,6-hexanediol diacrylate (HDA), 1,8-octanediol diacrylate (ODA), and 1,12-dodecanediol diacrylate (DoDA) with alkyl linkers; 2,2_ʹ_-[(dimethylsilylene)dioxy] diacrylate (SiDA) with a silyl ether linker; *p*-xylylene glycol diacrylate (XDA) and bisphenol A diacrylate (BPDA) with aromatic linkers; diethylene glycol diacrylate (GDA) with an ether linker; and 1,4-butanediol diacrylate urethane (BDDU) with urethane bonds, as compiled in Table 2. Most of these diacrylates can be readily prepared by reacting the corresponding diols with acryloyl chloride [34], while SiDA was prepared through the substitution reaction of SiMe_2_Cl_2_ with 2-hydroxyethyl acrylate [35], and BDDU was synthesized via the addition reaction of 1,4-butanediol and 2-isocyanatoethyl acrylate (Figs S1–S15) [36].

**Table 2.**
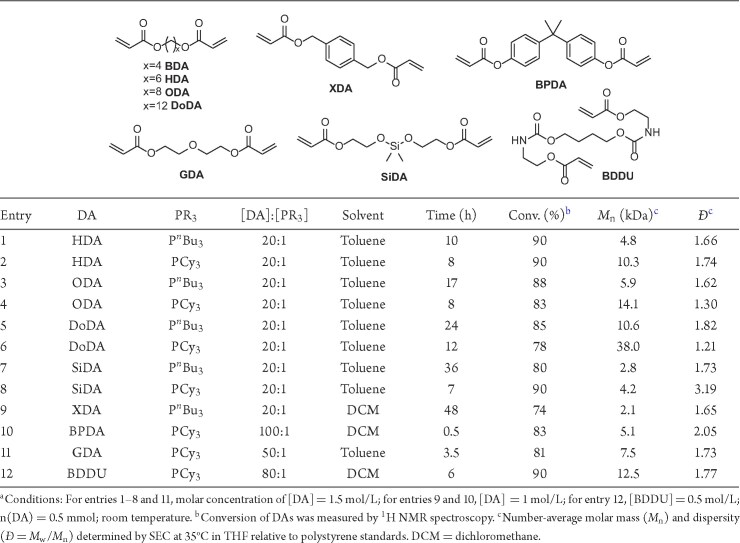
Results of HTP of DAs with different linker groups.^a^

As summarized in Table 2, all the DAs can undergo HTP catalyzed by P*^n^*Bu_3_ and PCy_3_, producing the corresponding unsaturated polyesters, all of which were characterized by ^1^H and ^13^C NMR spectra (Figs S23, S24 and S26–S39). Consistent with the trends observed in the catalyst screening for BDA polymerization, PCy_3_ demonstrated higher catalytic activity than P*^n^*Bu_3_, affording unsaturated polyesters with higher molar masses (Entries 1–8, Table 2; Figs S43–S45). For the alkyl-linked DAs (BDA, HDA, ODA, and DoDA), the polymerization rate decreased with increasing the length of the alkyl linker, although higher molecular weights were achieved with the longer alkyl linkers, with *M*_n_ values reaching up to 38.0 kDa for PDoDA (Entries 1 and 9, Table 1; Entries 1–6, Table 2). However, DAs with the silyl ether, aromatic linker, and ether linker can only be polymerized to yield low-molar-mass unsaturated polyesters with *M*_n_ = 2.1–7.5 kDa. It should be noted that DCM was used as solvent for XDA, BPDA, and BDDU polymerizations instead of toluene to improve solubility, yielding polymers with *M*_n_ values of up to 12.5 kDa (Entries 9, 10, and 12, Table 2; Fig. S48). Additionally, BPDA, GDA, and BDDU showed higher reactivity relative to the alkyl-linked analogs, thus requiring lower catalyst loadings ([DA]:[PR_3_] = 50:1 to 100:1) and shorter times (0.5–6 h) to achieve high conversions of monomers (Entries 10–12, Table 2). In general, the HTP method via the R-C reaction exhibits a broad diacrylate monomer scope, providing a general approach to synthesize unsaturated polyesters with diverse functionalities on the main chain and double bonds on the side chains.

Similar to the BDA polymerization, gelation also occurred for DAs with different linker groups, typically at high monomer conversions prior to complete consumption. Therefore, timely quenching was essential to avoid the formation of a crosslinking network. As in Table 1, the conversion data presented in Table 2 were collected at the quenching timepoint of the reactions, and the resulting polyesters were soluble in CHCl_3_ or THF for further NMR and SEC characterizations. To investigate the gelled products, alkyl-linked monomers such as BDA, HDA, ODA, and DoDA were selected as representative examples. Their reaction mixtures were allowed to gel (i.e. complete monomer conversion) in Teflon molds, and the residual toluene in the crosslinking network of the insoluble gel was removed under vacuum. The average molar mass between crosslinking points (*M*_c_) was calculated by the Flory–Rehner equation [37], based on their swelling ratio in DCM (Table S1). For the alkyl-linked gelled products, the average molar mass between crosslinking points was found to be 8∼10 monomer units, calculated by dividing the value obtained for *M*_c_ by the molar mass of the DAs (*M*_DA_).

**Figure 4.**
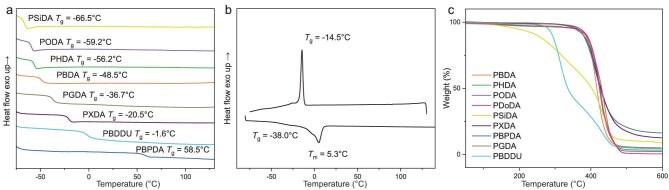
Thermal properties of unsaturated polyesters obtained by HTP of DAs. (a) DSC thermograms of the non-crystallizable unsaturated polyesters (Entry 9, Table 1; Entries 2, 4, 8–12, Table 2). (b) DSC thermograms of PDoDA (Entry 6, Table 2). (c) TGA thermograms of unsaturated polyesters.

The thermal properties of these unsaturated polyesters (Entry 9, Table 1; Entries 2, 4, 6, and 8–12, Table 2) were evaluated using differential scanning calorimetry (DSC) and thermal gravimetric analysis (TGA) methods. Second DSC heating scans revealed that the glass transition temperature (*T*_g_) of the polymers increased with the increasing rigidity of the main chains, following the trend: PSiDA (−66.5°C) < PODA (−59.2°C) < PHDA (−56.2°C) < PBDA (−48.5°C) < PDoDA (−38.0°C) < PGDA (−36.7°C) < PXDA (−20.5°C) < PBDDU (−1.6°C) < PBPDA (58.5°C) (Fig. 4a). No distinct crystallization temperature (*T*_c_) or melting temperature (*T*_m_) was observed for these polyesters under DSC conditions, except for PDoDA, which exhibited *T*_m_ = 5.3°C and *T*_c_ = −14.5°C (Fig. 4b). The lack of crystallinity in most of the unsaturated polyesters is likely attributed to the pendent double bonds, which could disrupt crystallization, whereas the crystallinity observed in PDoDA is likely due to the crystallization of the long (12 carbon atom) alkyl linker.

TGA and derivative thermogravimetry (DTG) curves of the polyesters are provided in Fig. 4c and Figs S49–S57. PSiDA showed the lowest thermal stability, with a degradation temperature (*T*_d_, defined at 5% weight loss) of 193°C, and a two-step degradation profile featuring maximum rate decomposition temperatures (*T*_max_) at 310°C and 430°C (Fig. S53). A similar two-step degradation profile was also observed for PBDDU (*T*_max1_ = 314°C, *T*_max2_ = 434°C), but with a higher *T*_d_ of 280°C (Fig. S57). Their two-step degradation is probably due to the differing thermal stabilities of silyl ether and urethane linkers, compared to ester groups. In contrast, all other polyesters, except for PSiDA and PBDDU, exhibited high thermal stabilities with one-step degradation profiles, showing *T*_d_ values ranging from 349 to 370°C and *T*_max_ values ranging from 415 to 437°C.

### Mechanistic considerations

**Figure 5.**
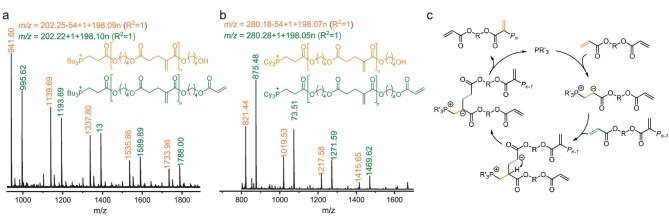
Mechanistic considerations. MALDI-TOF mass spectrum of (aa) P*^n^*Bu_3_-catalyzed PBDA (Entry 1, Table 1) and (b) PCy_3_-catalyzed PBDA (Entry 9, Table 1). (c) A proposed propagation cycle of the phosphine-catalyzed HTP. R = alkyl or aryl, R′ = alkyl, *P_n_* = polymer chain with *n* monomer units.

To further confirm the structure of the resulting unsaturated polyesters and investigate the polymerization mechanism, the chain end structure of PBDA was analyzed by matrix-assisted laser desorption ionization time-of-flight mass spectrometry (MALDI-TOF, Fig. 5a and b). Two distinct sets of molecular ion mass (*m/z*) signals were observed, with the spacing between neighboring peaks corresponding to the exact molar mass of BDA (*m/z* = 198) for both P*^n^*Bu_3_- and PCy_3_-catalyzed PBDA. Interestingly, one set showed the molar mass of the end group (*M*_end_) as 203 and 281 for P*^n^*Bu_3_- and PCy_3_-catalyzed PBDA, respectively, indicating the presence of a phosphonium cation group resulting from the PR_3_-vinyl addition. This end group was further confirmed by the ^31^P NMR spectrum (Fig. S21), and similar reactions can be found in previous studies [38]. The other set showed *M*_end_ values of 149 (i.e. 203–54) and 227 (i.e. 281–54), resulting from a deacryloylation process mediated by the phosphine, forming HO–CH_2_–CH_2_–CH_2_–CH_2_– as the other end group. This end group was confirmed by ^1^H NMR, with the methylene protons of the terminal –CH_2_–OH appearing at 3.68 ppm, and the adjacent methylene protons along the chain observed at 2.32 ppm and 1.89 ppm, respectively (Fig. 3b). The deacryloylation of *α*-vinylidene polyesters in the presence of a Lewis base has also been reported previously [39]. Furthermore, a similar pattern of molecular ion peaks in the MALDI-TOF spectra was observed for the PHDA synthesized by using P*^n^*Bu_3_ and PCy_3_, respectively (Entries 1–2, Table 2), showing the same phosphonium group and the deacryloylated chain end (Fig. S25). Therefore, the HTP of DAs is proposed to proceed via the propagation mechanism postulated in Fig. 5c, which is analogous to the phosphine-catalyzed dimerization of electron-deficient alkenes (*c.f.*, Fig. 2a).

### One-pot PR_3_-mediated HTP and post-functionalization

**Figure 6.**
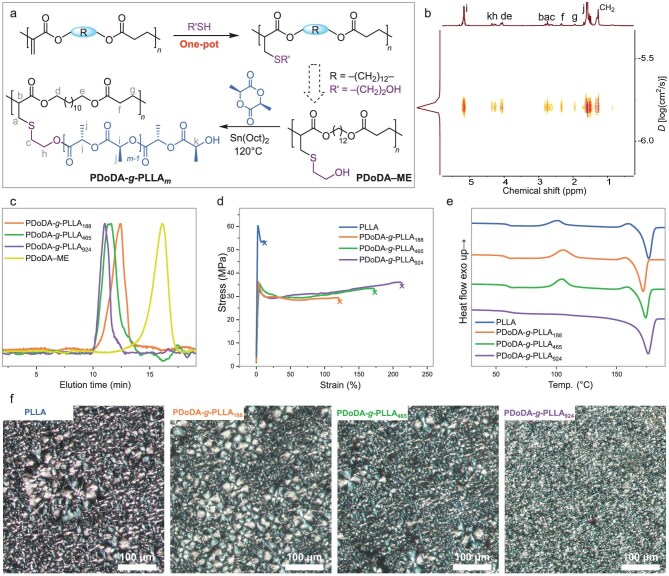
One-pot post-functionalization and toughening of PLLA by forming bottlebrush polymers. (a) Schematic route of the one-pot click reaction using the same carryover phosphine, followed by the ROP of l-LA with PDoDA–ME as a macroinitiator and Sn(Oct)_2_ as the catalyst to form bottlebrush polymers. (b) DOSY NMR spectrum of PDoDA-*g*-PLLA*_m_*. (c) SEC chromatograms of PDoDA-*g*-PLLA*_m_* and PDoDA–ME. (d) Tensile tests of the PDoDA-*g*-PLLA*_m_* and PLLA from a batch of at least 3 replicates. (e) The second heating DSC curves of PDoDA-*g*-PLLA*_m_* and PLLA. (f) POM images of PLLA, PDoDA-*g*-PLLA_188_, PdoDA-*g*-PLLA_465_, and PDoDA-*g*-PLLA_924_ (left to right).

The thiol–ene click reaction between thiol reagents and double bonds is widely considered to be one of the most efficient methods for the post-functionalization of unsaturated polymers. Previous studies have primarily focused on demonstrating the feasibility of this reaction to post-functionalize unsaturated polyesters that were typically isolated from the polymerization mixture and purified before the modification step [39–43]. In contrast, our system takes advantage of the pre-existing trialkylphosphine catalyst from the R-C reaction-based HTP, enabling a one-pot thiol–ene Michael addition click reaction for the post-modification of the *in-situ* generated unsaturated polyester (Fig. 6a).

Specifically, in a typical PCy_3_-catalyzed HTP reaction system (Entry 9, Table 2), when the conversion of BDA reached 96% after 6.5 h, an excess of 2-mercaptoethanol (ME, 10 equivalents to BDA monomer) in toluene was added to the reaction solution. After an additional 4 h, the signals for the double bonds at 6.19 and 5.61 ppm completely disappeared, and the target post-functionalized polymer PBDA–ME was obtained (Figs S58 and S59). To validate the outcome of this one-pot thiol-Michael addition click reaction, we also performed a conventional free radical thiol–ene click reaction to obtain PBDA–ME for comparison. In this case, the isolated PBDA was reacted with ME (10 equivalents to the amount of double bonds) in the presence of DMPA (5 wt%) as the photocatalyst in THF. The resulting polymer exhibited the same structure as the PBDA–ME polymer obtained via the one-pot reaction initiated by PCy_3_, as confirmed by the overlaid ^1^H NMR spectra (Fig. S60), further demonstrating the success of the PCy_3_-mediated one-pot strategy. To confirm that the one-pot post-polymerization with ME is indeed initiated by the phosphine, the BDA polymerization by PCy_3_ was quenched with HCl/methanol (0.1 M) to remove any residual phosphine from the resulting PBDA (Fig. S61); under these conditions, no reaction of the HCl/methanol-quenched PBDA with ME was observed over 6 h (Fig. S62), demonstrating the central role of phosphine in the post-polymerization one-pot click reaction. Additionally, benzyl mercaptan (BnSH) was also employed in this one-pot modification method, and the resulting product was consistent with the photocatalyzed radical click reaction product, PBDA–BnSH (Figs S63–S65). The same one-pot click reaction was also applied to PDoDA (Entry 6, Table 2), producing the corresponding modified polyester PDoDA–ME (Fig. 6a and Figs S66, S67). These results suggest that the post-modification of the *in-situ* generated unsaturated polyester was successfully achieved in a one-pot fashion using the carryover trialkylphosphine employed for the prior HTP of DAs (Fig. 6a).

### Toughening of poly(l-lactide) by bottlebrush polymers

The unique advantage of unsaturated polyesters with pendent double bonds lies in their potential for facile post-modification reactions, generating new materials with tailored properties. In particular, PBDA–ME and PDoDA–ME containing hydroxyl groups on their side chains can serve as macroinitiators to synthesize bottlebrush polymers, the materials that have gained significant attention for their potential applications in diverse fields such as colloid science, biomedicine, and surface modification [44]. In this context, we explored the potential of PDoDA–ME, derived from the highest-molecular-weight polyester PDoDA (*M*_n_ = 38.0 kDa, *Ð* = 1.21), to toughen technologically important yet brittle PLLA, as its relatively long backbone is expected to better promote the formation of robust bottlebrush architectures. Specifically, we employed the ring-opening polymerization (ROP) of l-lactide (l-LA) using PDoDA–ME as the macroinitiator and stannous octoate (Sn(Oct)_2_) as the catalyst to produce bottlebrush polymer PDoDA-*g*-PLLA*_m_* through a ‘grafting-from’ approach (Fig. 6a).

By varying the molar ratio of l-LA to –OH group of PDoDA–ME, [l-LA]:[–OH], from 200:1 to 1000:1, a series of bottlebrush polymers PDoDA-*g*-PLLA*_m_* with varying densely grafted PLLA side chain lengths (where the subscript ‘*m*’ denotes the number of l-LA units in the side chain), namely PDoDA-*g*-PLLA_188_, PDoDA-*g*-PLLA_465_, and PDoDA-*g*-PLLA_924_, were prepared (Table 3). Their structures were first analyzed by ^1^H NMR spectra, where the methylene protons adjacent to the side hydroxyl groups of PDoDA–ME at 3.72 ppm completely disappeared after the grafting ROP (Fig. S69), indicating that the grafting density is essentially 100%. Furthermore, the singular diffusion coefficient peak in the DOSY NMR spectrum (Fig. 6b) confirmed that the polymer consisted solely of PDoDA-*g*-PLLA*_m_* brushes without homopolymer contaminants. The SEC chromatograms also shifted to higher molar masses compared to the starting PDoDA–ME (Fig. 6c).

**Table 3.** Thermal and mechanical properties of bottlebrush polymer PDoDA-*g*-PLLA*_m_* and homopolymer PLLA.

*m* ^a^	[l-LA]:[–OH]	Conv. (%)^a^	*M*_n_ (kDa)^b^	*Ð* ^b^	*E* (GPa)	*ε*_b_ (%)	*U*_T_^c^ (MPa)	*T*_m_ (°C)	Δ*H*_f_ (J/g)
188	200:1	94.4	638.5	1.33	1.28 ± 0.15	131 ± 10	37.7 ± 2.9	171.9	34.43
465	500:1	93.1	1244	1.62	1.25 ± 0.09	171 ± 7	53.7 ± 3.0	174.2	31.30
924	1000:1	92.4	1902	1.32	1.22 ± 0.07	198 ± 11	61.1 ± 5.0	176.1	30.24
	PLLA		788.1	1.41	2.97 ± 0.08	12.1 ± 0.5	6.4 ± 0.2	176.9	36.76

a The length of the side PLLA chains (*m*) calculated by the l-LA/–OH feeding ratio and the conversion of l-LA as determined by ^1^H NMR. ^b^Number-average molar mass (*M*_n_) and dispersity (*Đ* = *M*_w_/*M*_n_) determined by SEC at 35°C in CHCl_3_ relative to polystyrene standards. ^c^The toughness was calculated by the integration of the area under the stress–strain curves.

The bottlebrush polymers PDoDA-*g*-PLLA*_m_* were solution-cast into films and cut into dogbone-shaped specimens for the subsequent tensile testing to characterize their mechanical properties. Notably, these bottlebrush polymers exhibited significantly higher toughness (*U*_T_ ranging from 37.7 to 61.1 MPa) compared to homopolymer PLLA (*U*_T_ = 6.4 MPa), Fig. 6d. As the length of the side PLLA chains in the bottlebrush polymers increased, their Young’s modulus (*E*) remained high at ∼1.2 GPa and almost unchanged, while the elongation at break (*ε*_b_) increased from 131% to 198%, which is significantly higher than that of PLLA (*ε*_b_ = 12%). Importantly, the *T*_m_ of the PDoDA-*g*-PLLA*_m_* slightly increased with the length of the side chains, yet no significant difference in enthalpy of fusion (Δ*H*_f_) was observed between homo-PLLA and PDoDA-*g*-PLLA*_m_*, as evidenced by the DSC thermograms (Fig. 6e). Notably, compared with other two bottlebrush polymers carrying shorter side chains or even with PLLA, PDoDA-*g*-PLLA_924_ exhibited a faster crystallization behavior, as no cold crystallization peak was observed at the same cooling and heating rate of 10°C/min (Fig. 6e and Figs S75–S78). Polarized optical microscopy (POM) further revealed the origin of both the accelerated crystallization and the enhanced mechanical toughness. As shown in Fig. 6f, the crystallite size follows the order: PLLA ≈ PDoDA-*g*-PLLA_188_ > PDoDA-*g*-PLLA_465_ > PDoDA-*g*-PLLA_924_. The formation of smaller crystallites in PDoDA-*g*-PLLA_924_ increases the density of nucleation sites, thereby lowering the barrier to crystallization and accelerating crystal growth. At the same time, the finer crystallite morphology reduces local stress concentrations and facilitates more uniform plastic deformation, collectively contributing to the enhanced toughness of this material.

To further investigate the effect of the macroinitiator’s main-chain length, PDoDA polymers with different molecular weights (*M*_n_ = 5.3 kDa, *Ð* *=* 1.99, denoted as PDoDA1; *M*_n_ = 7.8 kDa, *Ð* *=* 1.94, denoted as PDoDA2; see Supplementary Data for details), in addition to the above described PDoDA (*M*_n_ = 38.0 kDa, *Ð* = 1.21), were synthesized and used for PLLA grafting. The results (Table S2 and Fig. S74) show that bottlebrush polymers with shorter main chains (PDoDA1-*g*-PLLA_953_ and PDoDA2-*g*-PLLA_962_) also exhibit improved toughness compared to PLLA homopolymer, although their toughening effect is less pronounced than that of PDoDA-*g*-PLLA_924_. Nevertheless, they still showed cold crystallization peaks (Fig. S79), indicating slower crystallization than the bottlebrush polymers with longer main chains. These findings underscore the critical role of the main-chain length in tuning the thermal properties, particularly crystallization, of the resulting bottlebrush polymers.

While numerous strategies have been reported to overcome the inherent brittleness of PLA [45–48], including blending, copolymerization, mixing PLLA homopolymer with plasticizers, or grafting poly(d, l-lactide) side chains from macroinitiators to prepare bottlebrush polymers [49,50], most of these methods improved the ductility of PLA at the expense of reduced *T*_m_ and Δ*H*_f_. The ‘grafting from’ strategy we present here not only effectively enhances the toughness of PLLA without compromising *T*_m_ and Δ*H*_f_ but also increases the crystallization rate of PLLA (which is another inherent limitation of PLLA), offering a distinct advantage over other copolymerization approaches.

## CONCLUSION

This work developed a novel HTP of diacrylates for synthesizing unsaturated polyesters carrying a pendent double bond in each repeating unit through the Rauhut–Currier reaction. Trialkylphosphines such as P*^n^*Bu_3_ and PCy_3_ effectively promoted the HTP process, with PCy_3_ exhibiting higher catalytic activity, producing unsaturated polyesters with *M*_n_ up to 38.0 kDa. This HTP method was successfully applied to five types of diacrylates bearing alkyl, silylether, aryl, ether, and urethane linkers, affording the corresponding unsaturated polyesters efficiently.

Utilizing the carryover phosphine employed for the HTP step, the one-pot thiol-Michael addition click reaction of the *in-situ* generated unsaturated polyesters enabled the direct and facile synthesis of hydroxyl-functionalized polyesters PDoDA–ME and PBDA–ME. Notably, PDoDA–ME, featuring hydroxyl side chains, served as efficient macroinitiators for the ‘grafting-from’ ROP of l-LA in the presence of Sn(Oct)_2_ as the catalyst, affording high-molar-mass bottlebrush copolymers with varying grafted PLLA side chain lengths and *M*_n_ up to 1.9 million Da. Unlike other reported PLA toughening strategies, the strategy described herein effectively toughened PLLA without compromising its *T*_m_ and crystallinity, while synergistically increasing its crystallization rate.

Overall, this phosphine-catalyzed HTP system exhibits three unique features. First, it produces degradable unsaturated polyesters from industrially important acrylic monomers, which differ from prior polymers obtained by other systems including: main-chain C–C bonded vinyl polymers by conventional chain-growth vinyl-addition polymerization of acrylics; saturated polyesters from step-growth polymerization of hydroxyl-containing acrylics; and unsaturated polyesters with main-chain double bonds from NHC-catalyzed HTP of dimethacrylates. Second, the phosphine catalyst employed in the HTP step also initiates the subsequent thiol-ene click post-functionalization, thus enabling facile polymer synthesis and post-functionalization in a one-pot process without the addition of an extra catalyst or initiator. Third, the pendent, side-chain double bonds allow for efficient synthesis of densely grafted bottlebrush polymers that effectively toughen PLLA and synergistically increase the PLLA crystallization rate without compromising its high *T*_m_ and crystallinity.

## METHODS

### Materials, reagents, and characterization methods

The source of materials and reagents, and characterization methods used can be found in the supplementary information.

### Polymerization of DAs

Taking the polymerization of BDA as a representative example (Entry 9, Table 1). In an argon-filled glovebox, BDA (99 mg, 0.5 mmol) was dissolved in toluene (190 μL) in a 5 mL vial containing a stir bar, then PCy_3_ (50 μL, 0.5 mol/L in toluene) was added to the above solution to set the initial concentration of monomer to 1.5 mol/L and [monomer]:[catalyst] ratio to 20:1. The vial was sealed and left to stir in the glovebox under room temperature, or taken out of the glovebox to be placed in a heating block if a higher reaction temperature was needed. At desired time points, a small aliquot was analyzed by ^1^H NMR characterization to determine the conversion. The polymerization was quenched by adding CHCl_3_, and then the diluted reaction mixture was precipitated into an excess of *n*-pentane and centrifugated. The precipitate was then dissolved in CHCl_3_ (2 mL) again, precipitated into an excess of *n*-pentane and centrifugated for three more cycles. The precipitate was finally dried under vacuum.

### Preparation of bottlebrush copolymers PDoDA-*g*-PLLA*_m_*

Taking the synthesis of PDoDA-*g*-PLLA_188_ as a representative example. To a flame-dried Schlenk tube equipped with a magnetic stir bar, l-LA (720 mg, 5 mmol), a stock solution of PDoDA–ME (250 μL, THF as solvent, [–OH] = 0.1 mol/L), a stock solution of stannous octoate (10 μL, toluene as solvent, [Sn(Oct)_2_] = 0.5 mol/L), and 2 mL toluene was added. Then the tube was sealed with a Teflon lined cap, taken out from the glovebox and placed in a 120°C heating block. The reaction mixture was left to stir for 12 h. Then the tube was taken out of the heating block and cooled to room temperature to quench the polymerization. The residue was dissolved in DCM (10 mL) and then precipitated into an excess of cold methanol. The obtained precipitate was washed with methanol several times and then dried under vacuum at room temperature.

## Supplementary Material

nwaf437_Supplemental_File
