# Supplementary material for: One-pot organocatalysis of diacrylates to functional polyesters for toughening polylactide
Source: Natl Sci Rev. 2025 Oct 13;12(12):nwaf437. doi: 10.1093/nsr/nwaf437 (PMC12693503; doi:10.1093/nsr/nwaf437)
Supplement: nwaf437_Supplemental_File [file nwaf437_supplemental_file.pdf]

One-Pot Organocatalysis of Diacrylates to Functional Polyesters for Toughening Polylactide

Yifan Zhao, Wentao Meng, Eugene Y.-X. Chen, and Xiaoyan Tang

Table of Contents

Methods **5**

Nuclear Magnetic Resonance (NMR) 5

Size Exclusion Chromatography (SEC) 5

Matrix-Assisted Laser Desorption Ionization Time-of-Flight Mass Spectroscopy (MALDI-TOF MS) 5

Fourier Transform Infrared Spectroscopy (FTIR) 6

Thermogravimetric Analysis (TGA) 6

Differential Scanning Calorimetry (DSC) 6

Tensile Tests 6

Synthesis of Monomers **7**

Figure S1. Synthesis of diacrylate monomers. 7

Preparation of 1,8-octanediol diacrylate (ODA) **7**

Figure S2. ^{1}H NMR (400 MHz, $CDCl_3$) spectrum of ODA. 8

Figure S3. ^{13}C NMR (101 MHz, $CDCl_3$) spectrum of ODA. 8

Preparation of 1,12-dodecanediol diacrylate (DoDA) **9**

Figure S4. ^{1}H NMR (400 MHz, $CDCl_3$) spectrum of DoDA. 9

Figure S5. ^{13}C NMR (101 MHz, $CDCl_3$) spectrum of DoDA. 10

Preparation of diethylene glycol diacrylate (GDA) **10**

Figure S6. ^{1}H NMR (400 MHz, $CDCl_3$) spectrum of GDA. 11

Figure S7. ^{13}C NMR (101 MHz, $CDCl_3$) spectrum of GDA. 11

Preparation of *p*-xylylene glycol diacrylate (XDA) **12**

Figure S8. ^{1}H NMR (400 MHz, $CDCl_3$) spectrum of XDA. 12

Figure S9. ^{13}C NMR (101 MHz, $CDCl_3$) spectrum of XDA. 13

Preparation of bisphenol A diacrylate (BPDA) **13**

Figure S10. ^{1}H NMR (400 MHz, $CDCl_3$) spectrum of BPDA. 14

Figure S11. ^{13}C NMR spectrum (101 MHz, $CDCl_3$) of BPDA. 14

Preparation of [(dimethylsilylene)dioxy] diacrylate (SiDA) **15**

Figure S12. ^{1}H NMR (400 MHz, $CDCl_3$) spectrum of SiDA. 15

Figure S13. ^{13}C NMR (101 MHz, $CDCl_3$) spectrum of SiDA. 16

Preparation of 1,4-butanediol diacrylate urethane (BDDU) **16**

Figure S14. ^{1}H NMR (400 MHz, $CDCl_3$) spectrum of BDDU. 17

Figure S15. ^{13}C NMR (101 MHz, $CDCl_3$) spectrum of BDDU. 17

Polymerization of DAs **18**

Figure S16. Overlaid ^{1}H NMR spectra of the reaction mixture of polymerization in toluene at 2 hours by using (a) PPh_3; (b) $P(NMe_2)_3$; (c) PCy_3 as the catalyst. The conversion of the C═C double

bonds was calculated from the integral ratio of characteristic peaks: Conv. (%) = [I(a')/(I(a)+I(a'))]×100% 18
Figure S17. ^{1}H NMR (400 MHz, $CDCl_3$) spectrum of PBDA (Entry 9, Table 1). 19
Figure S18. ^{13}C NMR (101 MHz, $CDCl_3$) spectrum of PBDA (Entry 9, Table 1). 19
Figure S19. ^{1}H-^{1}H COSY NMR ($CDCl_3$) spectrum of PBDA (Entry 9, Table 1). 20
Figure S20. ^{1}H-^{13}C HSQC NMR ($CDCl_3$) spectrum of PBDA (Entry 9, Table 1). 20
Figure S21. ^{31}P NMR (162 MHz, $CDCl_3$) spectrum of PBDA (Entry 9, Table 1). 21
Figure S22. Possible mechanism of gelation by the formation of insoluble crosslinking network when the conversion of BDA approached slightly below 100%. 21
Figure S23. ^{1}H NMR (400 MHz, $CDCl_3$) spectrum of PHDA (Entry 2, Table 2). 22
Figure S24. ^{13}C NMR (101 MHz, $CDCl_3$) spectrum of PHDA (Entry 2, Table 2). 22
Figure S25. (a) MALDI-TOF mass spectrum of PBu_3-catalyzed HDA (Entry 1, Table 2). (b) MALDI-TOF mass spectrum of PCy_3-catalyzed HDA (Entry 2, Table 2). 23
Figure S26. ^{1}H NMR (400 MHz, $CDCl_3$) spectrum of PODA (Entry 4, Table 2). 23
Figure S27. ^{13}C NMR (101 MHz, $CDCl_3$) spectrum of PODA (Entry 4, Table 2). 24
Figure S28. ^{1}H NMR (400 MHz, $CDCl_3$) spectrum of PDoDA (Entry 6, Table 2). 24
Figure S29. ^{13}C NMR (101 MHz, $CDCl_3$) spectrum of PDoDA (Entry 6, Table 2). 25
Figure S30. ^{1}H NMR (400 MHz, $CDCl_3$) spectrum of PGDA (Entry 11, Table 2). 25
Figure S31. ^{13}C NMR (101 MHz, $CDCl_3$) spectrum of PGDA (Entry 11, Table 2). 26
Figure S32. ^{1}H NMR (400 MHz, $CDCl_3$) spectrum of PXDA (Entry 9, Table 2). 26
Figure S33. ^{13}C NMR (101 MHz, $CDCl_3$) spectrum of PXDA (Entry 9, Table 2). 27
Figure S34. ^{1}H NMR (400 MHz, $CDCl_3$) spectrum of PBPDA (Entry 10, Table 2). 27
Figure S35. ^{13}C NMR (101 MHz, $CDCl_3$) spectrum of PBPDA (Entry 10, Table 2). 28
Figure S36. ^{1}H NMR (400 MHz, $CDCl_3$) spectrum of PSiPDA (Entry 8, Table 2). 28
Figure S37. ^{13}C NMR (101 MHz, $CDCl_3$) spectrum of PSiDA (Entry 8, Table 2). 29
Figure S38. ^{1}H NMR (400 MHz, $CDCl_3$) spectrum of PBDDU (Entry 12, Table 2). 29
Figure S39. ^{13}C NMR (101 MHz, $CDCl_3$) spectrum of PBDDU (Entry 12, Table 2). 30
Calculations of the Average Molecular Weight Between Crosslinks M_c. 31
Table S1. The swelling ratio Q, the volume fractions of the polymer V_K and the average molecular weight between crosslinks M_c of gelled polymerization mixtures. 31
SEC Characterization of Polymers 32
Figure S40. SEC traces (THF as the eluent) of PBDA obtained from different [monomer]:[PBu_3] ratios (Entries 1, 2, Table 1). 32
Figure S41. SEC traces (THF as the eluent) of PBDA obtained from PBu_3-catalyzed polymerization in different solvents (Entries 1&3–6, Table 1). 32
Figure S42. SEC traces (THF as the eluent) of PBDA obtained from PCy_3-catalyzed polymerization in different solvents (Entries 9–13, Table 1). 33
Figure S43. SEC trace (THF as the eluent) of PBDA obtained from a 20-g scale batch. 33
Figure S44. SEC traces (THF as the eluent) of PHDA (Entries 1, 2, Table 2). 33
Figure S45. SEC traces (THF as the eluent) of PODA (Entries 3, 4, Table 2). 34
Figure S46. SEC traces (THF as the eluent) of PDoDA (Entries 5, 6, Table 2). 34
Figure S47. SEC traces (THF as the eluent) of PSiDA (Entries 7, 8, Table 2). 34
Figure S48. SEC traces (THF as the eluent) of PXDA, PBPDA, PGDA, PBDDU (Entries 9–12, Table 2). 35

Thermogravimetric Analysis (TGA) of Polymers **35**
Figure S49. TGA and DTG curves of PBDA (Entry 9, Table 1). 35
Figure S50. TGA and DTG curves of PHDA (Entry 2, Table 2). 35
Figure S51. TGA and DTG curves of PODA (Entry 4, Table 2). 36
Figure S52. TGA and DTG curves of PDoDA (Entry 6, Table 2). 36
Figure S53. TGA and DTG curves of PSiDA (Entry 8, Table 2). 36
Figure S54. TGA and DTG curves of PXDA (Entry 9, Table 2). 37
Figure S55. TGA and DTG curves of PBPDA (Entry 10, Table 2). 38
Figure S56. TGA and DTG curves of PGDA (Entry 11, Table 2). 38
Figure S57. TGA and DTG curves of PBDDU (Entry 12, Table 2). 38
Post-modification of PBDA and PDoDA **39**
One-Pot Synthesis of PBDA–ME **39**
Photo-Induced Free Radical Synthesis of PBDA–ME **39**
Figure S58. (A) The overlaid ^{1}H NMR (400 MHz, $CDCl_3$) spectra of PBDA and PBDA–ME. (B) ^{1}H NMR (400 MHz, $CDCl_3$) spectrum of PBDA–ME. 40
Figure S59. ^{13}C NMR (101 MHz, $CDCl_3$) spectrum of PBDA–ME. 41
Figure S60. Overlaid ^{1}H NMR spectra (400 MHz, $CDCl_3$) of PBDA–ME acquired from one-pot thiol-Michael addition click reaction and photo-induced free radical click reaction. 41
Figure S61. ^{31}P NMR ($CDCl_3$) of PBDA after quenching with HCl/methanol (0.1 M). 42
Figure S62. Overlaid ^{1}H NMR ($CDCl_3$) spectra of PBDA after removal of the phosphine (bottom) and its reaction mixture with ME after 6 h (top). 42
One-Pot Synthesis of PBDA–BnSH **43**
Photo-Induced Free Radical Synthesis of PBDA–BnSH **43**
Figure S63. Overlaid ^{1}H NMR spectra (400 MHz, $CDCl_3$) of PBDA–BnSH acquired from one-pot thiol-Michael addition click reaction and photo-induced free radical click reaction. 43
Figure S64. ^{1}H NMR (400 MHz, $CDCl_3$) spectrum of PBDA–BnSH. 44
Figure S65. ^{13}C NMR (101 MHz, $CDCl_3$) spectrum of PBDA–BnSH. 44
One-Pot Synthesis of PDoDA–ME **45**
Figure S66. (A) The overlaid ^{1}H NMR (400 MHz, $CDCl_3$) spectra of PDoDA and PDoDA–ME. (B) ^{1}H NMR (400 MHz, $CDCl_3$) spectrum of PDoDA–ME. 45
Figure S67. ^{13}C NMR (101 MHz, $CDCl_3$) spectrum of PDoDA–ME. 46
Synthesis of PDoDA1 and PDoDA2 .... **46**
Figure S68. SEC traces (THF as the eluent) of PDoDA1 and PDoDA2. 46
Preparation of Bottlebrush Copolymers PDoDA-*g*-PLLA$_m$ **47**
Figure S69. Overlaid ^{1}H NMR spectra (400 MHz, $CDCl_3$) of PDoDA–ME and PDoDA-*g*-PLLA$_m$. The disappearance of the peak at 3.72 ppm suggested the grafting density is close to 100%. 47
Preparation of PLLA Homopolymer **47**
Figure S70. Tensile tests for PDoDA-*g*-PLLA$_{188}$. 48
Figure S71. Tensile tests for PDoDA-*g*-PLLA$_{465}$. 48
Figure S72. Tensile tests for PBDA-*graft*-PLLA$_{924}$. 48
Figure S73. Tensile tests for PLLA homopolymer. 49
Figure S74. Tensile tests for PDoDA1-*g*-PLLA$_{953}$ and PDoDA2-*g*-PLLA$_{962}$. 49
Figure S75. DSC curves of PDoDA-*g*-PLLA$_{188}$. 49
Figure S76. DSC curves of PDoDA-*g*-PLLA$_{465}$. 50

Figure S77. DSC curves of PDoDA-*g*-PLLA$_{924}$. 50
Figure S78. DSC curves of homopolymer PLLA. 50
Figure S79. DSC curves of PDoDA1-*g*-PLLA$_{953}$ and PDoDA2-*g*-PLLA$_{962}$. 51
Table S2. The comparison of mechanical and thermal properties for PLLA and graft polymers PDoDA-*g*-PLLA$_m$ with different main-chain lengths. 51
References 51

Experimental Details

Materials

All synthesis and manipulations of air- and moisture-sensitive chemicals and materials were carried out in flamed Schlenk-type glassware on a dual-manifold Schlenk line or in an inert gas (Ar)-filled glovebox. Toluene (Tol) was freshly refluxed over sodium and distilled under nitrogen atmosphere, then stored in glovebox. Anhydrous tetrahydrofuran (THF), dichloromethane (DCM), 1,4-dioxane and acetonitrile (MeCN) were purchased from J&K and used as received. Tricyclohexyl phosphine (Energy Chemical), tributylphosphine (J&K), tri-tert-butylphosphine (Heowns), triphenylphosphine (Aladdin), trimethyl phosphite (Energy Chemical), hexamethylphosphorous triamide (Energy Chemical), tris(4-methoxyphenyl)phosphine (Accela Chembio Co.), acryloyl chloride (Energy Chemical) 1,4-butanediol (J&K), 1,8-octanediol (Aladdin), 1,12-dodecanediol (Konoscience), tert-butanol (J&K), 2-hydroxyethyl acrylate (Konoscience), *p*-xylylene glycol (TCI), bisphenol A (Energy Chemical), dichlorodimethylsilane (Aladdin), 2-isocyanatoethyl acrylate (Energy Chemical), diethylene glycol (Energy Chemical), triethylamine (TEA, TGREAG), 2-mercaptoethanol (Energy Chemical), benzyl mercaptan (Energy Chemical), 2,2-dimethoxyl-2-phenylacetophenone (DMPA, Bidepharm), stannous octoate ($Sn(Oct)_2$, J&K) were purchased from commercial suppliers and used as received. BDA and HDA monomers were purchased from Konoscience, dried by stirring over CaH_2 and then distilled under vacuum. ODA, SiDA and GDA monomers were prepared according to the literature, [1,2] dried by stirring over CaH_2 and distilled under vacuum. DoDA, XDA, BPDA and BDDU monomers were prepared according to the literature, [1,3] purified by recrystallization from the DCM/pentane and sublimation under vacuum. L-lactide was purchased from J&K and recrystallized in dry toluene and then sublimated.

Methods

Nuclear Magnetic Resonance (NMR)

^{1}H, ^{13}C, ^{1}H-^{13}C HSQC, ^{31}P and DOSY NMR spectra were measured on a Bruker Avance III 400 MHz or 500 MHz spectrometer. Chemical shifts are reported in δ (ppm) relative to the residual solvent peak. Splitting patterns are designated as singlet (s), doublet (d), triplet (t), quartet (q), doublet of doublets (dd), and multiplet (m).

Size Exclusion Chromatography (SEC)

SEC measurements were performed on an Agilent 1260 Infinity II instrument equipped with two Agilent PLgel columns (MIXED-C, MIXED-D) and a RI detector (THF as eluent), or an Agilent LC system equipped with one guard column (50×8.0 mm, 5 um) and two MZ-Gel LS SDplusLinear columns (300×8.0 mm, 5 um) and coupled with a Wyatt Optilab refractive index detector ($CHCl_3$ as eluent). HPLC grade THF or $CHCl_3$ was used as the eluent with a flow rate of 1.0 mL/min at 35 °C. M_n and $Đ$ were obtained on Agilent software under a calibration curve from polystyrene standards. The chromatograms were processed with Agilent software or Wyatt ASTRA software.

Matrix-Assisted Laser Desorption Ionization Time-of-Flight Mass Spectroscopy (MALDI-TOF MS)

MALDI-TOF measurements were performed on a MALDI TOF/TOF 5800 mass spectrometer (AB SCIEX). Trans-2-[3-(4-tert-butylphenyl)-2-methyl-2-propenylidene] malononitrile (DCTB) was used as the matrix, and the spectrum was acquired in positive reflection mode. Samples were prepared by mixing 3 μL of polymer sample (10 mg/mL in THF or DCM), 1 μL of CF_3COONa (10 mg/mL in THF), and 20 μL of DCTB (20 mg/mL in THF). Then, 0.5 μL of the mixed solution was spotted on the sample plate

and allowed to be air-dried before a test.

Fourier Transform Infrared Spectroscopy (FTIR)

FTIR spectra in the 4000–400 cm^{-1} range were recorded on a Bruker Tensor 27 FTIR spectrometer using KBr plates.

Thermogravimetric Analysis (TGA)

TGA measurements were performed on a Q600 SDT thermogravimetric analyzer. Samples were heated from ambient temperature to 600 °C at a heating rate of 10 °C/min under a nitrogen flow of 100 mL/min. Decomposition temperatures (T_d) were defined at a 5% weight loss. Decomposition temperatures (T_d, max) were obtained from derivative (wt%/°C) vs. temperature (°C) plots.

Differential Scanning Calorimetry (DSC)

DSC measurements were performed on a TA DSC250 differential scanning calorimeter under a nitrogen flow of 50 mL/min at a heating and cooling rate of 10 °C/min (unless otherwise specified). All T_m and T_g values were obtained from a second scan after the thermal history was removed from the first scan.

Tensile Tests

Tensile stress/strain testing was performed by an Instron 3365 universal testing system on dog-bone-shaped test specimen generated via slow-solvent evaporation. The dogbone specimen were annealed in a 50 °C oven overnight. Test specimens were stretched at a strain rate of 10 mm/min at ambient temperature unless indicated otherwise until break. The measurements repeated 3 times and the values reported are averaged from the measured data.

Synthesis of Monomers

ODA, DoDA, XDA, BPDA and GDA were prepared from the corresponding diols and acryloyl chloride. SiDA was prepared through the substitution reaction of dichlorodimethylsilane and 2-hydroxyethyl acrylate. BDDU was synthesized through the addition reaction of 1, 4-butanediol to 2-isocyanatoethyl acrylate.

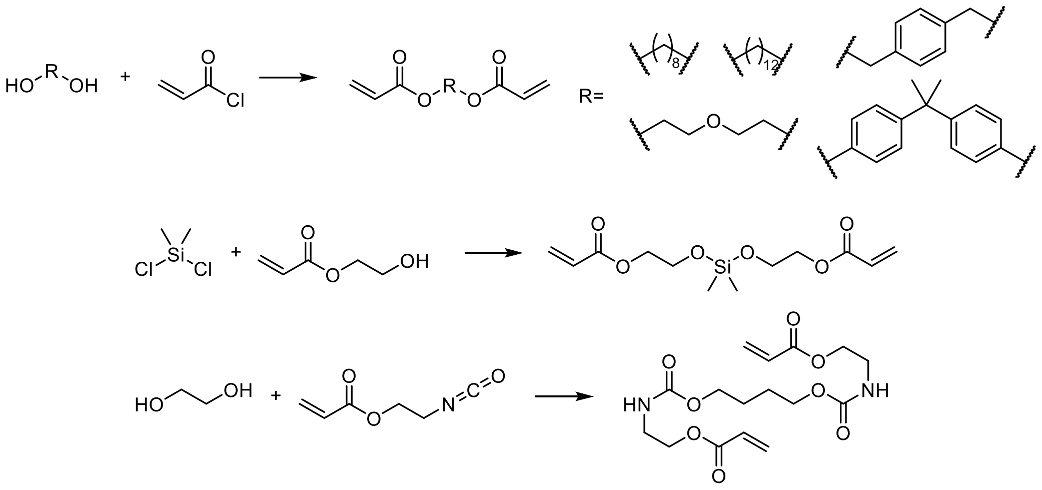

Figure S1. Synthesis of diacrylate monomers.

Preparation of 1,8-octanediol diacrylate (ODA)

According to the literature, [1] 1,8-octanediol (7.13 g, 50 mmol) and TEA (28.8 mL, 200 mmol) were dissolved in DCM (100 mL) and underwent three freeze-pump-thaw cycles. Acryloyl chloride (16 mL, 200 mmol) was diluted with DCM (50 mL) and added dropwise at 0 °C under stirring in N_2 atmosphere within 30 minutes. The reaction mixture was stirred at 0 °C for 2 hours and then at room temperature for 16 hours. The reaction mixture was filtered to remove ammonium salt and then washed with water (2×300 mL) and brine (300 mL) and dried over Na_2SO_4, filtered and concentrated under vacuo. The obtained residue was purified by silica-gel column chromatography (petroleum ether: ethyl acetate = 5:1) to afford colorless liquid (71% yield). ^{1}H NMR (400 MHz, $CDCl_3$), δ (ppm): 6.40 (2H, dd, ^2J = 1.6 Hz, ^3J = 17.3 Hz), 6.12 (2H, dd, ^3J = 10.4, 17.3 Hz), 5.82 (2H, dd, ^2J = 1.5 Hz, ^3J = 10.7 Hz), 4.15 (4H, t, J = 6.7 Hz), 1.66 (4H, m), 1.41–1.33 (8H, m). ^{13}C NMR (101 MHz, $CDCl_3$), δ (ppm): 166.34, 130.45, 128.63, 64.62, 29.10, 28.57, 25.83.

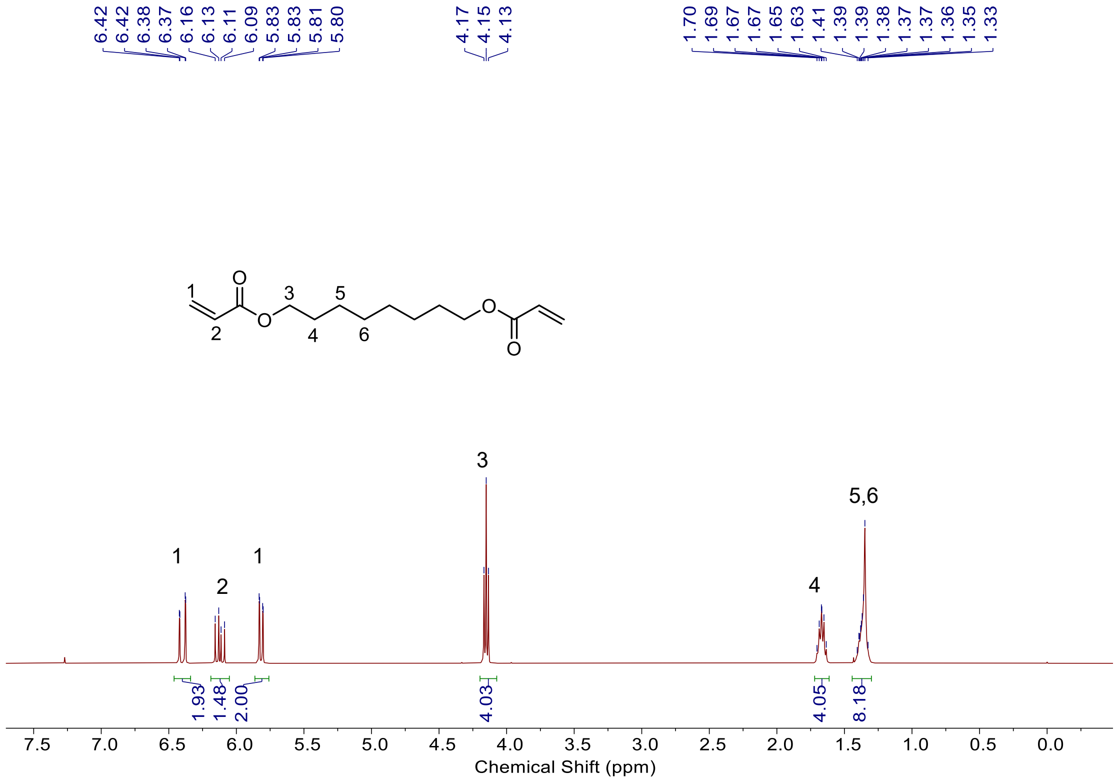

Figure S2. ^{1}H NMR (400 MHz, $CDCl_3$) spectrum of ODA.

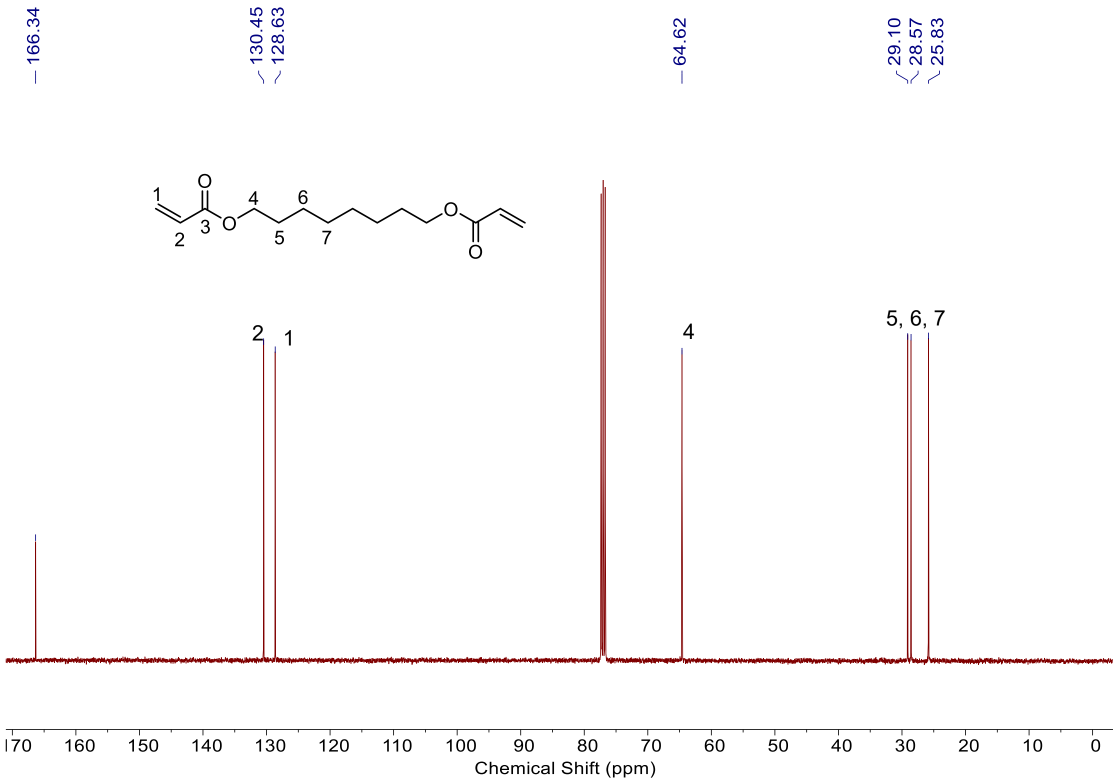

Figure S3. ^{13}C NMR (101 MHz, $CDCl_3$) spectrum of ODA.

Preparation of 1,12-dodecanediol diacrylate (DoDA)

Similar to the preparation of ODA, 1,12-dodecanediol (10.12 g, 50 mmol) and TEA (28.8 mL, 200 mmol) were dissolved in THF (100 mL) and underwent three freeze-pump-thaw cycles. Acryloyl chloride (16 mL, 200 mmol) was diluted with THF (50 mL) and added dropwise at 0 °C under stirring in N_2 atmosphere within 30 minutes. The reaction mixture was stirred at 0 °C for 2 hours and then at room temperature for 24 hours. The reaction mixture was filtered to remove ammonium salt. The solvent was removed under vacuo, then the residue was diluted with DCM (200 mL), washed with water (2×300 mL) and brine (300 mL) and dried over Na_2SO_4, filtered and concentrated under vacuo. The obtained residue was purified by silica-gel column chromatography (petroleum ether: ethyl acetate = 10:1) to afford white crystal (64% yield). ^{1}H NMR (400 MHz, $CDCl_3$), δ (ppm): 6.39 (2H, dd, ^2J = 1.5 Hz, ^3J=17.3 Hz), 6.11 (2H, dd, ^3J = 10.4, 17.3 Hz), 5.80 (2H, dd, ^2J = 1.5 Hz, ^3J = 10.4 Hz), 4.14 (4H, t, J = 6.7 Hz), 1.65 (4H, m), 1.36–1.27 (16H, m). ^{13}C NMR (101 MHz, $CDCl_3$), δ (ppm): 166.34, 130.39, 128.67, 64.70, 29.51, 29.48, 29.24, 28.61, 25.92.

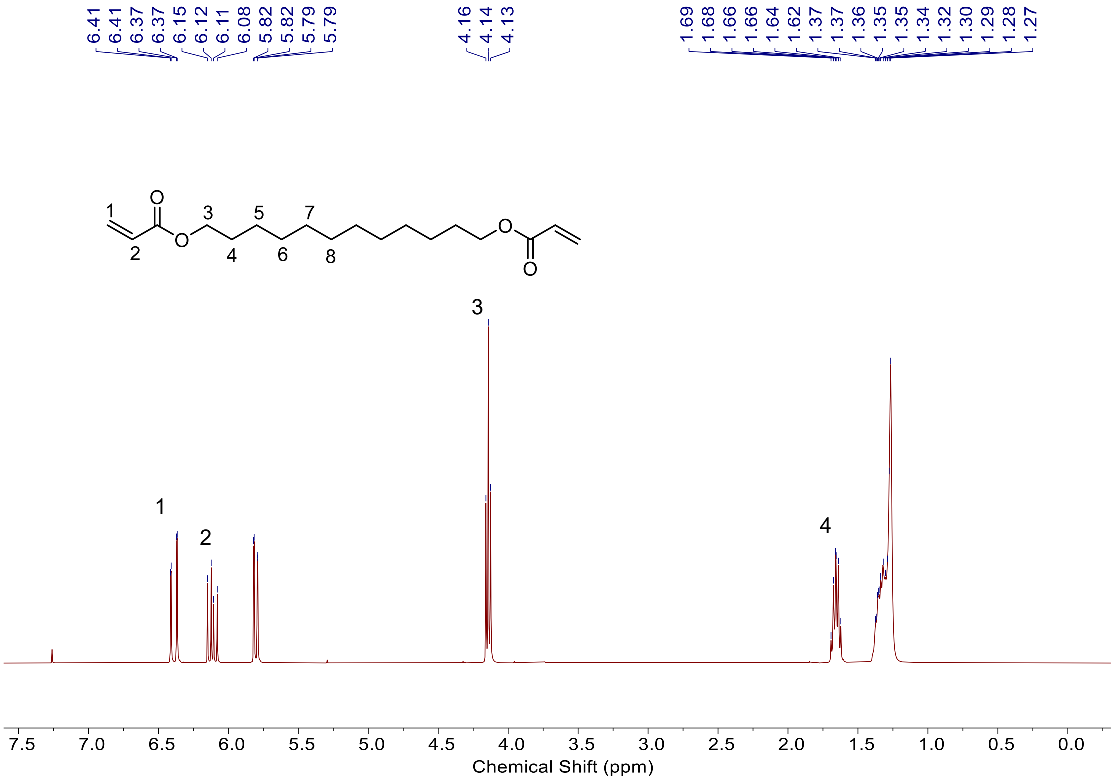

Figure S4. ^{1}H NMR (400 MHz, $CDCl_3$) spectrum of DoDA.

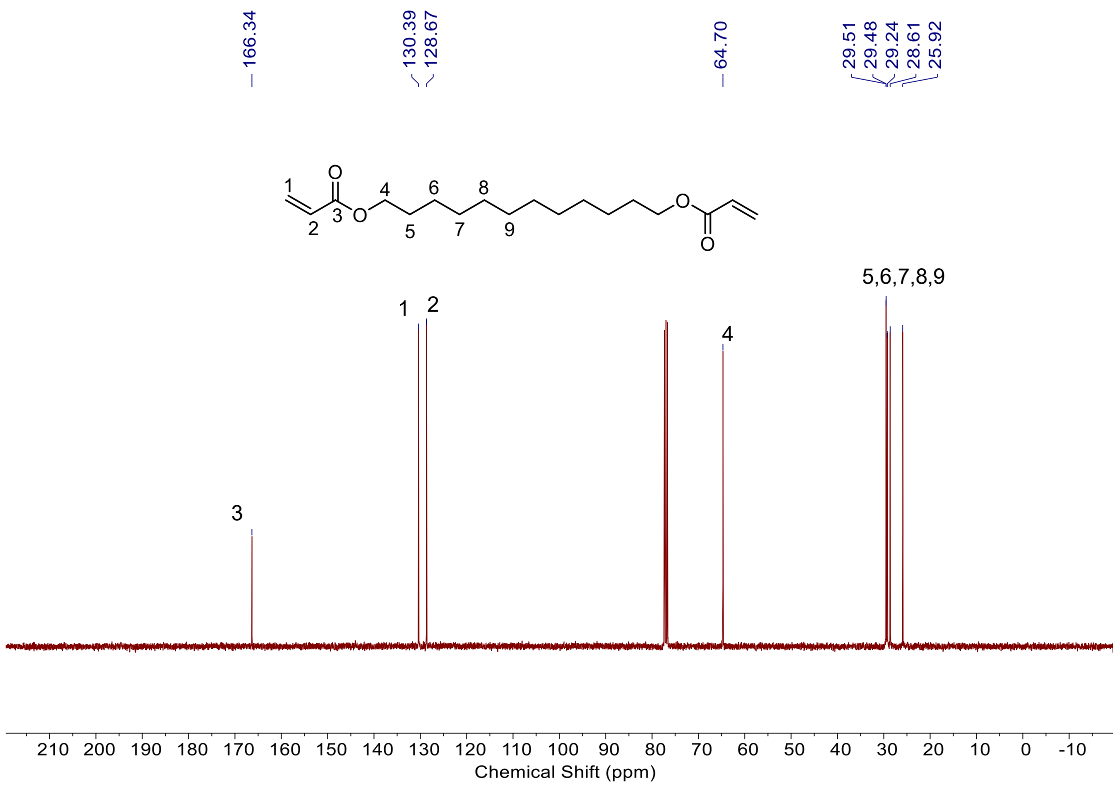

Figure S5. ^{13}C NMR (101 MHz, $CDCl_3$) spectrum of DoDA.

Preparation of diethylene glycol diacrylate (GDA)

Similar to the preparation of ODA, diethylene glycol (5.3 g, 50 mmol) and TEA (28.8 mL, 200 mmol) were dissolved in DCM (100 mL) and underwent three freeze-pump-thaw cycles. Acryloyl chloride (16 mL, 200 mmol) was diluted with DCM (50 mL) and added dropwise at 0 °C under stirring in N_2 atmosphere within 30 minutes. The reaction mixture was stirred at 0 °C for 2 hours and then at room temperature for 18 hours. The reaction mixture was filtered to remove ammonium salt and then washed with water (2×300 mL) and brine (300 mL) and dried over Na_2SO_4, filtered and concentrated under vacuo. The obtained residue was purified by silica-gel column chromatography (petroleum ether: ethyl acetate = 3:1) to afford colorless liquid (80% yield). ^1H NMR (400 MHz, $CDCl_3$), δ (ppm): 6.43 (2H, dd, ^2J = 1.4 Hz, ^3J = 17.4 Hz), 6.15 (2H, dd, ^3J = 10.4, 17.3 Hz), 5.84 (2H, dd, ^2J = 1.4 Hz, ^3J=10.4 Hz), 4.32 (4H, t, J = 4.9 Hz), 3.75 (4H, t, J = 4.8 Hz), 1.41–1.33 (8H, m). ^{13}C NMR (101 MHz, $CDCl_3$), δ (ppm): 166.23, 131.22, 128.33, 69.19, 63.67.

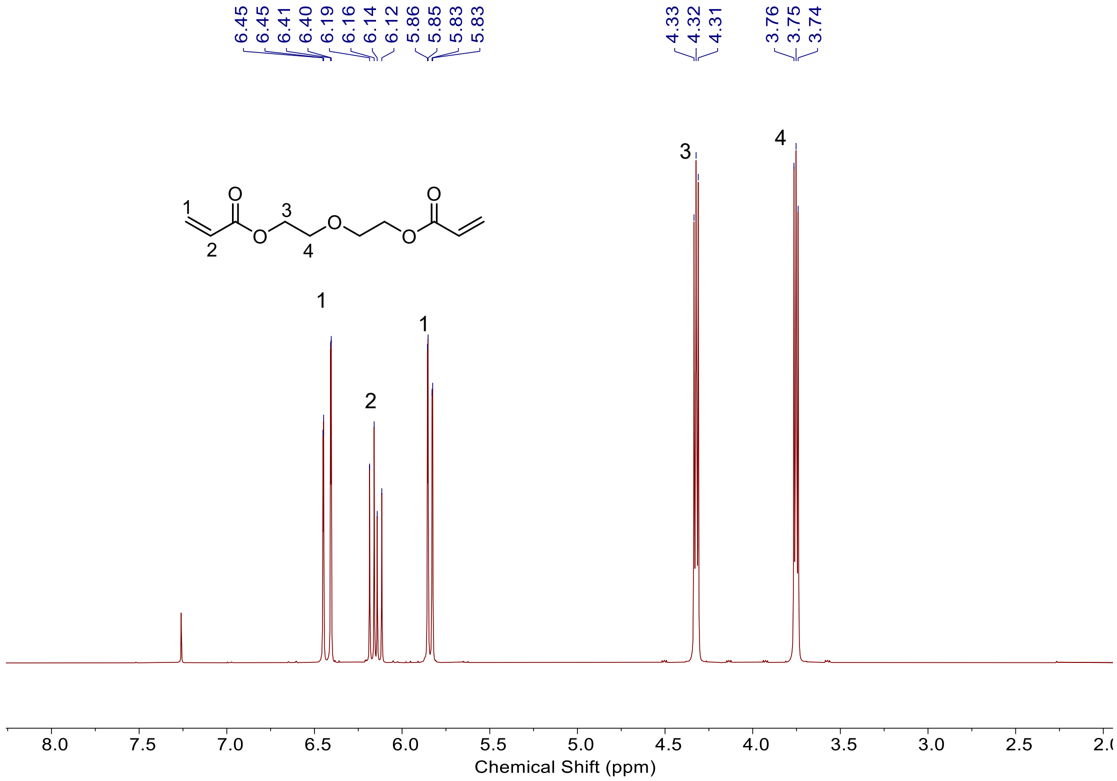

Figure S6. ^{1}H NMR (400 MHz, $CDCl_3$) spectrum of GDA.

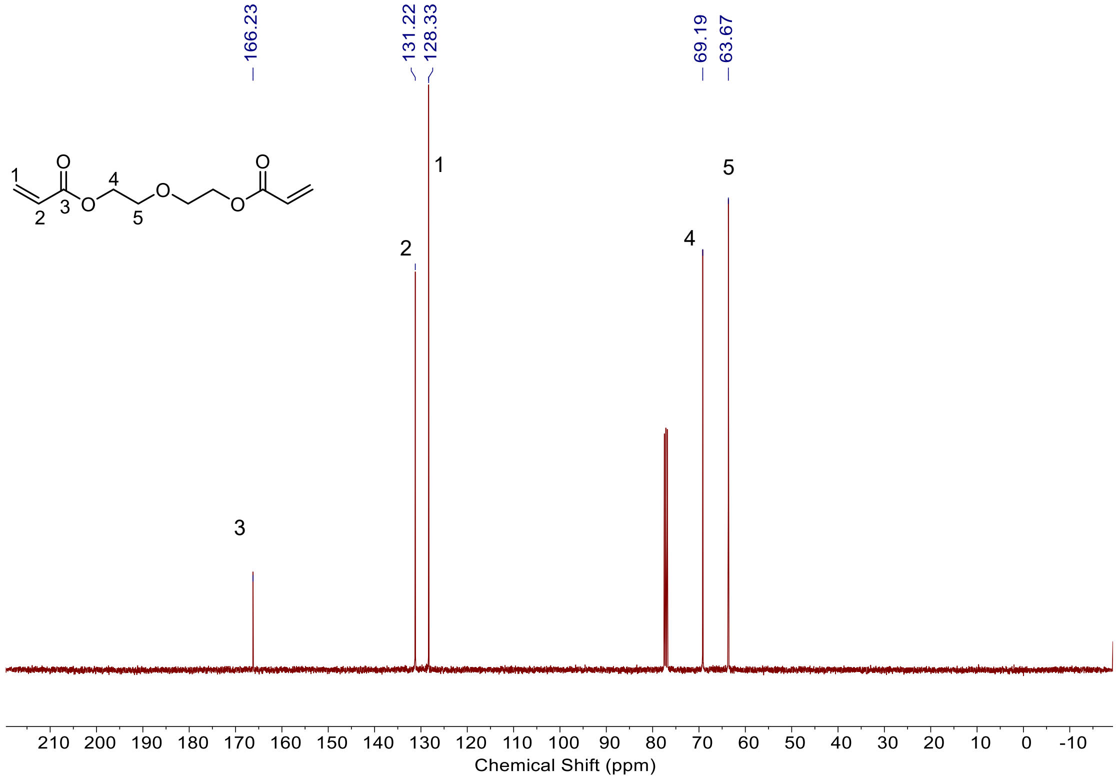

Figure S7. ^{13}C NMR (101 MHz, $CDCl_3$) spectrum of GDA.

Preparation of *p*-xylylene glycol diacrylate (XDA)

Similar to the preparation of ODA, *p*-Xylylene glycol (4.14 g, 30 mmol) and TEA (17.0 mL, 120 mmol) were dissolved in DCM (100 mL) and underwent three freeze-pump-thaw cycles. Acryloyl chloride (5.1 mL, 63 mmol) was diluted with DCM (50 mL) and added dropwise at 0 °C under stirring in N_2 atmosphere within 30 minutes. The reaction mixture was stirred at 0 °C for 2 hours and then at room temperature for 16 hours. The reaction mixture was filtered to remove ammonium salt and then washed with water (2×300 mL) and brine (300 mL) and dried over Na_2SO_4, filtered and concentrated under vacuo. The obtained residue was purified by silica-gel column chromatography (petroleum ether: ethyl acetate = 5:1) to afford white crystal (60% yield). ^{1}H NMR (400 MHz, $CDCl_3$), δ (ppm): 7.39 (4H, s), 6.45 (2H, dd, ^2J = 1.5 Hz, ^3J = 17.4 Hz), 6.16 (2H, dd, ^3J = 10.4, 17.3 Hz), 5.85 (2H, dd, ^2J = 1.4 Hz, ^3J=10.4 Hz), 5.20 (4H, s). ^{13}C NMR (101 MHz, $CDCl_3$), δ (ppm): 166.10, 136.12, 131.35, 128.60, 128.37, 66.07.

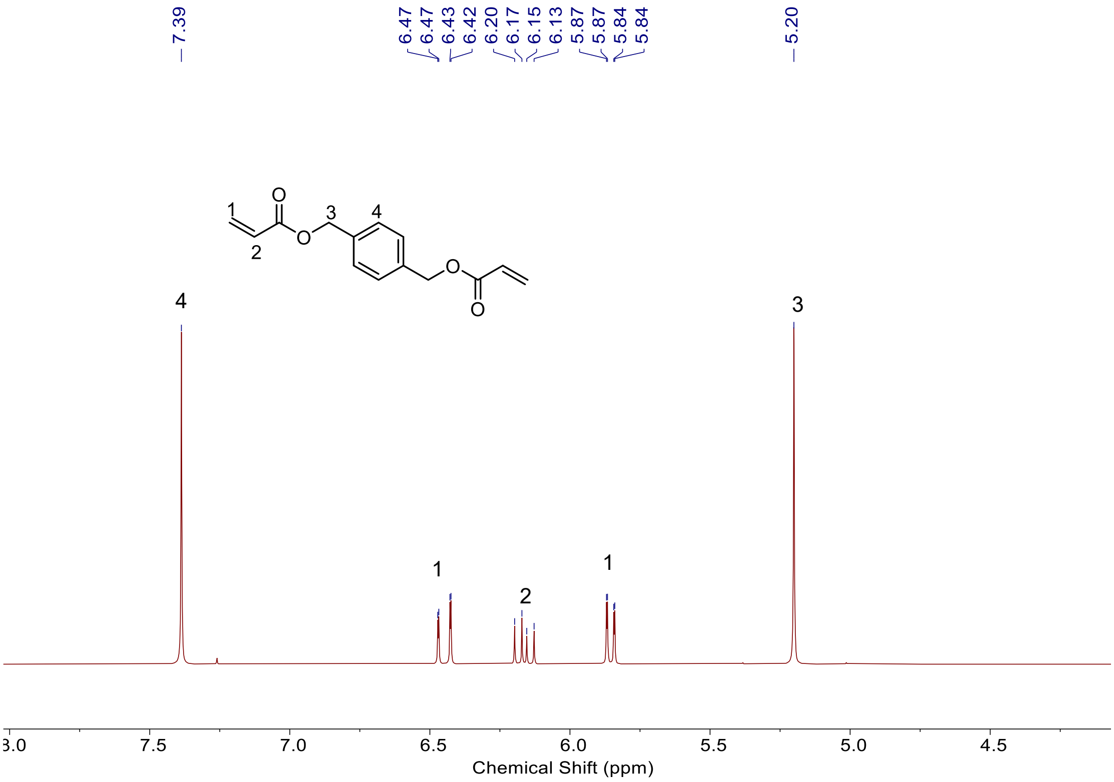

Figure S8. ^{1}H NMR (400 MHz, $CDCl_3$) spectrum of XDA.

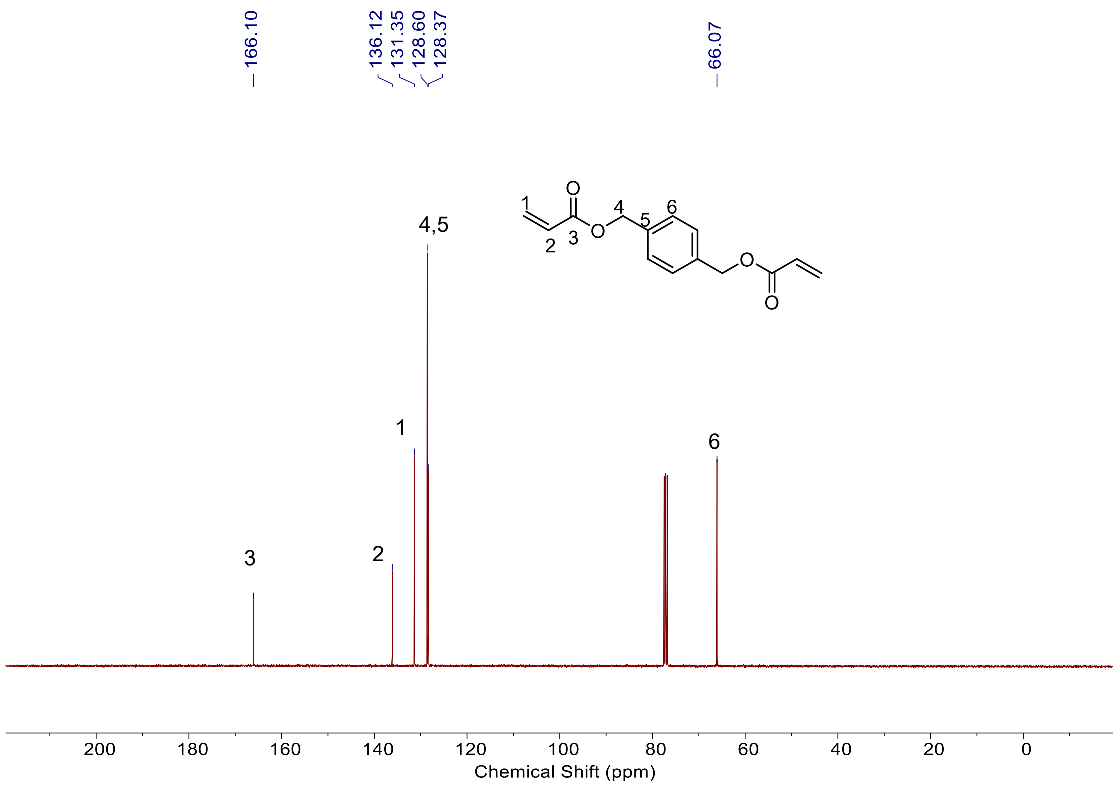

Figure S9. ^{13}C NMR (101 MHz, $CDCl_3$) spectrum of XDA.

Preparation of bisphenol A diacrylate (BPDA)

Similar to the preparation of ODA, Bisphenol A (11.4 g, 50 mmol) and TEA (28.8 mL, 200 mmol) were dissolved in DCM (100 mL) and underwent three freeze-pump-thaw cycles. Acryloyl chloride (16 mL, 200 mmol) was diluted with DCM (50 mL) and added dropwise at 0 °C under stirring in N_2 atmosphere within 30 minutes. The reaction mixture was stirred at 0 °C for 2 hours and then at room temperature for 24 hours. The reaction mixture was filtered to remove ammonium salt and then washed with water (2×300 mL) and brine (300 mL) and dried over Na_2SO_4, filtered and concentrated under vacuo. The obtained residue was purified by silica-gel column chromatography (petroleum ether: ethyl acetate = 5:1) to afford white crystal (81% yield). ^{1}H NMR (400 MHz, $CDCl_3$), δ (ppm): 7.29–7.26 (4H, m), 7.08–7.05 (4H, m), 6.62 (2H, dd, ^2J = 1.3 Hz, ^3J = 17.4 Hz), 6.34 (2H, dd, ^3J = 10.4, 17.3 Hz),6.02 (2H, dd, ^2J = 1.3 Hz, ^3J=10.5 Hz), 1.71 (6H, s). ^{13}C NMR (101 MHz, $CDCl_3$), δ (ppm): 164.74, 148.61, 148.06, 132.56, 128.16, 127.98, 121.02, 42.65, 31.09.

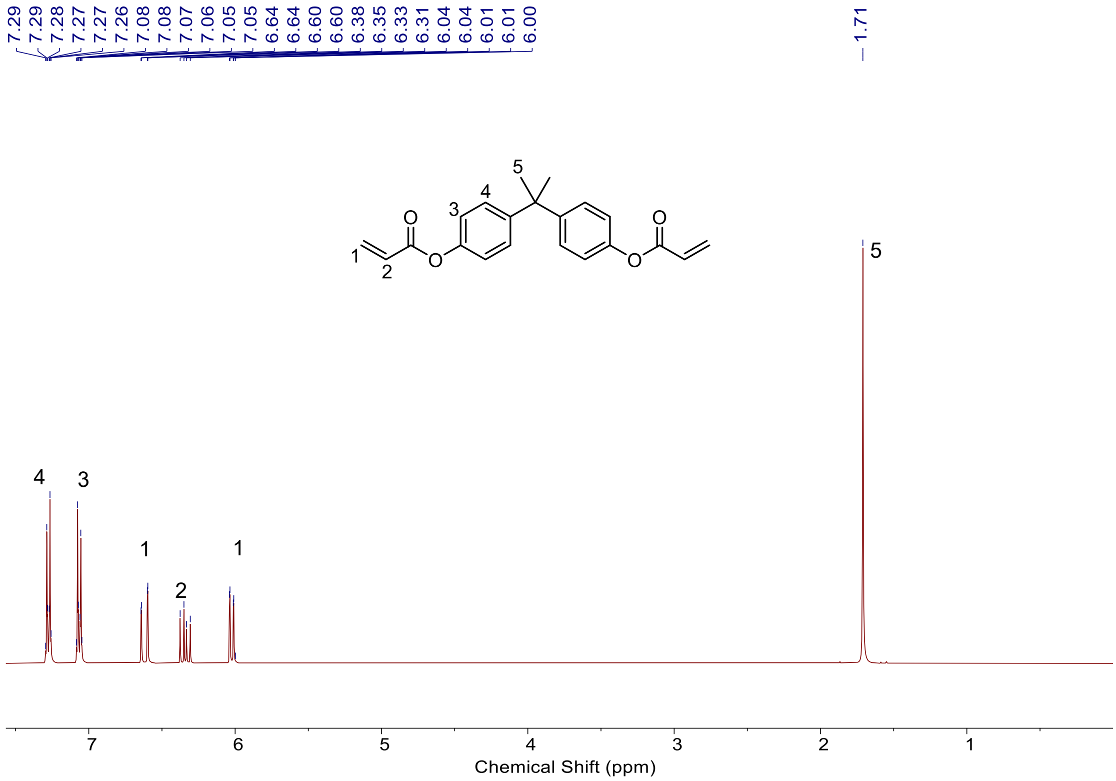

Figure S10. ^1H NMR (400 MHz, $CDCl_3$) spectrum of BPDA.

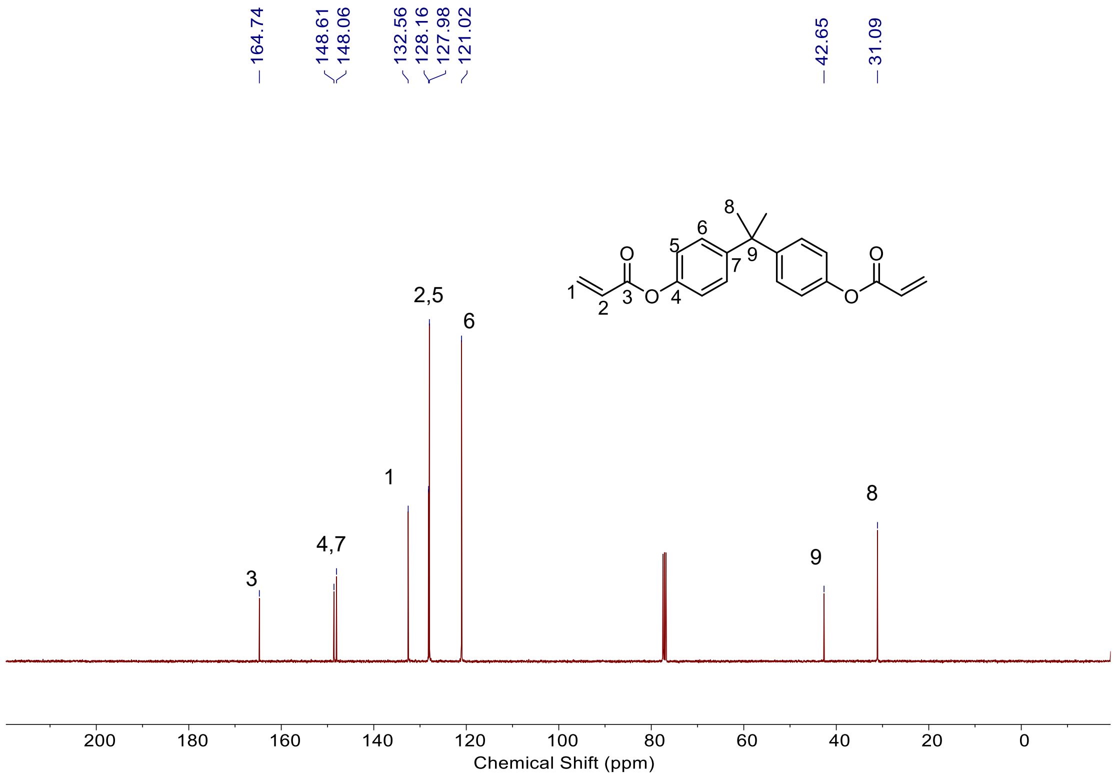

Figure S11. ^{13}C NMR spectrum (101 MHz, $CDCl_3$) of BPDA.

Preparation of [(dimethylsilylene)dioxy] diacrylate (SiDA)

According to the literature, [2] 2-hydroxyethyl acrylate (24.4 g, 210 mmol) and TEA (30 mL, 210 mmol) were dissolved in DCM (100 mL) and underwent three freeze-pump-thaw cycles. Dichlorodimethylsilane (9.7 mL, 100 mmol) was diluted with DCM (20 mL) and added dropwise at 0 °C under stirring in N_2 atmosphere within 30 minutes. The reaction mixture was stirred at 0 °C for 2 hours and then at room temperature for 24 hours. The reaction mixture was filtered to remove ammonium salt and then concentrated under vacuo. The obtained residue was purified by silica-gel column chromatography (petroleum ether: ethyl acetate = 5:1) to afford colorless liquid (65% yield). ^{1}H NMR (400 MHz, $CDCl_3$), δ (ppm): 6.42 (2H, dd, ^2J = 1.0 Hz, ^3J = 17.4 Hz), 6.14 (2H, dd, ^3J = 10.4, 17.4 Hz),5.83 (2H, dd, ^2J = 1.4 Hz, ^3J = 10.5 Hz), 4.24 (4H, t, J = 4.8 Hz), 3.91 (4H, t, J = 4.8 Hz), 0.15 (6H, s). ^{13}C NMR (101 MHz, $CDCl_3$), δ (ppm): 166.25, 131.14, 128.41, 65.64, 60.70, -3.07.

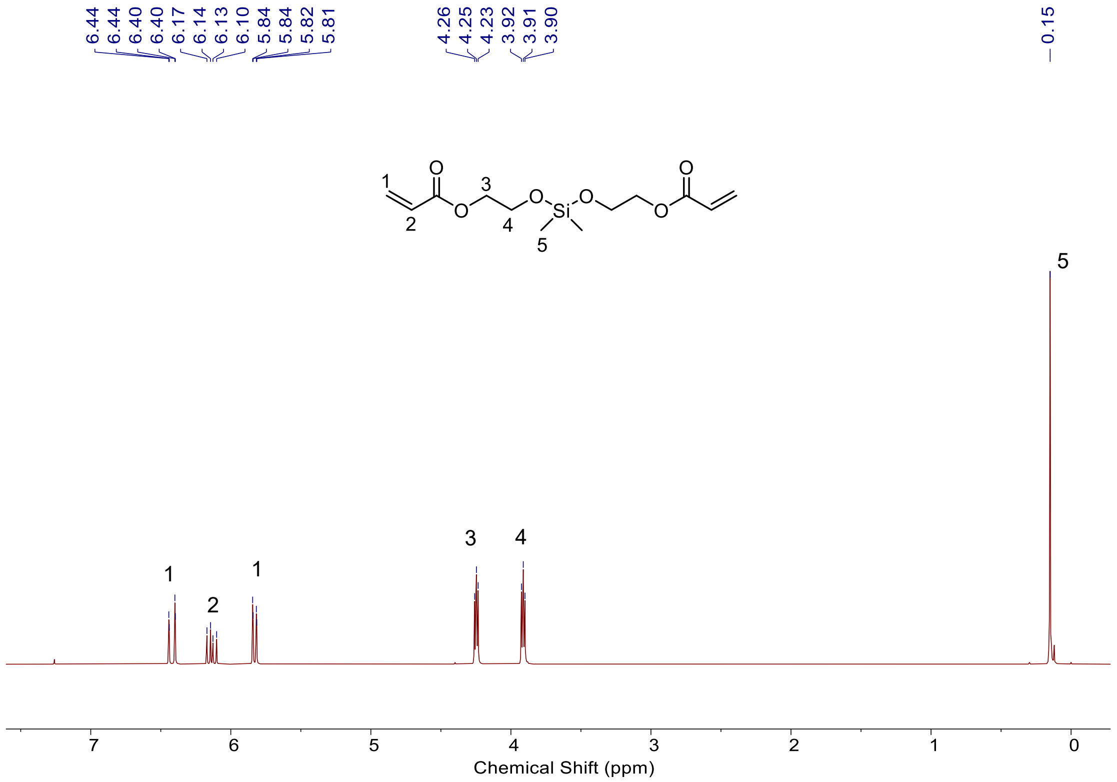

Figure S12. ^{1}H NMR (400 MHz, $CDCl_3$) spectrum of SiDA.

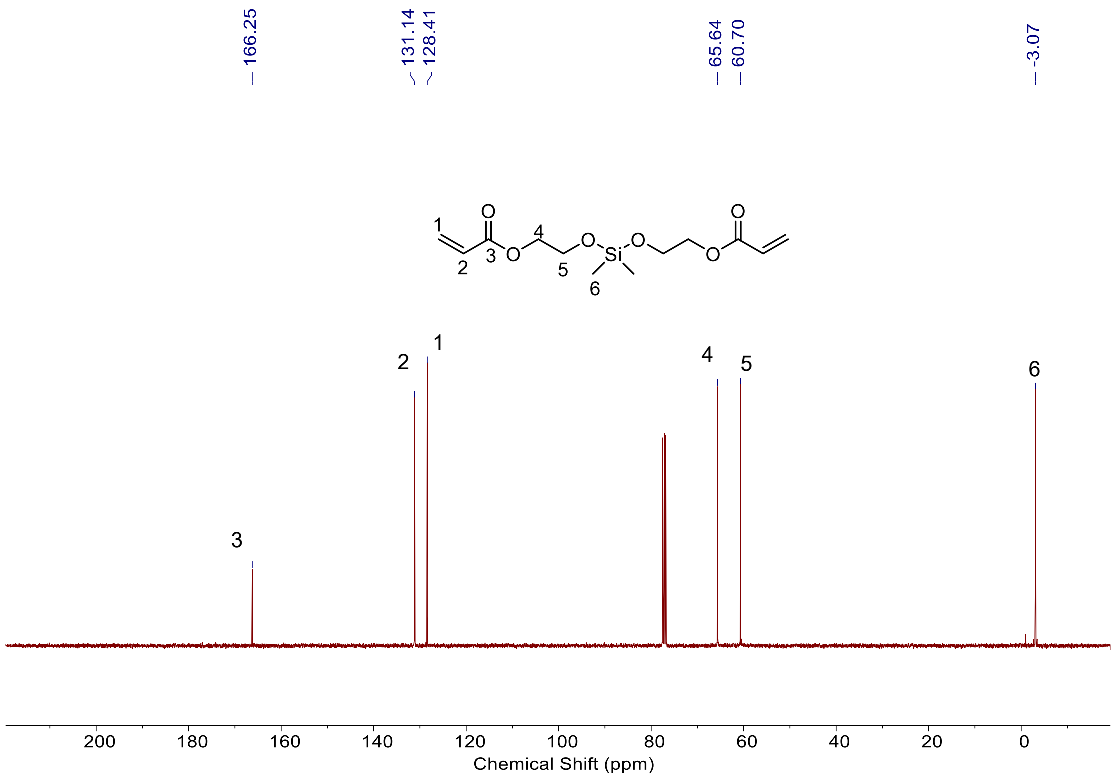

Figure S13. ^{13}C NMR (101 MHz, $CDCl_3$) spectrum of SiDA.

Preparation of 1,4-butanediol diacrylate urethane (BDDU)

According the literature, [3] 1,4-butanediol (2.25 g, 25 mmol) was dissolved in THF (40 mL). 2-Isocyanatoethyl acrylate (7.5 g, 53 mmol) was added dropwise at 0 °C under stirring. Then, $Sn(oct)_2$ (30 μL) was added at 0 °C under stirring. The reaction mixture was left to stir at room temperature for 18 hours. The reaction mixture was concentrated under vacuo and recrystallized from ethyl acetate/pentane to afford white crystal (82% yield). ^1H NMR (400 MHz, $CDCl_3$), δ (ppm): 6.44 (2H, dd, ^2J = 1.4 Hz, ^3J = 17.3 Hz), 6.13 (2H, dd, ^3J = 10.4, 17.3 Hz), 5.87 (2H, dd, ^2J = 1.4 Hz, ^3J = 10.4 Hz), 5.03 (2H, s), 4.24 (4H, t, J = 5.3 Hz), 4.09 (4H, d, J = 5.7 Hz), 3.49 (4H, q, J = 5.6 Hz), 1.69 (4H, m). ^{13}C NMR (101 MHz, $CDCl_3$), δ (ppm): 166.07, 156.55, 131.42, 127.99, 64.60, 63.57, 40.09, 25.61.

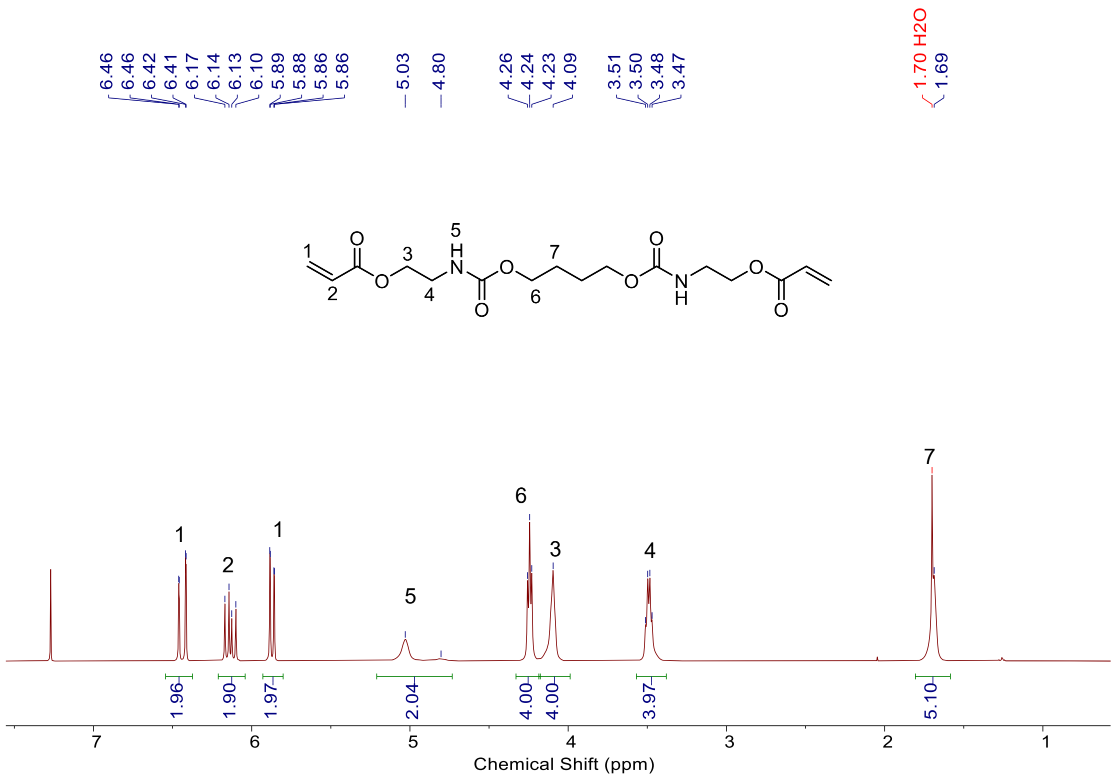

Figure S14. ^{1}H NMR (400 MHz, $CDCl_3$) spectrum of BDDU.

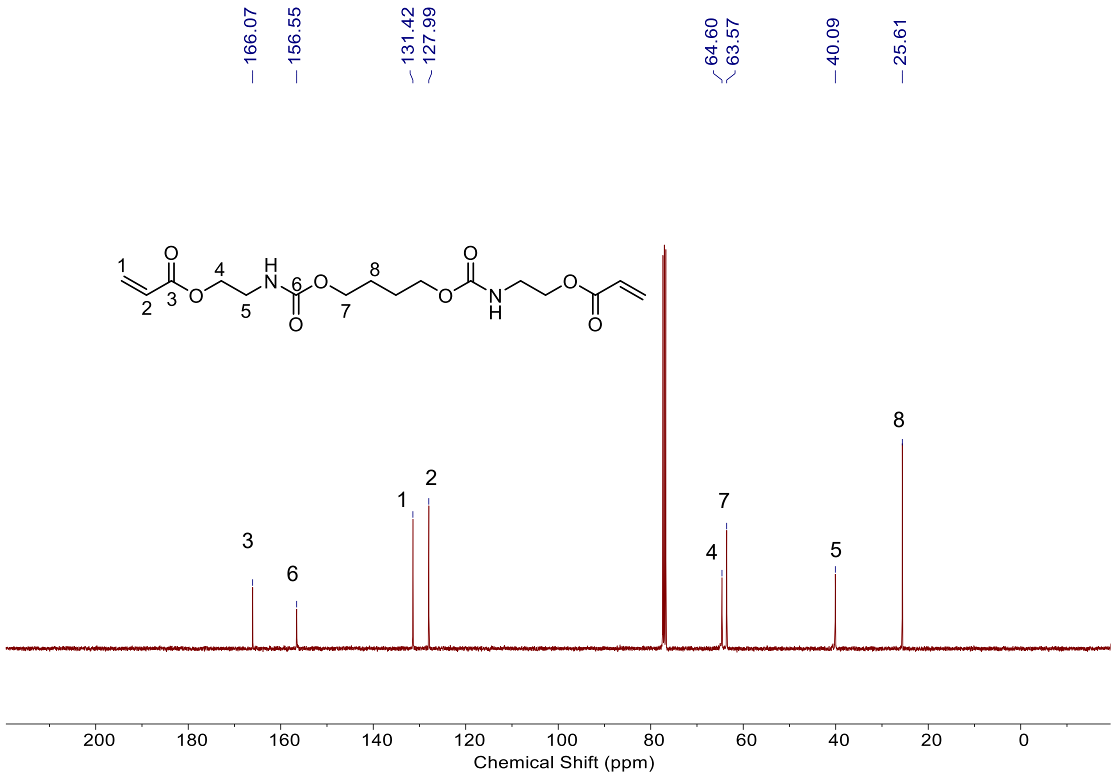

Figure S15. ^{13}C NMR (101 MHz, $CDCl_3$) spectrum of BDDU.

Polymerization of DAs

Taking the polymerization of BDA as a representative example (Entry 9, Table 1). In an argon-filled glovebox, BDA (99 mg, 0.5 mmol) was dissolved in toluene (190 μL) in a 5 mL vial containing a stir bar, then PCy_3 (50 μL, 0.5 mol·L^{-1} in toluene) was added to the above solution to set the initial concentration of monomer to 1.5 mol L^{-1} and [monomer]:[catalyst] ratio to 20:1. The vial was sealed and left to stir in the glovebox under room temperature, or taken out of the glovebox to be placed in a heating block if a higher reaction temperature was needed. At desired time points, a small aliquot was analyzed by ^{1}H NMR characterization to determine the conversion. The polymerization was quenched by adding $CHCl_3$, and then the diluted reaction mixture was precipitated into an excess of n-pentane and centrifugated. The precipitate was then dissolved in $CHCl_3$ (2 mL) again, precipitated into an excess of n-pentane and centrifugated for three more cycles. The precipitate was finally dried under vacuum.

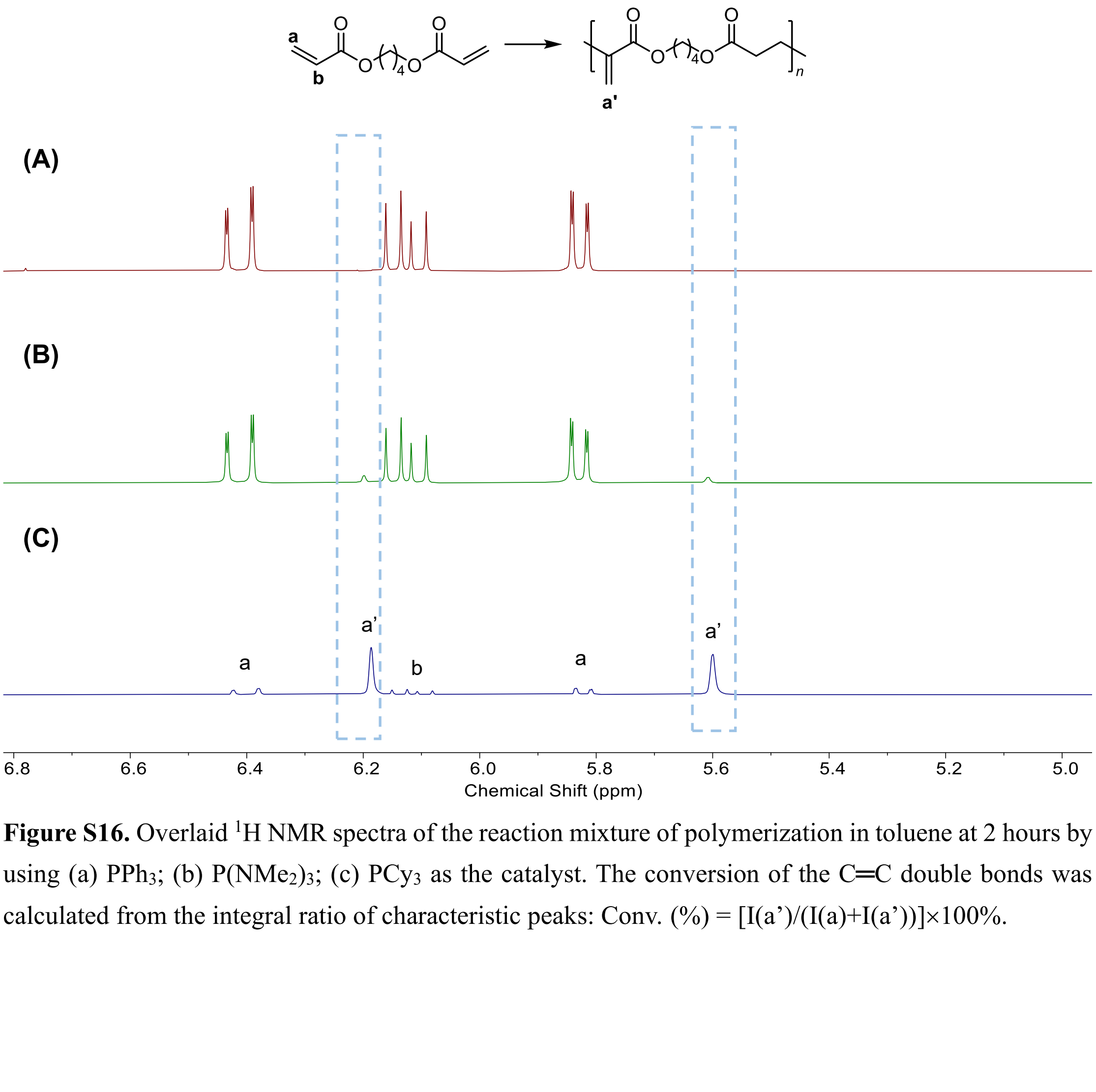

Figure S16. Overlaid ^{1}H NMR spectra of the reaction mixture of polymerization in toluene at 2 hours by using (a) PPh_3; (b) $P(NMe_2)_3$; (c) PCy_3 as the catalyst. The conversion of the C═C double bonds was calculated from the integral ratio of characteristic peaks: Conv. (%) = [I(a')/(I(a)+I(a'))]×100%.

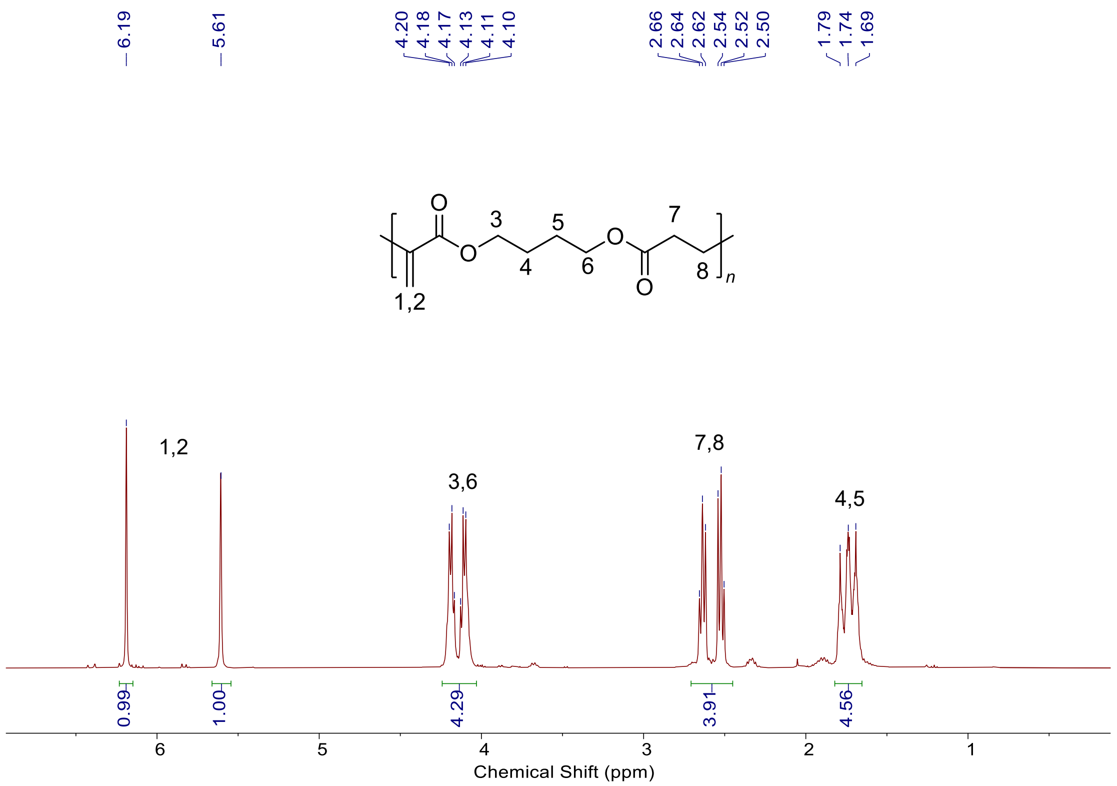

Figure S17. ^{1}H NMR (400 MHz, $CDCl_3$) spectrum of PBDA (Entry 9, Table 1).

^{1}H NMR (400 MHz, $CDCl_3$), δ (ppm): 6.19 (1H, s), 5.61 (1H, s), 4.15 (4H, m), 2.58 (4H, dt, ^{2}J = 7.2 Hz, ^{3}J = 45.8 Hz), 1.74 (4H, m).

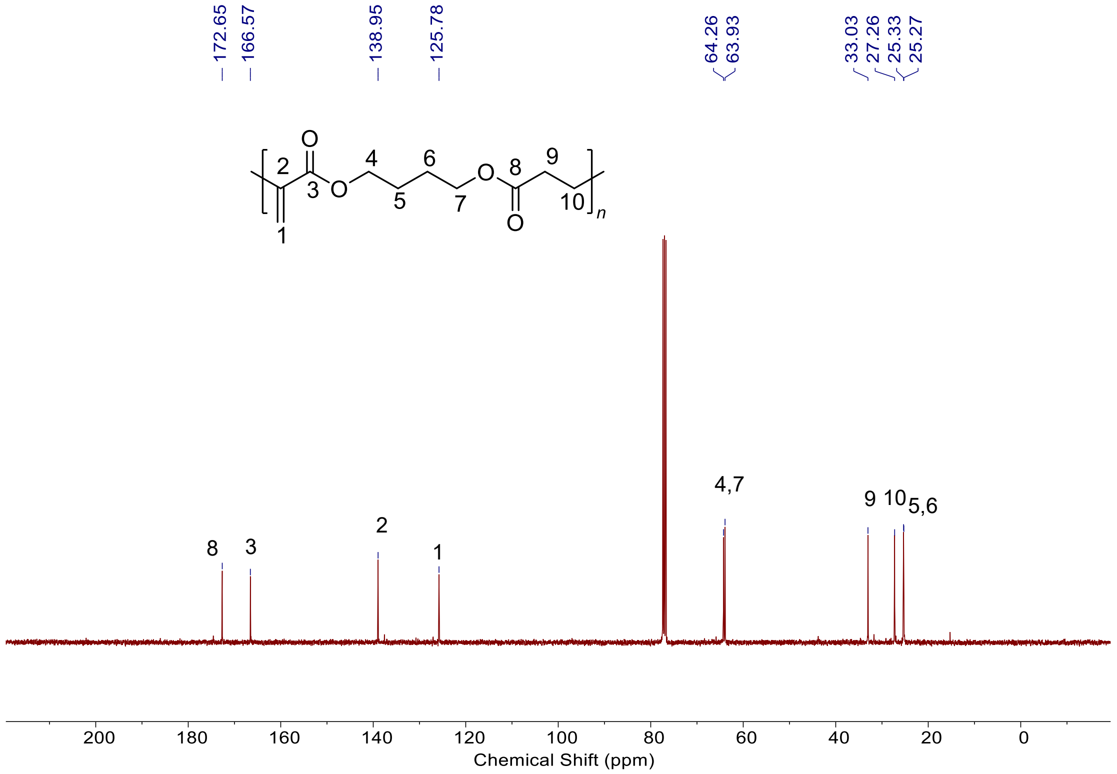

Figure S18. ^{13}C NMR (101 MHz, $CDCl_3$) spectrum of PBDA (Entry 9, Table 1).

^{13}C NMR (101 MHz, $CDCl_3$), δ (ppm): 172.65, 166.57, 138.95, 125.78, 64.26, 63.93, 33.03, 27.26, 25.33, 25.27.

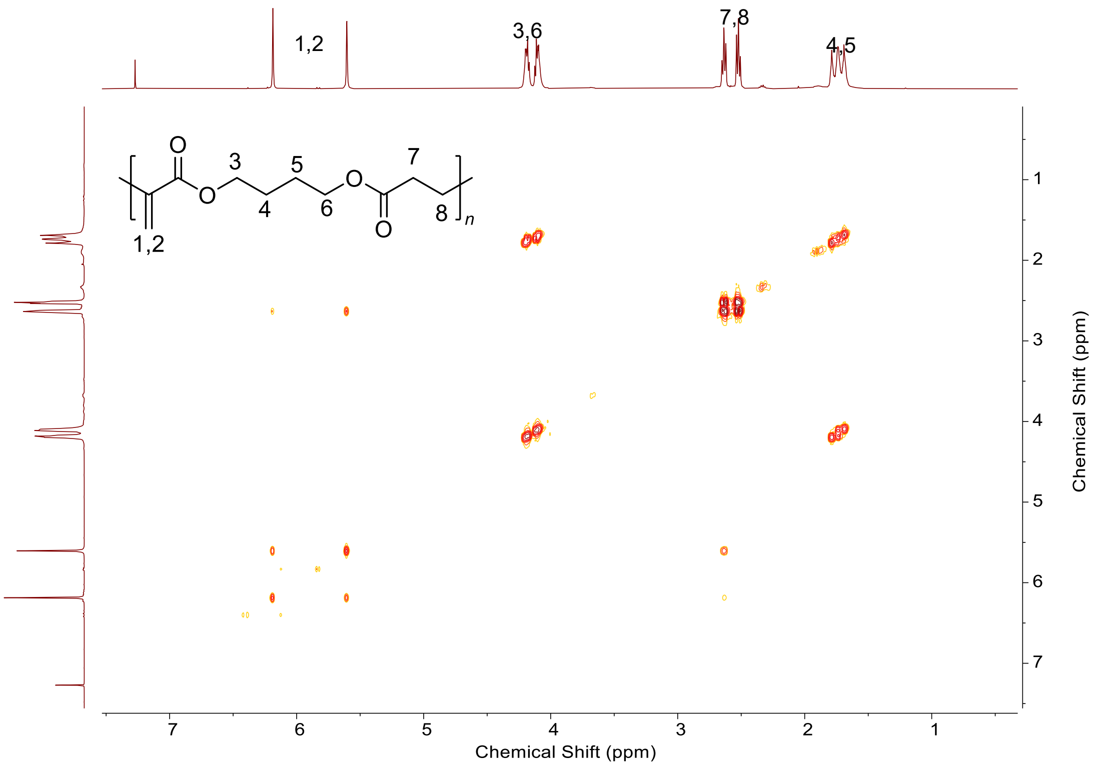

Figure S19. ^{1}H-^{1}H COSY NMR ($CDCl_3$) spectrum of PBDA (Entry 9, Table 1).

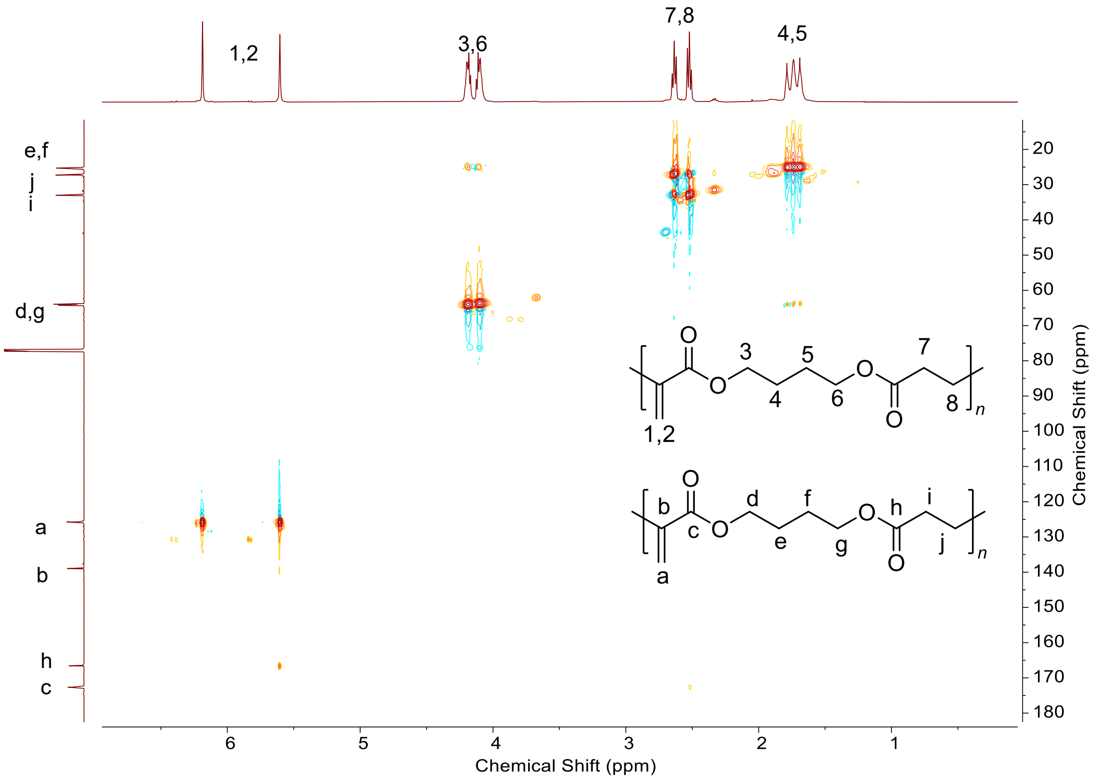

Figure S20. ^{1}H-^{13}C HSQC NMR ($CDCl_3$) spectrum of PBDA (Entry 9, Table 1).

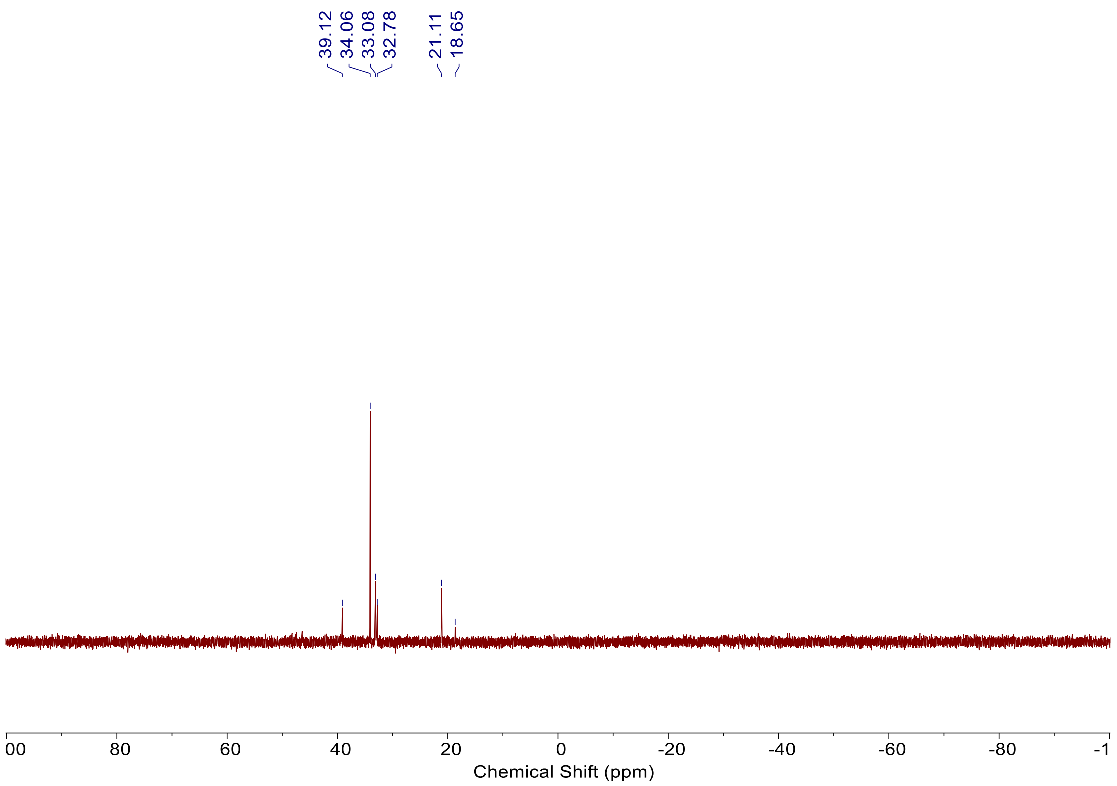

Figure S21. ^{31}P NMR (162 MHz, $CDCl_3$) spectrum of PBDA (Entry 9, Table 1).

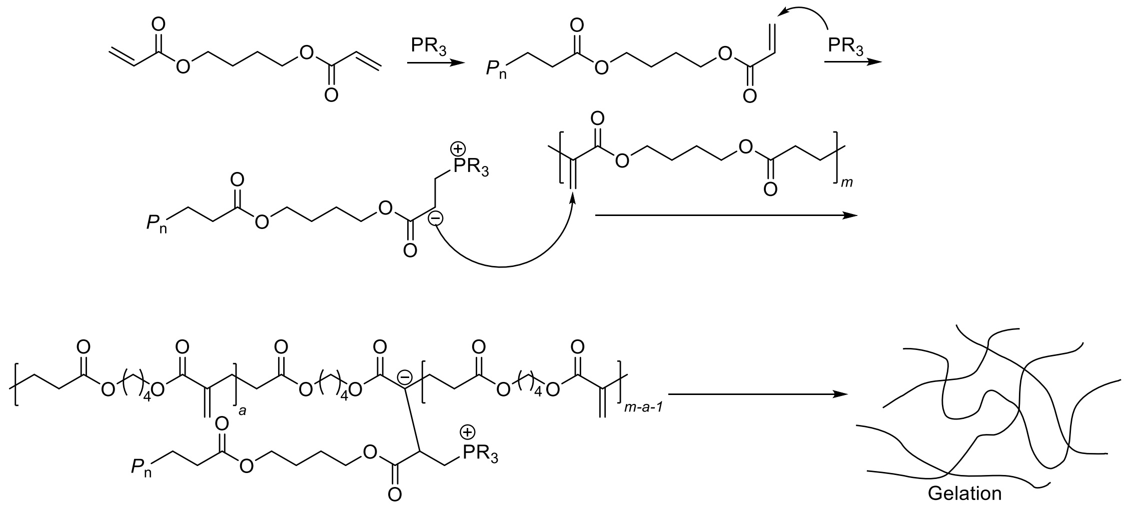

Figure S22. Possible mechanism of gelation by the formation of insoluble crosslinking network when the conversion of BDA approached slightly below 100%.

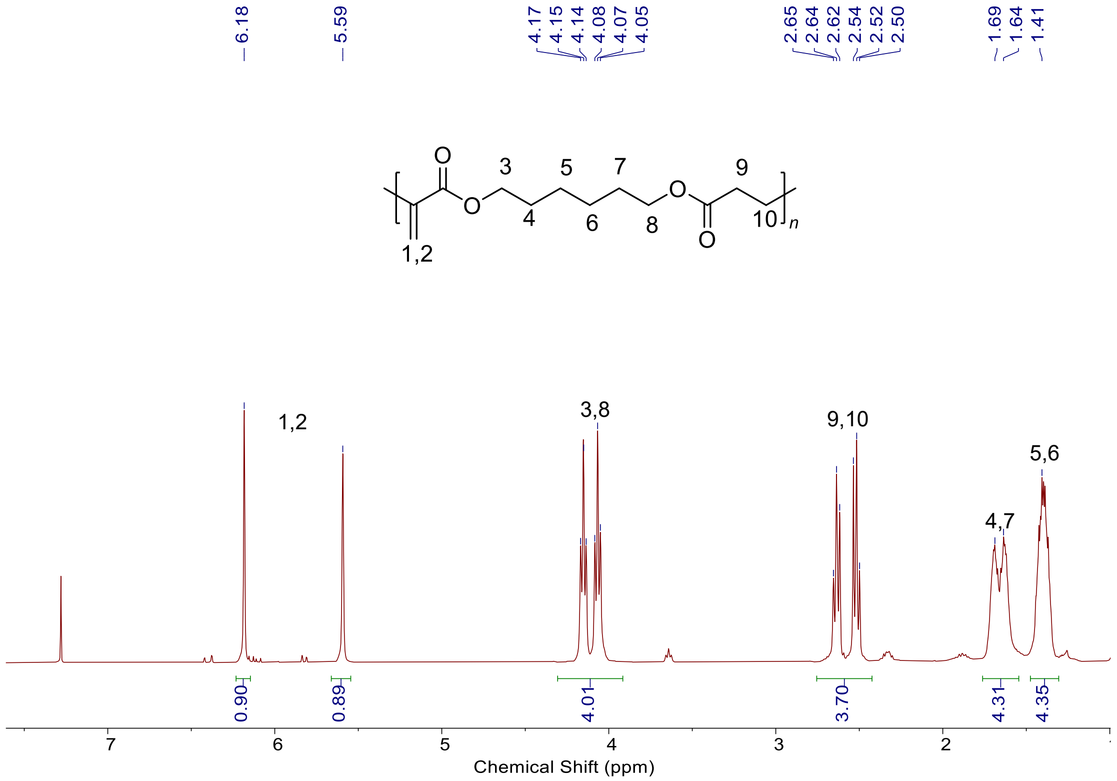

Figure S23. ^{1}H NMR (400 MHz, $CDCl_3$) spectrum of PHDA (Entry 2, Table 2).

^{1}H NMR (400 MHz, $CDCl_3$), δ (ppm): 6.18 (1H, s), 5.59 (1H, s), 4.11 (4H, dt, ^{2}J = 7.0 Hz, ^{3}J = 34.0 Hz), 2.58 (4H, dt, ^{2}J = 7.2 Hz, ^{3}J = 47.7 Hz), 1.66 (4H, m), 1.41(4H, m).

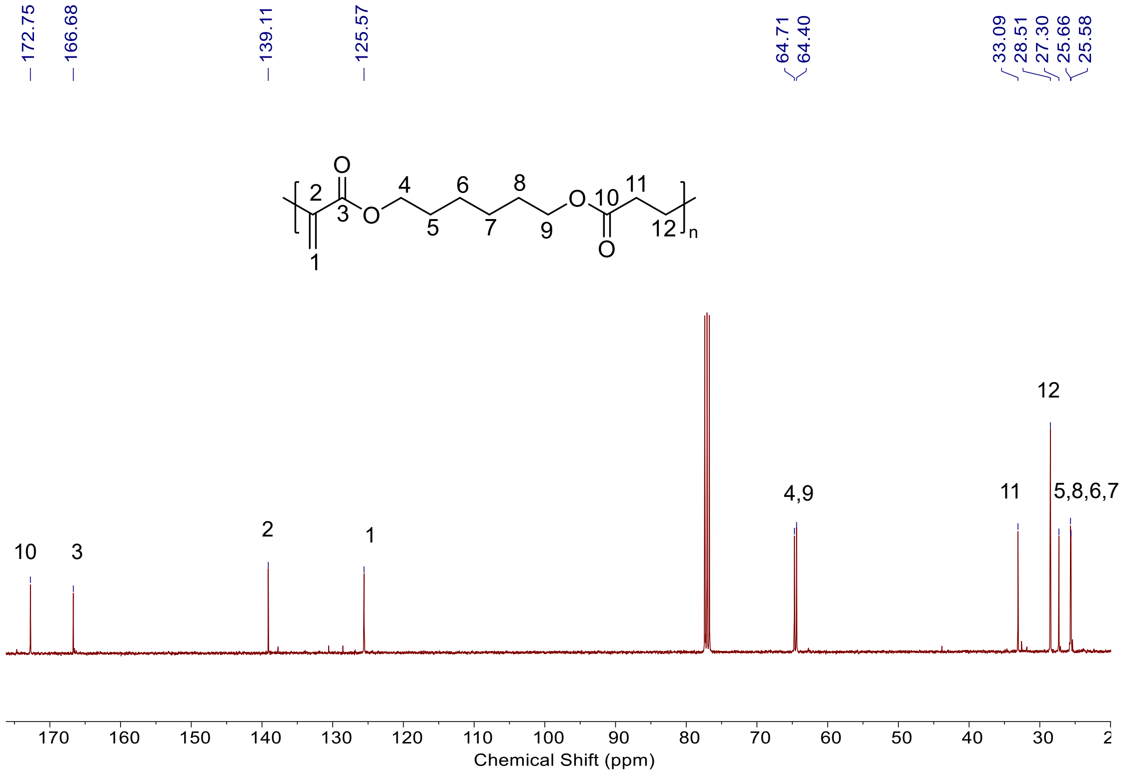

Figure S24. ^{13}C NMR (101 MHz, $CDCl_3$) spectrum of PHDA (Entry 2, Table 2).

^{13}C NMR (101 MHz, $CDCl_3$), δ (ppm): 172.75, 166.68, 139.11, 125.57, 64.71, 64.40, 33.09, 28.51, 27.30, 25.66, 25.58.

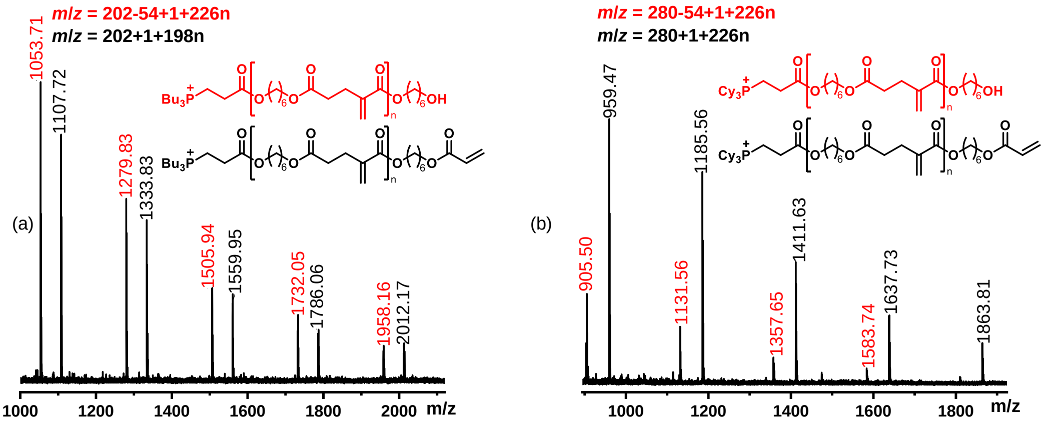

Figure S25. (a) MALDI-TOF mass spectrum of PBu_3-catalyzed HDA (Entry 1, Table 2). (b) MALDI-TOF mass spectrum of PCy_3-catalyzed HDA (Entry 2, Table 2).

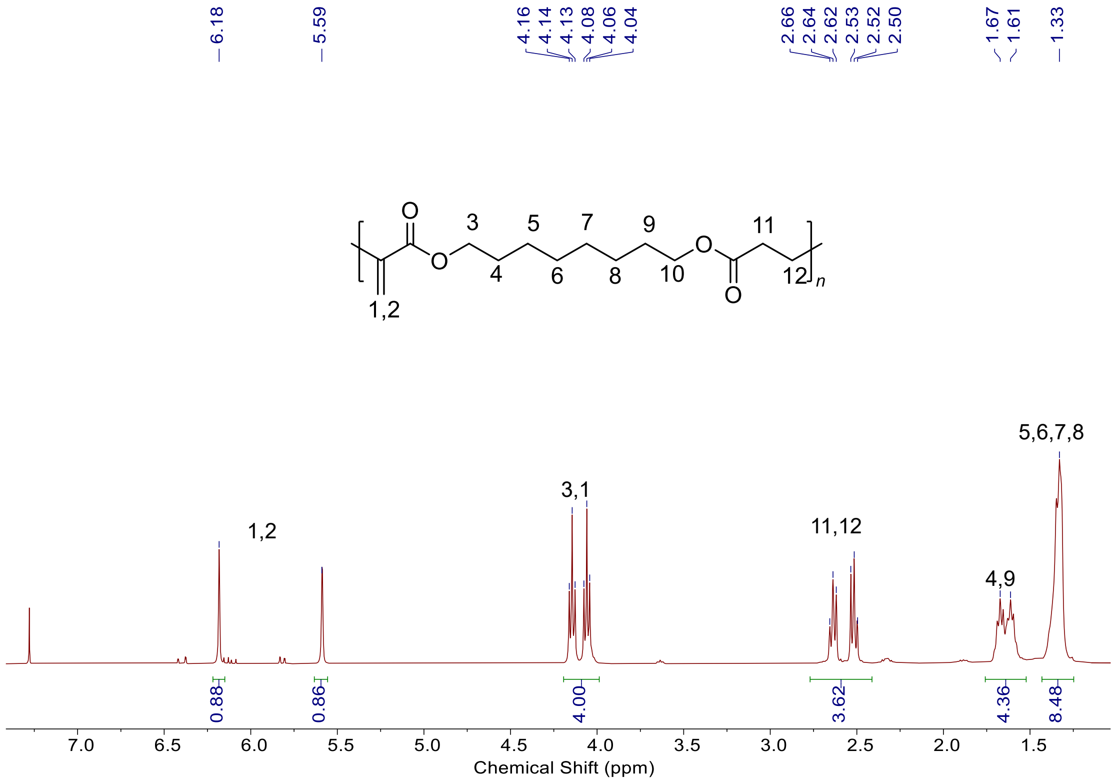

Figure S26. ^1H NMR (400 MHz, $CDCl_3$) spectrum of PODA (Entry 4, Table 2).

^1H NMR (400 MHz, $CDCl_3$), δ (ppm): 6.18 (1H, s), 5.59 (1H, s), 4.10 (4H, dt, $^2J = 6.7$ Hz, $^3J = 34.8$ Hz), 2.59 (4H, m), 1.64 (4H, m), 1.33(8H, m).

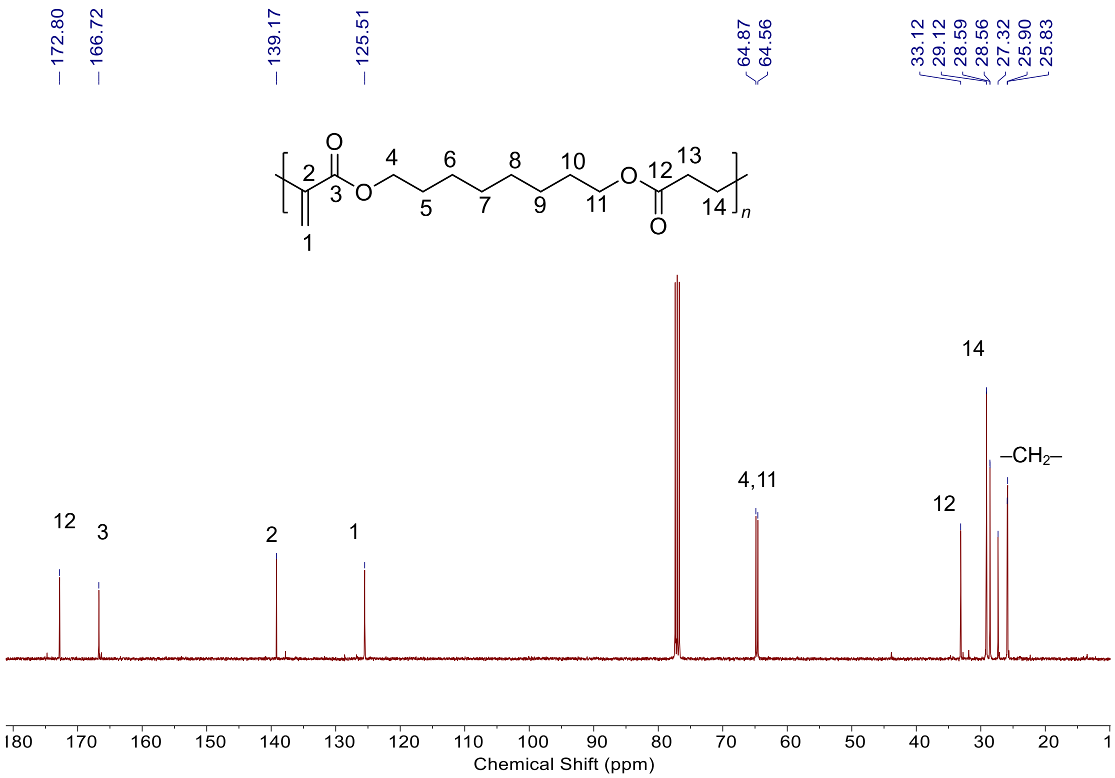

Figure S27. ^{13}C NMR (101 MHz, $CDCl_3$) spectrum of PODA (Entry 4, Table 2).

^{13}C NMR (101 MHz, $CDCl_3$), δ (ppm): 172.80, 166.72, 139.17, 125.51, 64.87, 64.56, 33.12, 29.12, 28.59, 28.26, 27.32, 25.90, 25.83.

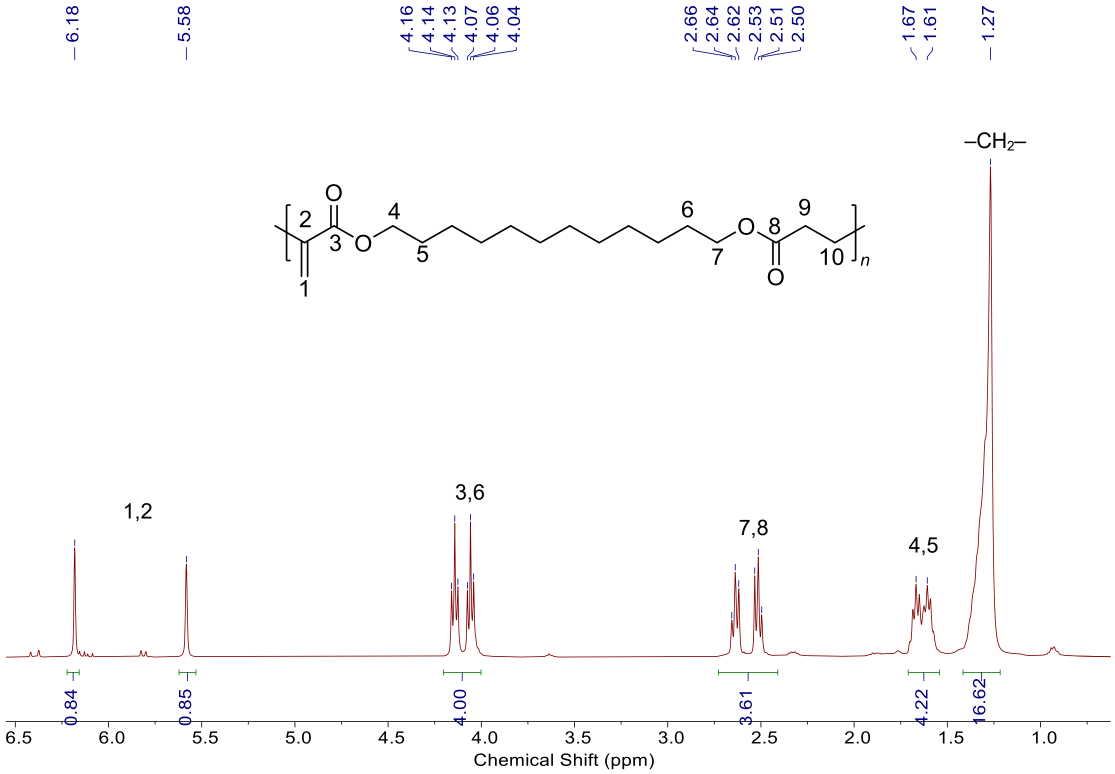

Figure S28. ^{1}H NMR (400 MHz, $CDCl_3$) spectrum of PDoDA (Entry 6, Table 2).

^{1}H NMR (400 MHz, $CDCl_3$), δ (ppm): 6.18 (1H, s), 5.58 (1H, s), 4.10 (4H, dt, ^{2}J = 6.7 Hz, ^{3}J = 33.9 Hz), 2.58 (4H, dt, ^{2}J = 7.4 Hz, ^{3}J = 49.3 Hz), 1.64 (4H, m), 1.27(16H, m).

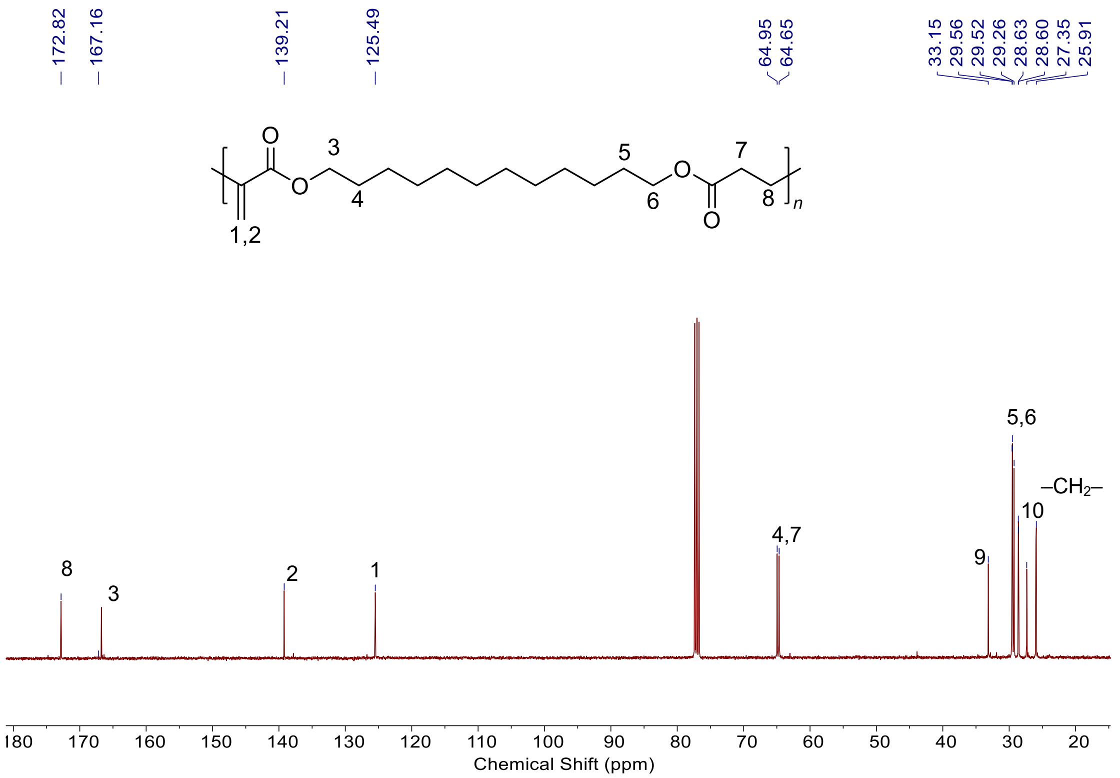

Figure S29. ^{13}C NMR (101 MHz, $CDCl_3$) spectrum of PDoDA (Entry 6, Table 2).

^{13}C NMR (101 MHz, $CDCl_3$), δ (ppm): 172.82, 167.16, 139.21, 125.49, 64.95, 64.65, 33.15, 29.56, 29.52, 29.26, 28.63, 28.60, 27.35, 25.91.

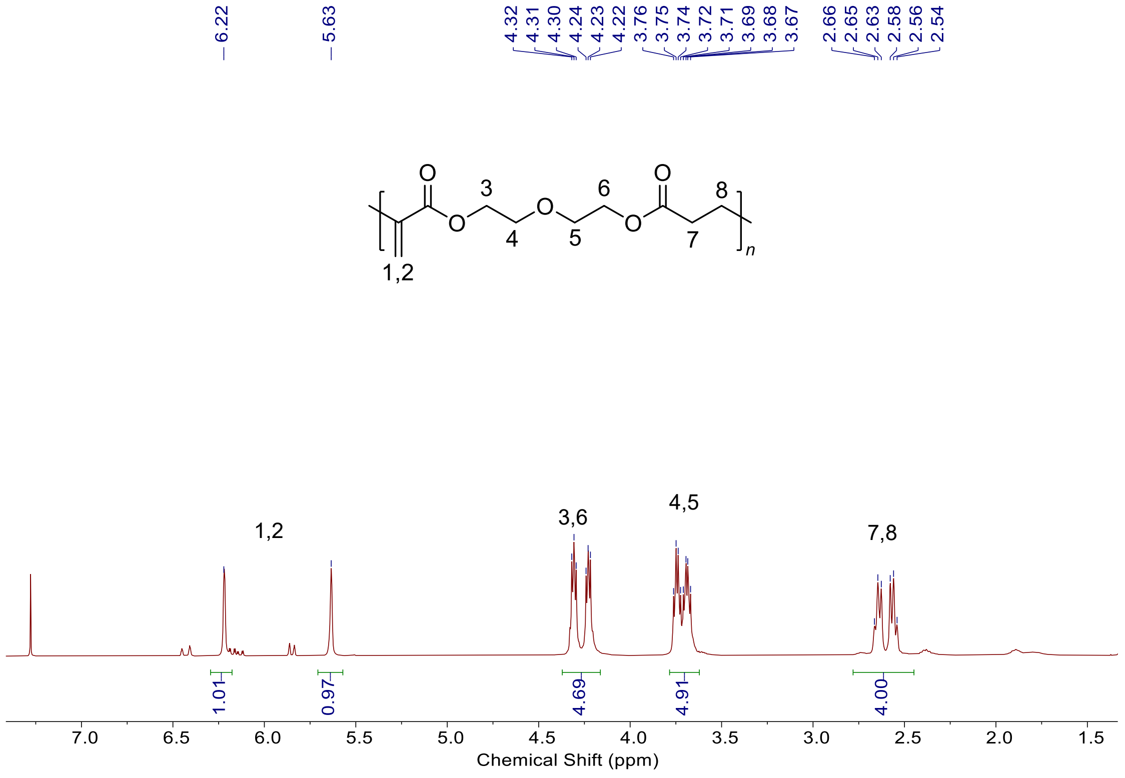

Figure S30. ^{1}H NMR (400 MHz, $CDCl_3$) spectrum of PGDA (Entry 11, Table 2).

^{1}H NMR (400 MHz, $CDCl_3$), δ (ppm): 6.22 (1H, s), 5.63 (1H, s), 4.27 (4H, dt, ^{2}J = 5.0 Hz, ^{3}J = 31.2 Hz), 3.72 (4H, ddd, ^{2}J = 7.4, 10.0, 21.9 Hz), 2.59 (4H, m).

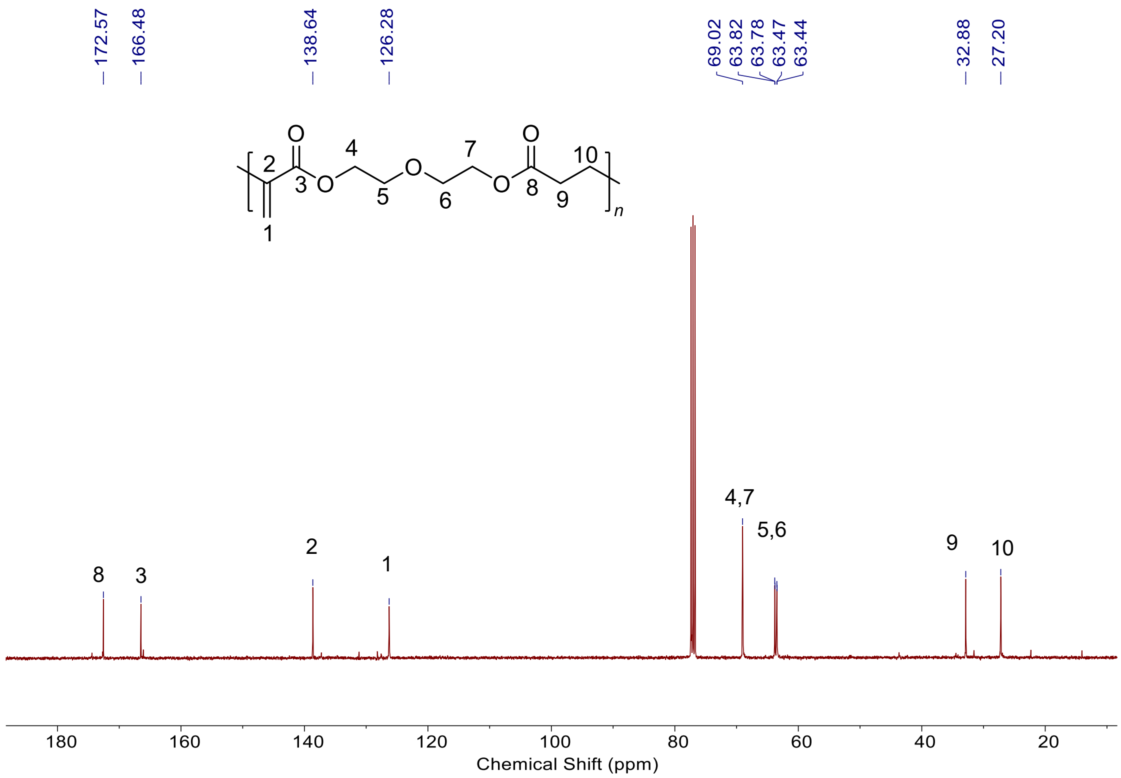

Figure S31. ^{13}C NMR (101 MHz, $CDCl_3$) spectrum of PGDA (Entry 11, Table 2).

^{13}C NMR (101 MHz, $CDCl_3$), δ (ppm): 172.57, 167.48, 139.64, 126.28, 69.02, 63.82, 63.78, 63.74, 32.88, 27.20.

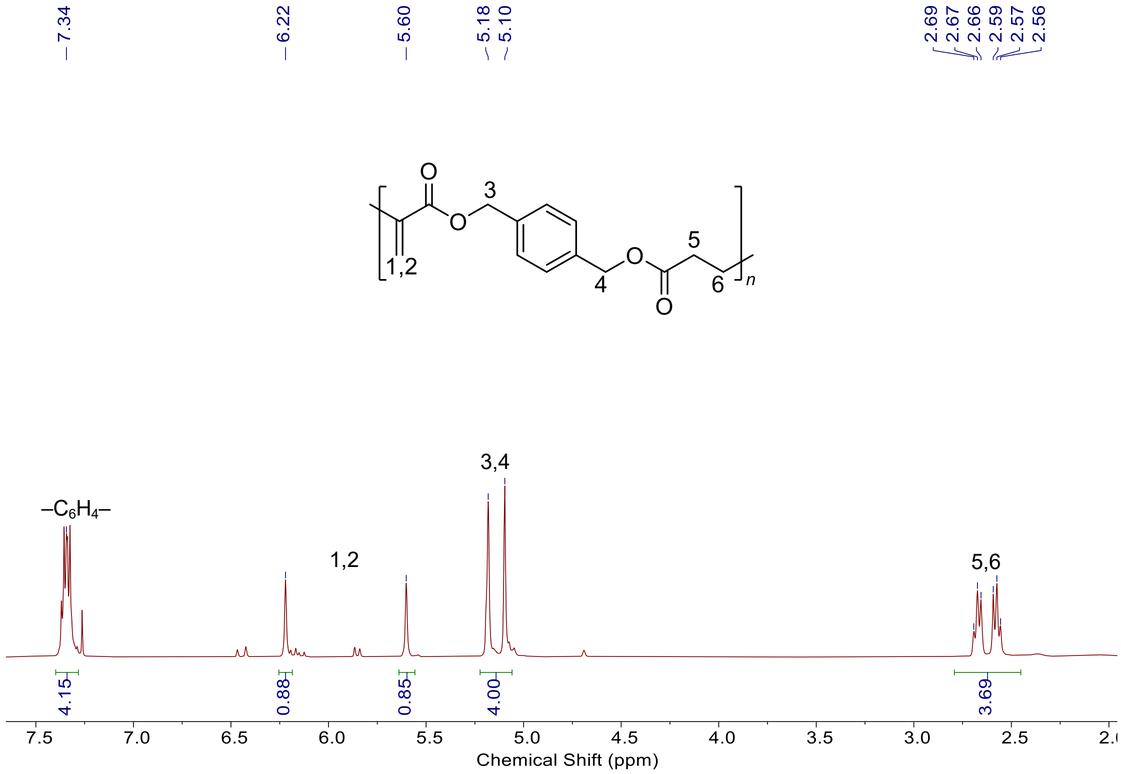

Figure S32. ^{1}H NMR (400 MHz, $CDCl_3$) spectrum of PXDA (Entry 9, Table 2).

^{1}H NMR (400 MHz, $CDCl_3$), δ (ppm): 7.34 (4H, m), 6.22 (1H, s), 5.60 (1H, s), 5.18 (2H, s), 5.10 (2H, s), 2.62 (4H, m).

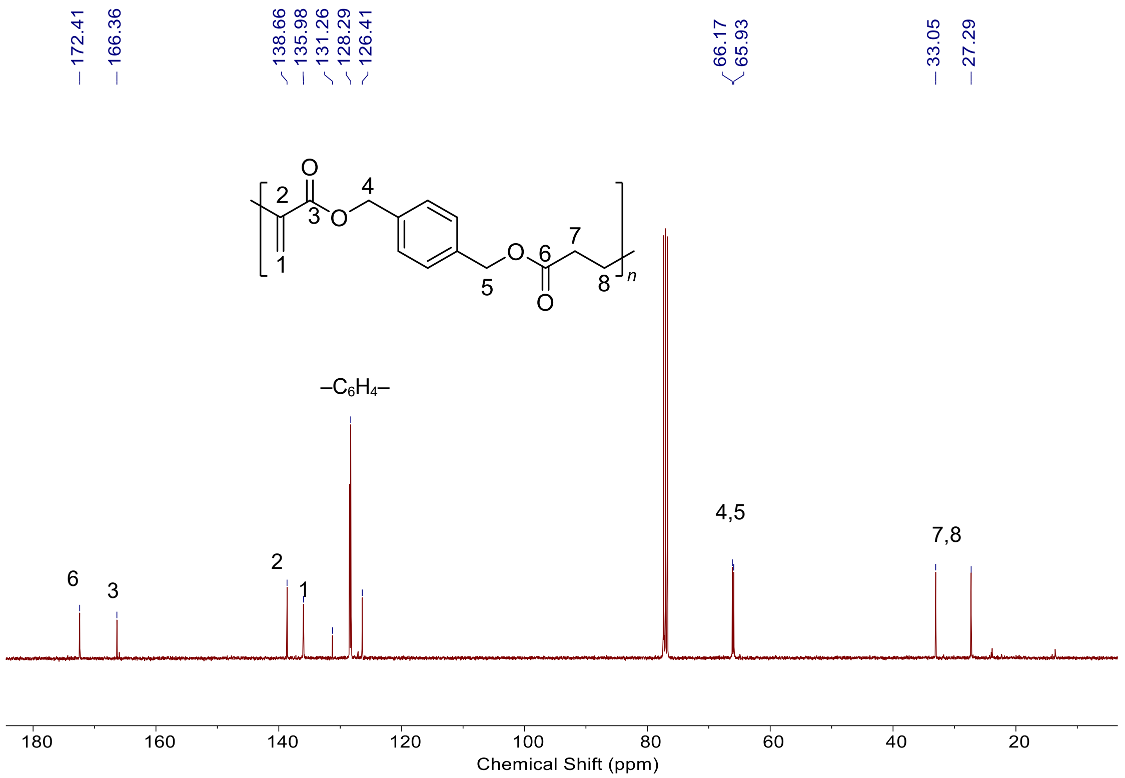

Figure S33. ^{13}C NMR (101 MHz, $CDCl_3$) spectrum of PXDA (Entry 9, Table 2).

^{13}C NMR (101 MHz, $CDCl_3$), δ (ppm): 172.41, 166.36, 138.66, 135.98, 131.26, 128.29, 126.41, 66.17, 65.93, 33.05, 27.29.

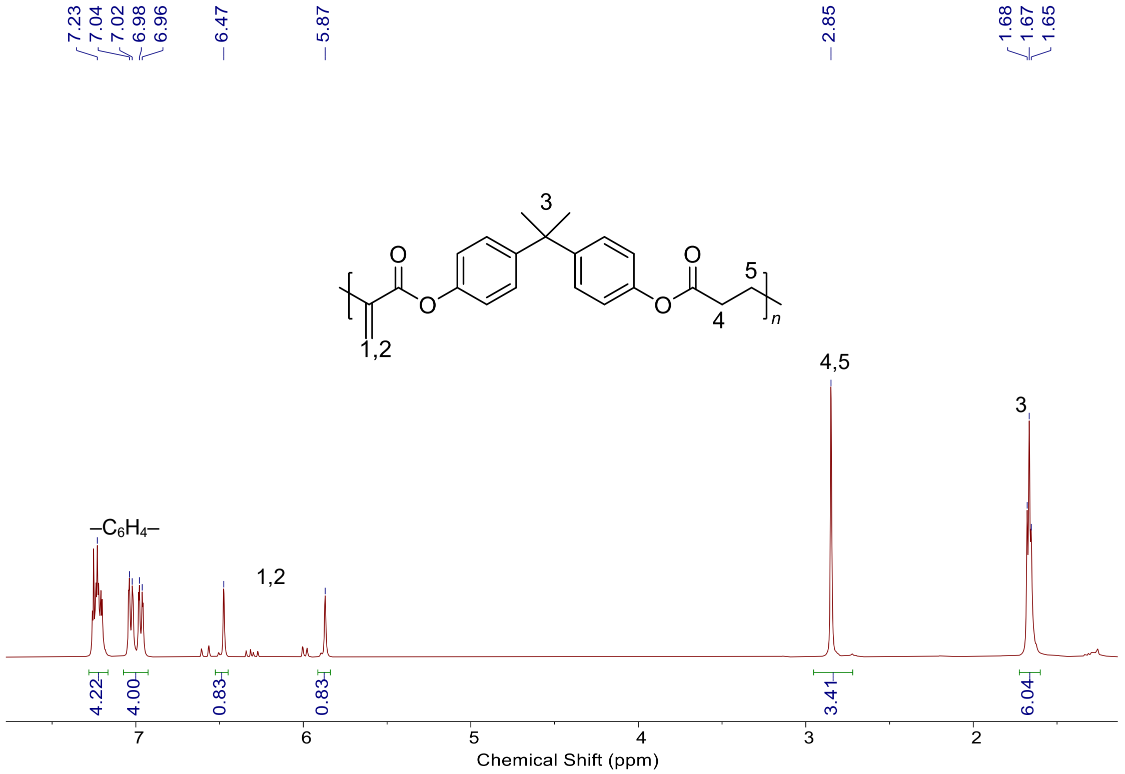

Figure S34. ^{1}H NMR (400 MHz, $CDCl_3$) spectrum of PBPDA (Entry 10, Table 2).

^{1}H NMR (400 MHz, $CDCl_3$), δ (ppm): 7.23 (4H, m), 7.00 (4H, m), 6.47 (1H, s), 5.87 (1H, s), 2.85 (4H, s), 1.67 (6H, t, J = 4.9 Hz).

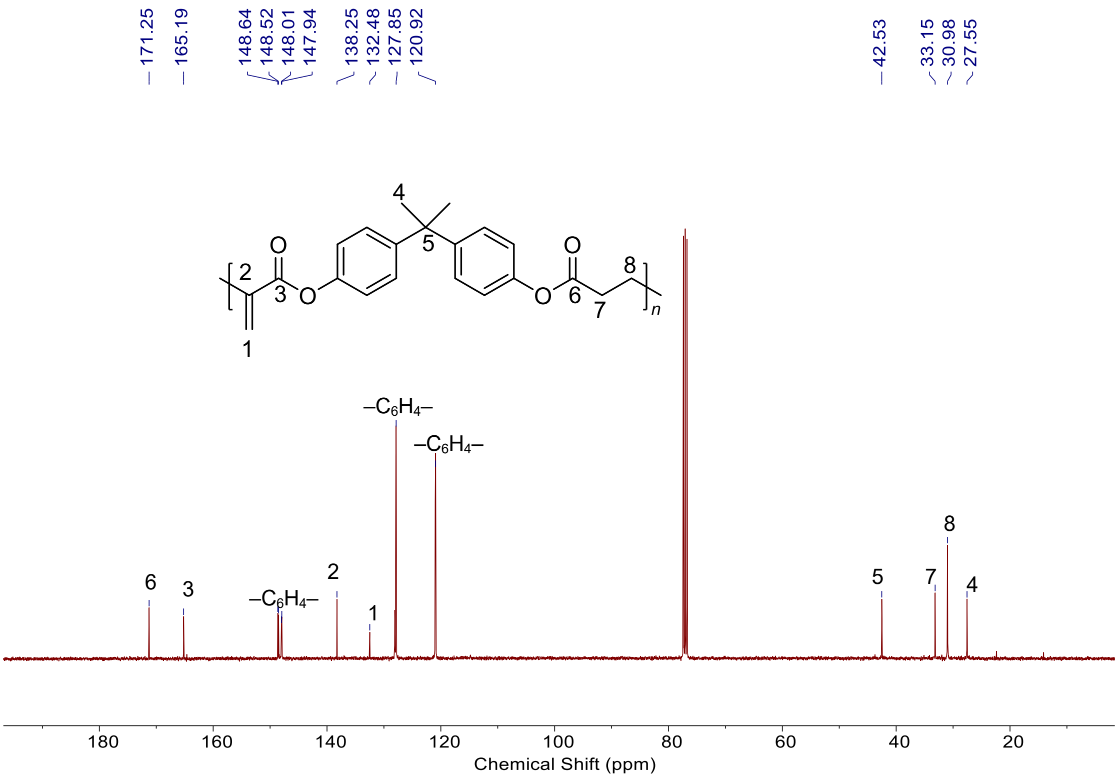

Figure S35. ^{13}C NMR (101 MHz, $CDCl_3$) spectrum of PBPDA (Entry 10, Table 2).

^{13}C NMR (101 MHz, $CDCl_3$), δ (ppm): 171.25, 165.19, 148.64, 148.52, 148.01, 147.94, 138.25, 132.48, 127.85, 120.92, 42.53, 33.15, 30.98, 27.55.

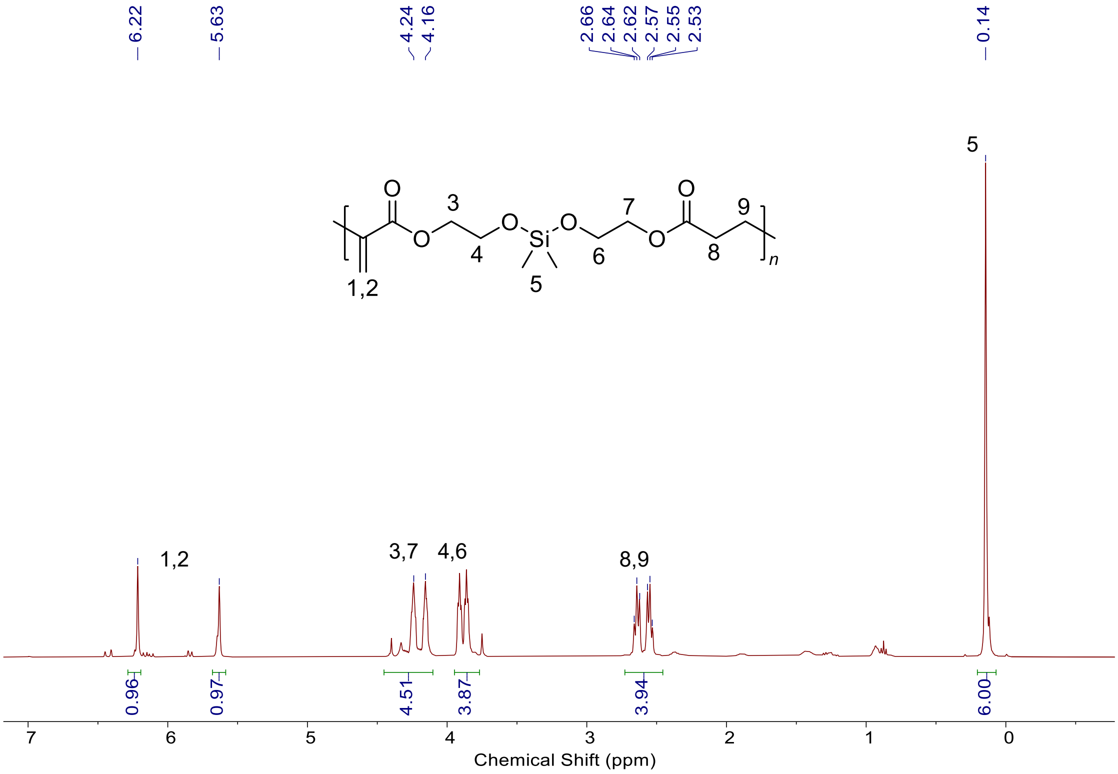

Figure S36. ^{1}H NMR (400 MHz, $CDCl_3$) spectrum of PSiPDA (Entry 8, Table 2).

^{1}H NMR (400 MHz, $CDCl_3$), δ (ppm): 6.22 (1H, s), 5.63 (1H, s), 4.20 (4H, m), 3.89 (4H, m), 2.59 (4H, dt, ^{2}J = 7.0 Hz, ^{3}J = 35.9 Hz), 0.14 (6H, s).

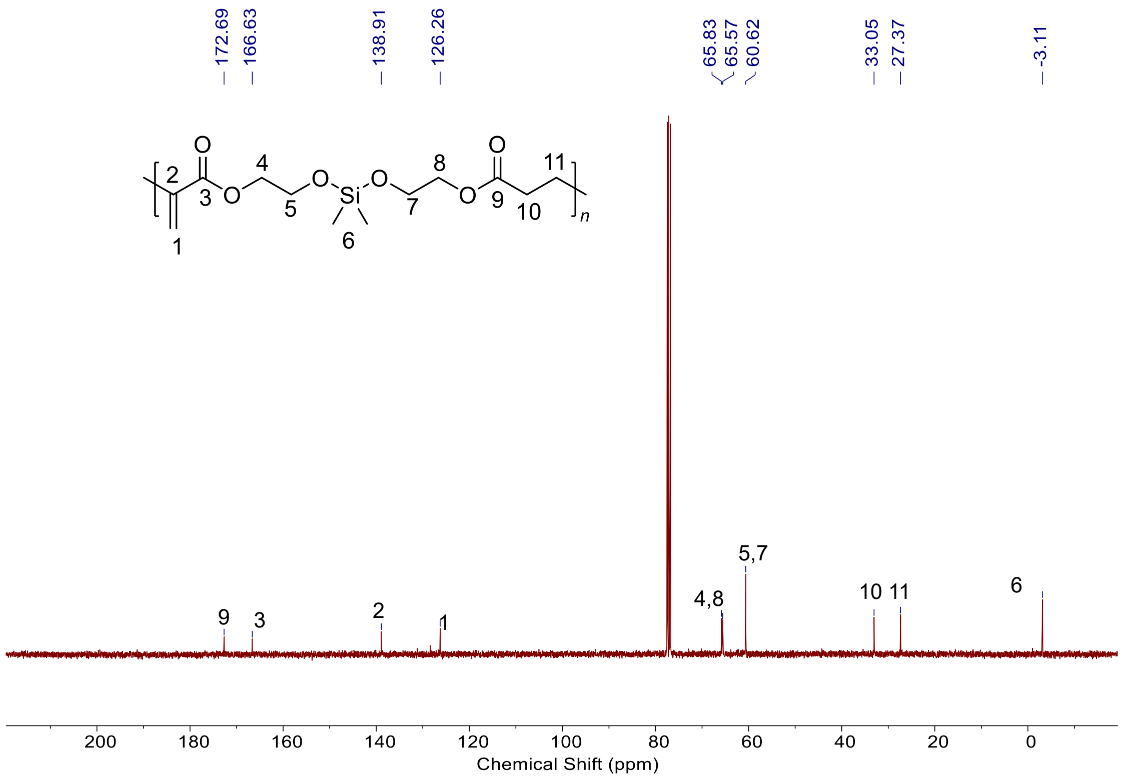

Figure S37. ^{13}C NMR (101 MHz, $CDCl_3$) spectrum of PSiDA (Entry 8, Table 2).

^{13}C NMR (101 MHz, $CDCl_3$), δ (ppm): 172.69, 166.63, 138.91, 126.26, 65.83, 65.57, 60.62, 33.05, 27.37, -3.11.

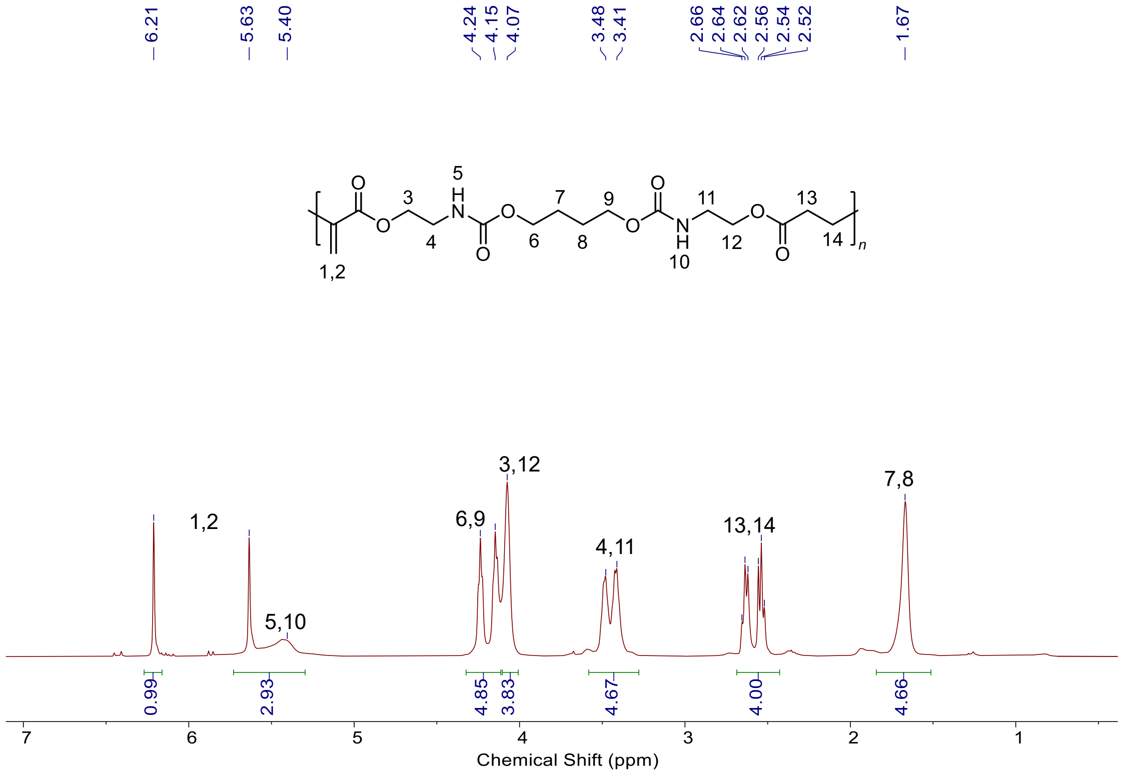

Figure S38. ^{1}H NMR (400 MHz, $CDCl_3$) spectrum of PBDDU (Entry 12, Table 2).

^{1}H NMR (400 MHz, $CDCl_3$), δ (ppm): 6.21 (1H, s), 5.63 (1H, s), 5.40 (2H, br), 4.24 (4H, m), 4.11 (4H, m), 3.45 (4H, m), 2.59 (4H, dt, ^{2}J = 7.5 Hz, ^{3}J = 39.8 Hz), 1.67 (4H, s).

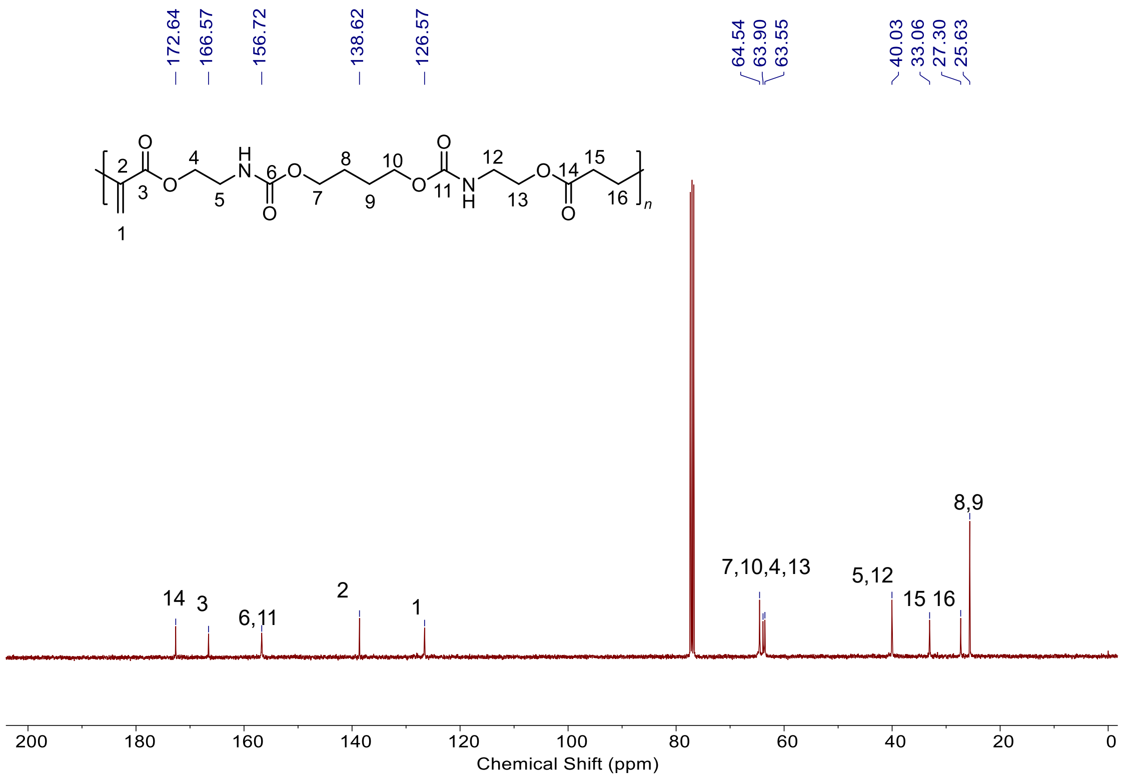

Figure S39. ^{13}C NMR (101 MHz, $CDCl_3$) spectrum of PBDDU (Entry 12, Table 2).

^{13}C NMR (101 MHz, $CDCl_3$), δ (ppm): 172.64, 166.57, 156.72, 138.62, 126.57, 64.54, 63.90, 63.55, 40.03, 33.06, 27.30, 25.63.

Calculations of the Average Molecular Weight Between Crosslinks M_c.

The gelled products were prepared under the same conditions as it was in Entry 1, Table 1 and Entries 1, 3, 5, Table 2 but without quenching. The reaction mixtures were left to gel.

The swelling experiments were conducted by immersing the samples in dichloromethane (DCM) and a minimum of three specimens were tested for each sample. The swelling degree Q is calculated from the following equation:

$$Q = \frac{m_s - m_0}{m_0} \tag{1}$$

where m_s and m_0 correspond to the weight of the swollen sample and dried sample. According to the Flory-Rehner equation, [4] the average molecular weight between crosslinks M_c can be calculated as follows:

$$\frac{1}{M_C} = \frac{2}{M_n} - \frac{\frac{V_P}{V_l}[\ln(1 - V_K) + V_K + \chi_{PK} V_K^2]}{V_K^{\frac{1}{3}} - \frac{V_K}{2}} \tag{2}$$

where V_P and V_l are corresponding to the specific volume of the polymer and molar volume of the solvent, M_n is the average molecular weight of the linear polymer before crosslinking, χ_{PK} is the Flory interaction parameter (selected as 0.37). [5] V_K represents the volume fractions of the polymer, which can be calculated as follows:

$$V_K = \frac{1}{1 + Q} \tag{3}$$

Table S1. The swelling ratio Q, the volume fractions of the polymer V_K and the average molecular weight between crosslinks M_c of gelled polymerization mixtures.

Gel	Swelling Ratio Q	Volume fractions of the polymer V_K	Average molecular weight between crosslinks M_c (kDa)	Molecular weight of monomer M_{DA}	Average number of monomer units between crosslinks
PBDA-Gel	8.77±0.30	0.102±0.003	1.65±0.01	198.22	8.3
PHDA-Gel	7.96±0.38	0.112±0.004	1.96±0.03	226.27	8.7
PODA-Gel	6.26±0.42	0.138±0.007	2.11±0.07	254.33	8.3
PDoDA-Gel	6.21±0.29	0.141±0.002	3.11±0.04	310.43	10.0

SEC Characterization of Polymers

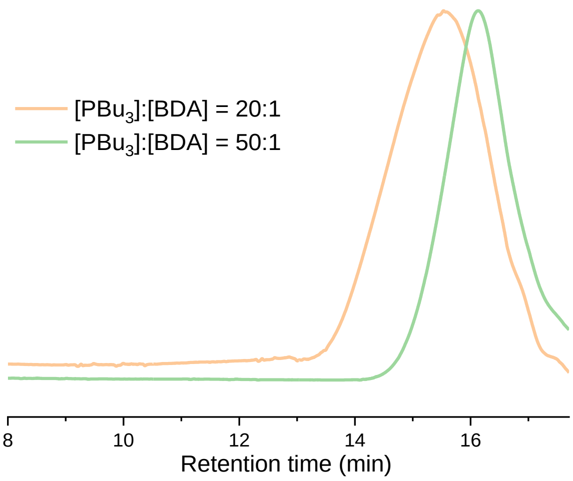

Figure S40. SEC traces (THF as the eluent) of PBDA obtained from different [monomer]:[PBu_3] ratios (Entries 1, 2, Table 1).

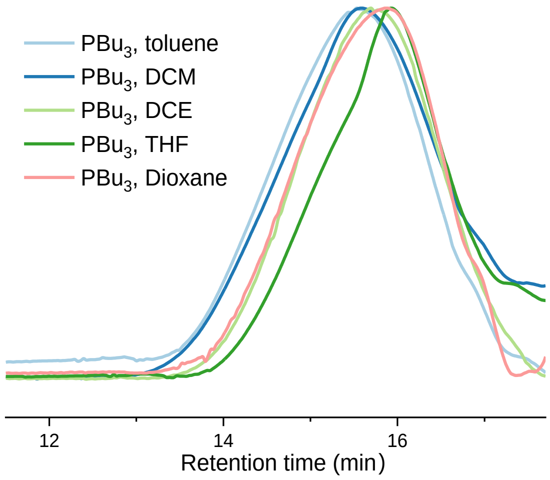

Figure S41. SEC traces (THF as the eluent) of PBDA obtained from PBu_3-catalyzed polymerization in different solvents (Entries 1&3–6, Table 1).

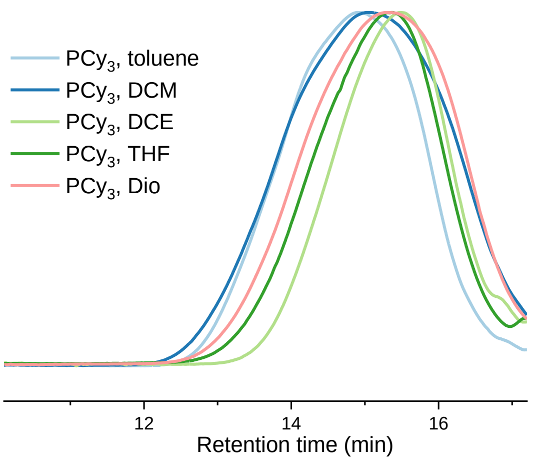

Figure S42. SEC traces (THF as the eluent) of PBDA obtained from PCy_3-catalyzed polymerization in different solvents (Entries 9–13, Table 1).

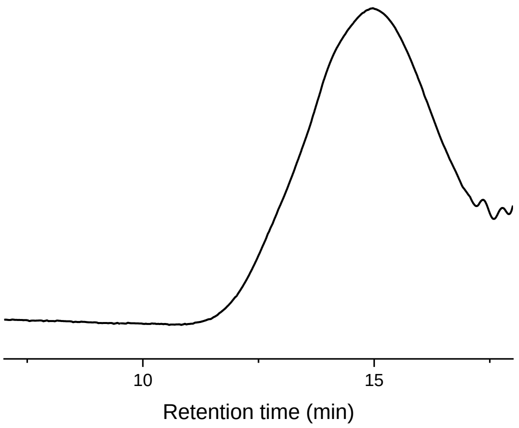

Figure S43. SEC trace (THF as the eluent) of PBDA obtained from a 20-g scale batch.

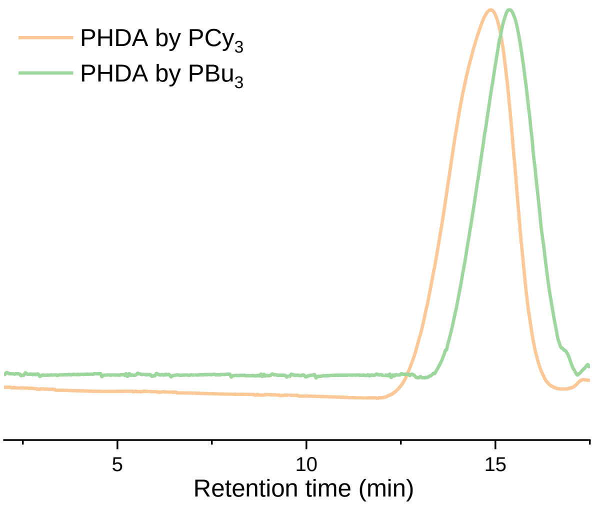

Figure S44. SEC traces (THF as the eluent) of PHDA (Entries 1, 2, Table 2).

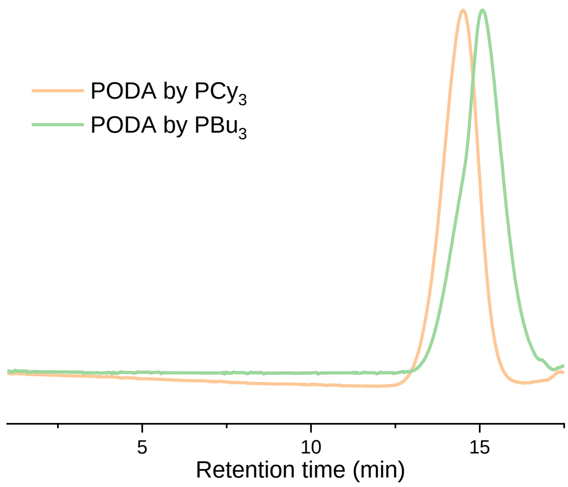

Figure S45. SEC traces (THF as the eluent) of PODA (Entries 3, 4, Table 2).

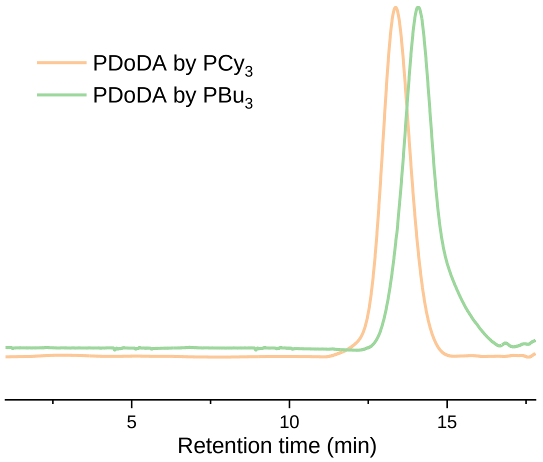

Figure S46. SEC traces (THF as the eluent) of PDoDA (Entries 5, 6, Table 2).

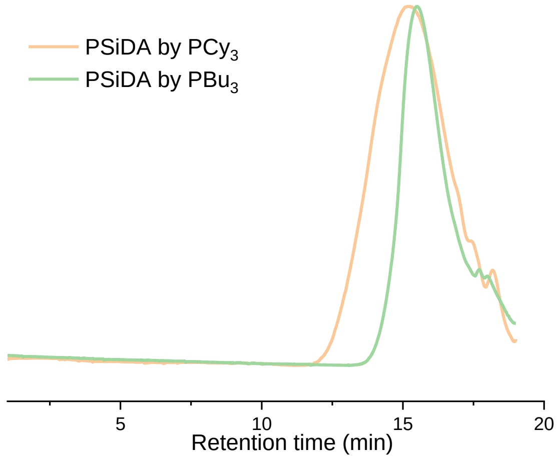

Figure S47. SEC traces (THF as the eluent) of PSiDA (Entries 7, 8, Table 2).

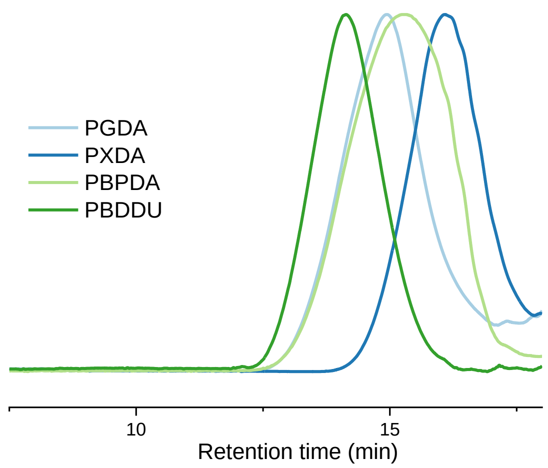

Figure S48. SEC traces (THF as the eluent) of PXDA, PBPDA, PGDA, PBDDU (Entries 9–12, Table 2).

Thermogravimetric Analysis (TGA) of Polymers

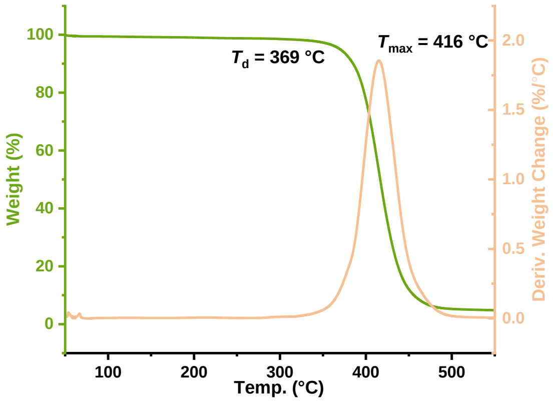

Figure S49. TGA and DTG curves of PBDA (Entry 9, Table 1).

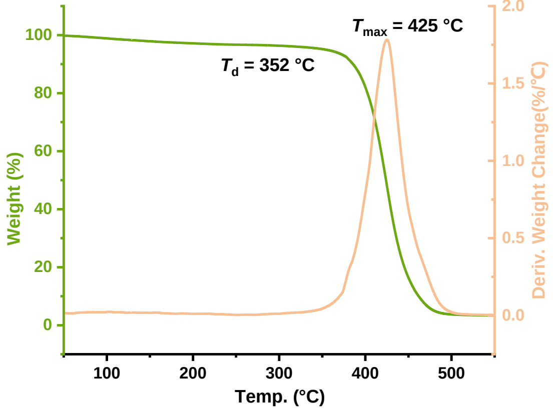

Figure S50. TGA and DTG curves of PHDA (Entry 2, Table 2).

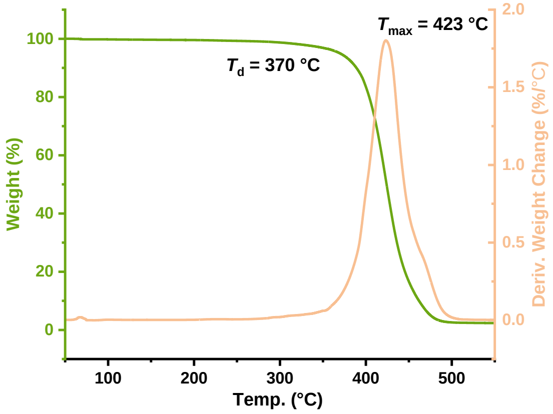

Figure S51. TGA and DTG curves of PODA (Entry 4, Table 2).

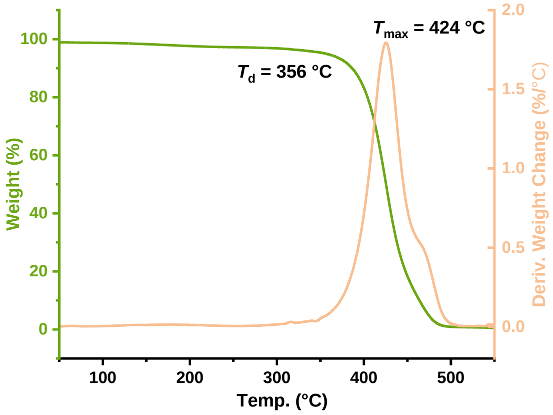

Figure S52. TGA and DTG curves of PDoDA (Entry 6, Table 2).

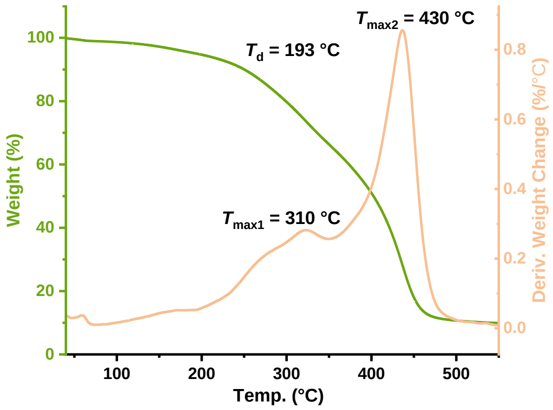

Figure S53. TGA and DTG curves of PSiDA (Entry 8, Table 2).

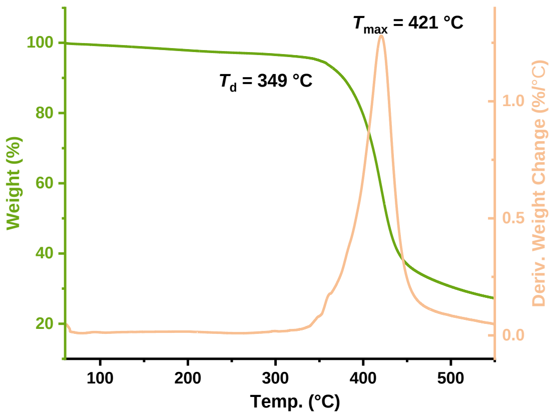

Figure S54. TGA and DTG curves of PXDA (Entry 9, Table 2).

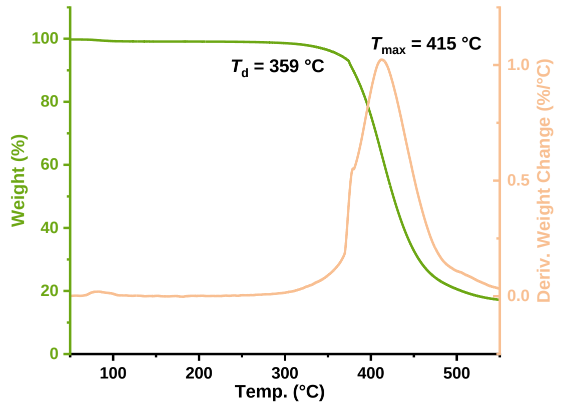

Figure S55. TGA and DTG curves of PBPDA (Entry 10, Table 2).

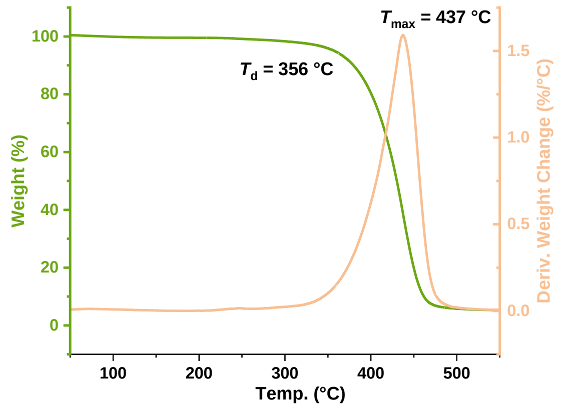

Figure S56. TGA and DTG curves of PGDA (Entry 11, Table 2).

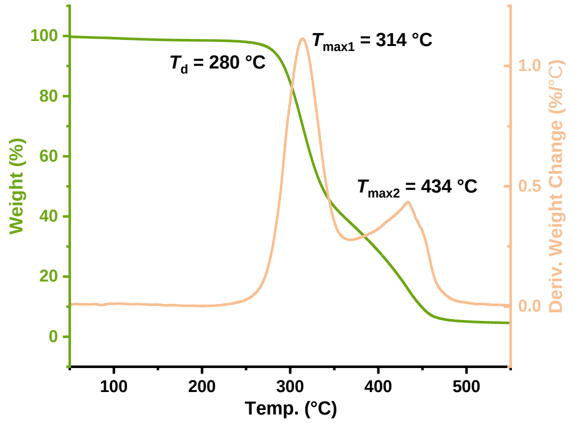

Figure S57. TGA and DTG curves of PBDDU (Entry 12, Table 2).

Post-modification of PBDA and PDoDA

One-Pot Synthesis of PBDA–ME

The polymerization condition was the same as it was stated for Entry 9, Table 1. When the conversion of BDA reached 95% in 6.5 hours, the polymerization system was diluted with toluene (500 μL), and then 2-mercaptoethanol (350 μL, 10 equivalent to BDA monomer) was added to the reaction mixture. After 4 more hours, the reaction was quenched by adding $CHCl_3$ (2 mL), and then the diluted reaction mixture was precipitated into an excess of diethyl ether and centrifugated. The precipitate was then dissolved in $CHCl_3$ (2 mL) again, precipitated into an excess of ether and centrifugated for two more cycles. The precipitate was finally dried under vacuum.

Photo-Induced Free Radical Synthesis of PBDA–ME

PBDA (obtained from Entry 9, Table 1, 49 mg, 0.25 mmol of C=C double bond) and 2-mercaptoethanol (182 μL, 2.5 mmol, 10 equivalent to the amount of C=C double bond) were dissolved in THF (300 μL) in a transparent quartz tube equipped with a magnetic stir bar in an argon-filled glovebox. 2,2-Dimethoxyl-2-phenylacetophenone (2.5 mg, 5 wt.%) was added. The tube was sealed and taken out of the glovebox and then irradiated by a UV light with a wavelength of 365 nm for 3.5 h. The reaction mixture was diluted with $CHCl_3$ (2 mL) and then precipitated into diethyl ether. The obtained precipitate was washed with diethyl ether once more and then dried under vacuum at room temperature.

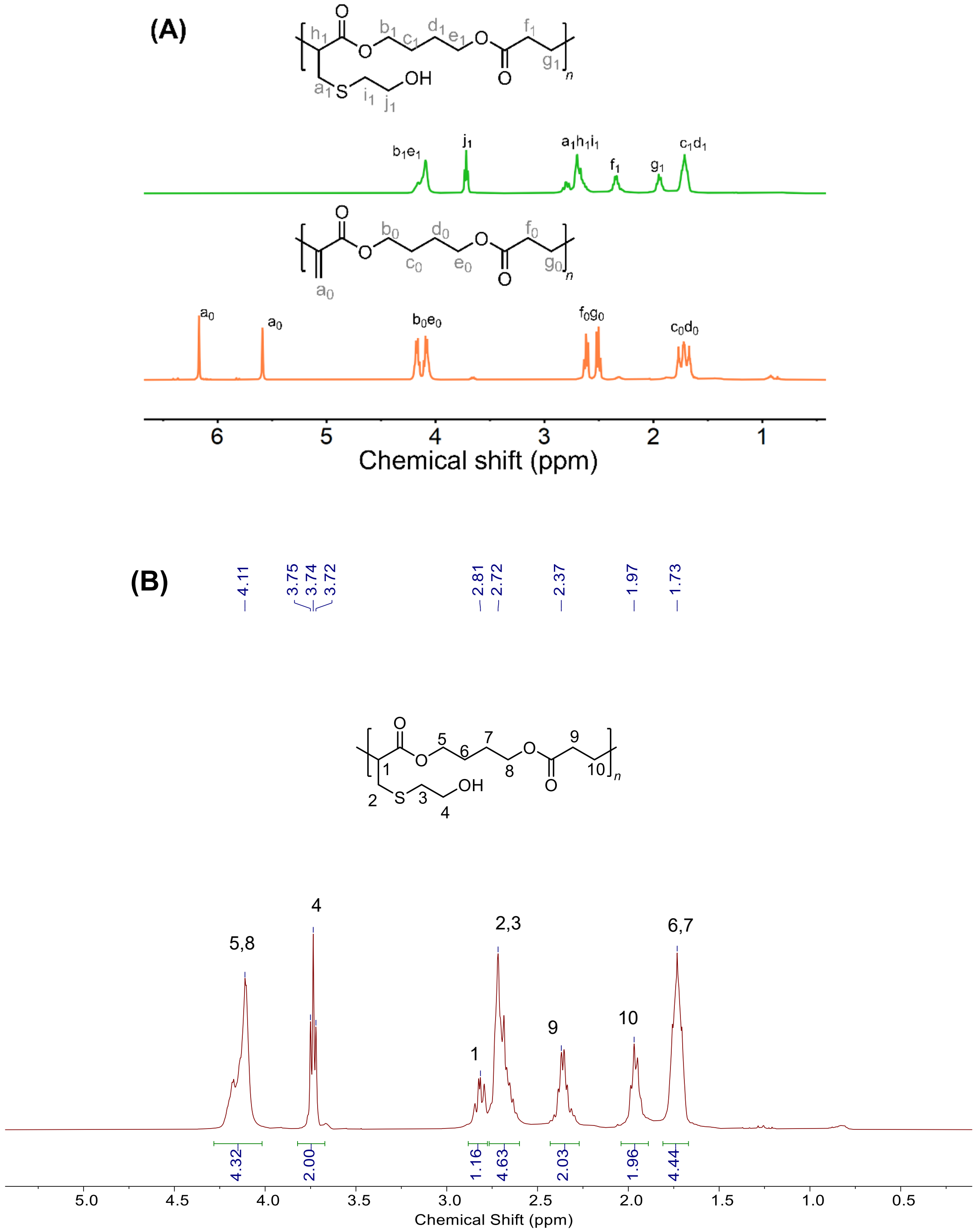

Figure S58. (A) The overlaid ^{1}H NMR (400 MHz, $CDCl_3$) spectra of PBDA and PBDA–ME. **(B)** ^{1}H NMR (400 MHz, $CDCl_3$) spectrum of PBDA–ME.

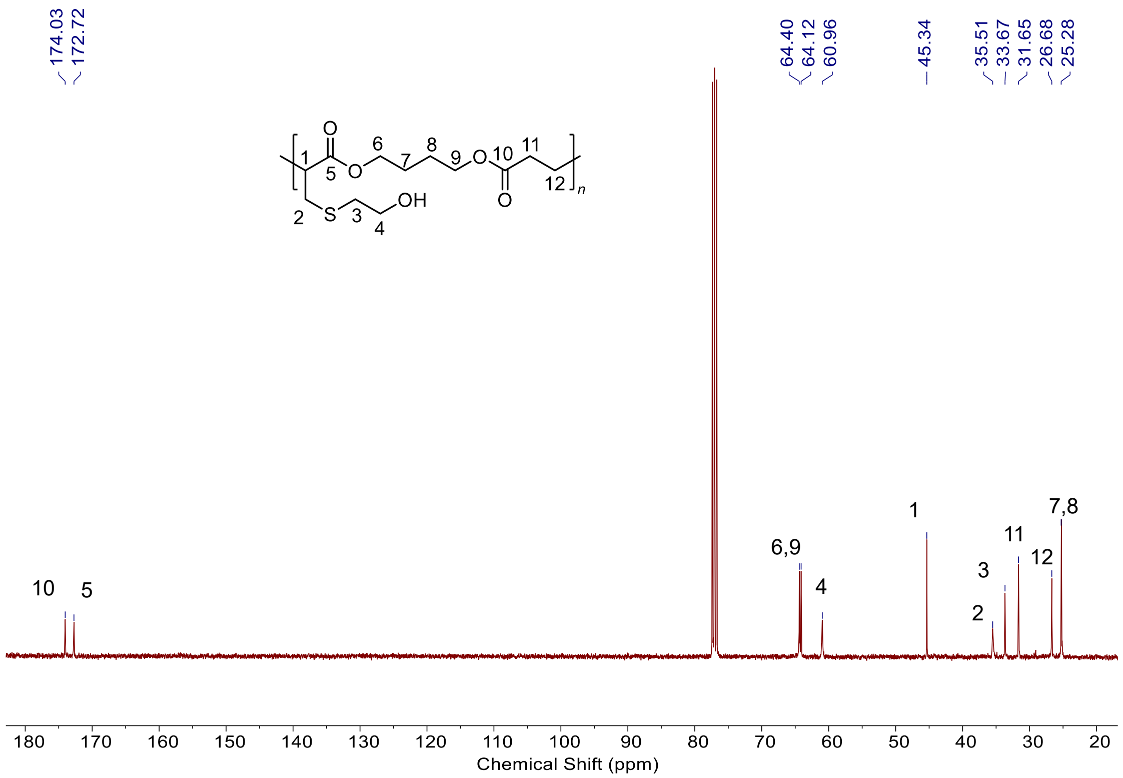

Figure S59. ^{13}C NMR (101 MHz, $CDCl_3$) spectrum of PBDA–ME.

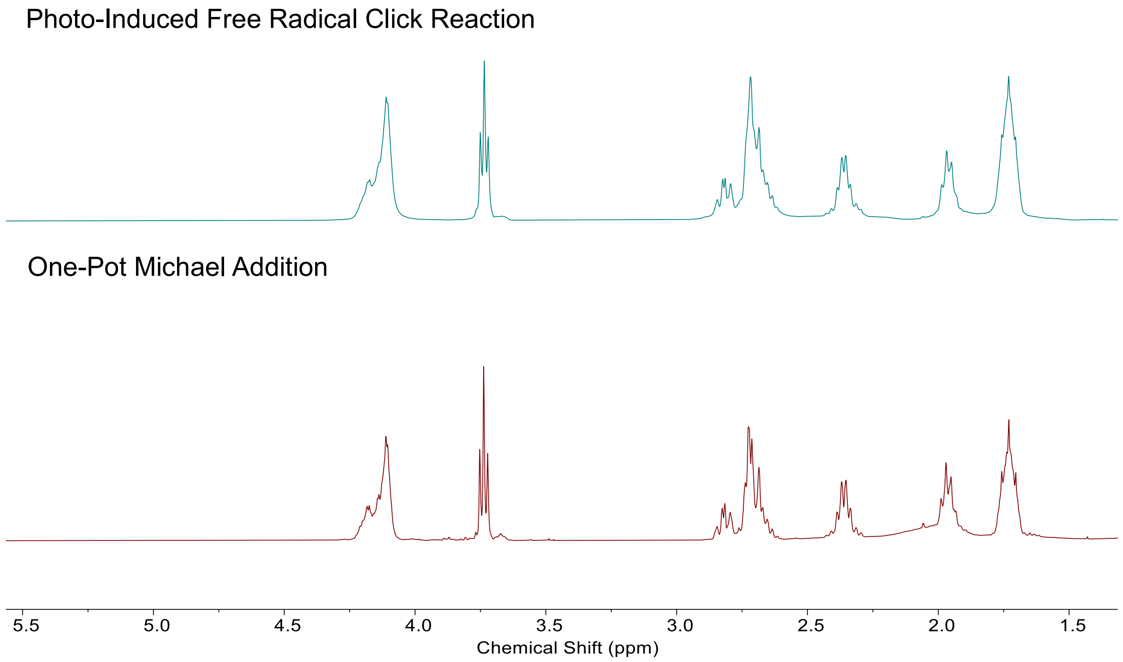

Figure S60. Overlaid ^{1}H NMR spectra (400 MHz, $CDCl_3$) of PBDA–ME acquired from one-pot thiol-Michael addition click reaction and photo-induced free radical click reaction.

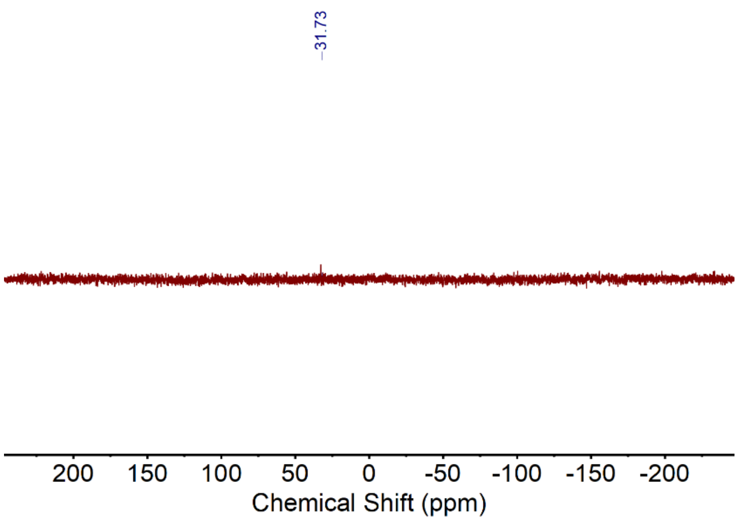

Figure S61. ^{31}P NMR ($CDCl_3$) of PBDA after quenching with HCl/methanol (0.1 M).

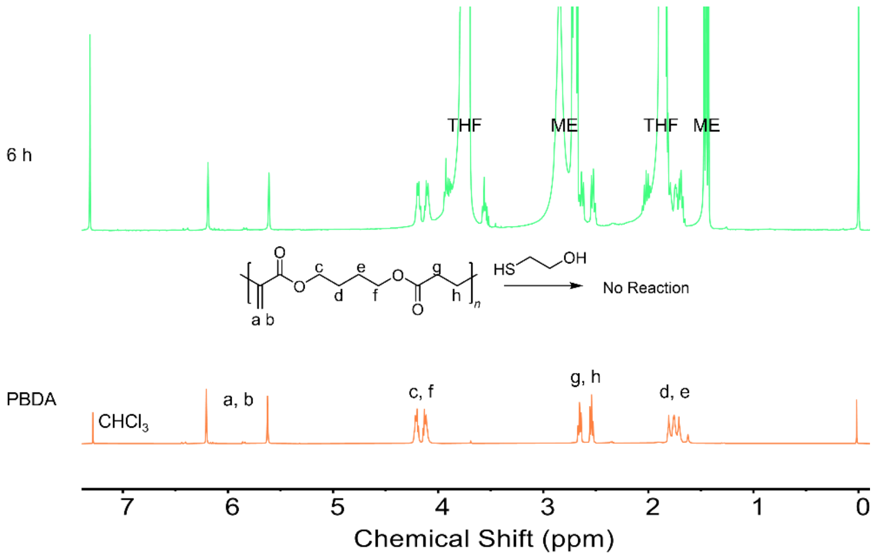

Figure S62. Overlaid ^{1}H NMR ($CDCl_3$) spectra of PBDA after removal of the phosphine (bottom) and its reaction mixture with ME after 6 h (top).

One-Pot Synthesis of PBDA–BnSH

The polymerization condition was the same as it was stated for Entry 9, Table 1. When the conversion of BDA reached 95% in 6.5 hours, benzyl mercaptan (585 μL, 10 equivalent to BDA monomer) was added to the polymerization system. After 48 more hours, the polymerization was quenched by adding $CHCl_3$ (2 mL), and then the diluted reaction mixture was precipitated into an excess of diethyl ether and centrifugated. The precipitate was then dissolved in $CHCl_3$ (2 mL) again, precipitated into an excess of ether and centrifugated for two more cycles. The precipitate was finally dried under vacuum.

Photo-Induced Free Radical Synthesis of PBDA–BnSH

PBDA (obtained from Entry 9, Table 1, 99 mg, 0.5 mmol of C=C double bond) and benzyl mercaptan (585 μL, 5 mmol, 10 equivalent to the amount of C=C double bond) were dissolved in THF (1 mL) in a transparent quartz tube with a magnetic stir bar in an argon-filled glovebox. 2,2-Dimethoxyl-2-phenylacetophenone (5 mg, 5 wt.%) was added. The tube was sealed and taken out of the glovebox. The tube was then irradiated by a UV light with a wavelength of 365 nm for 3.5 h. The reaction mixture was diluted with $CHCl_3$ (2 mL) and then precipitated into an excess of diethyl ether. The obtained precipitate was washed with diethyl ether once more and then dried under vacuum at room temperature.

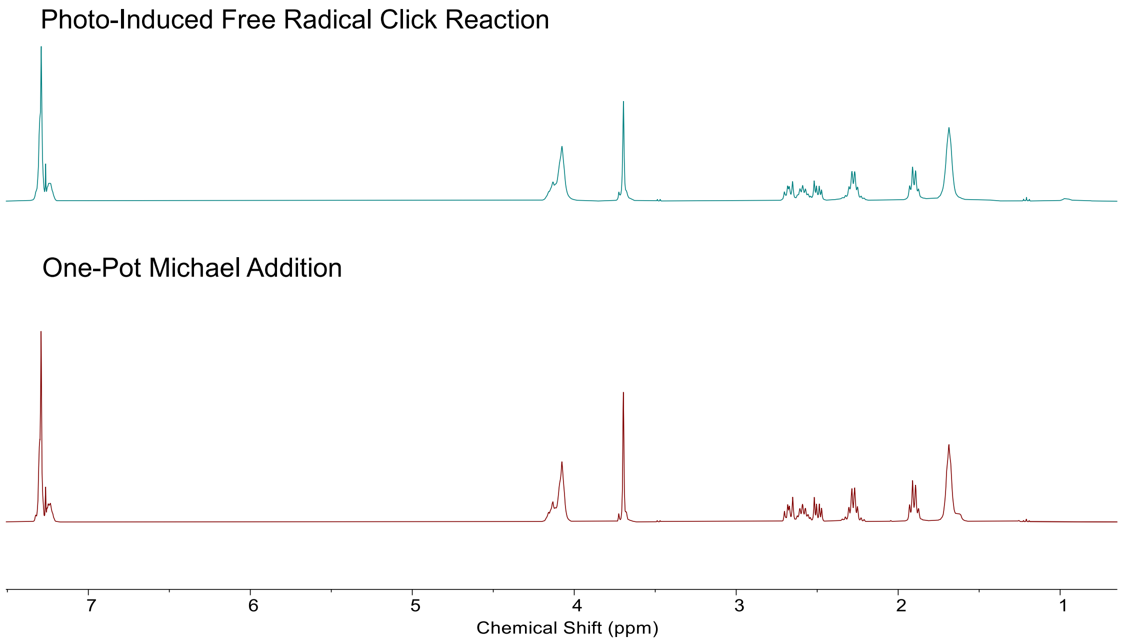

Figure S63. Overlaid ^{1}H NMR spectra (400 MHz, $CDCl_3$) of PBDA–BnSH acquired from one-pot thiol-Michael addition click reaction and photo-induced free radical click reaction.

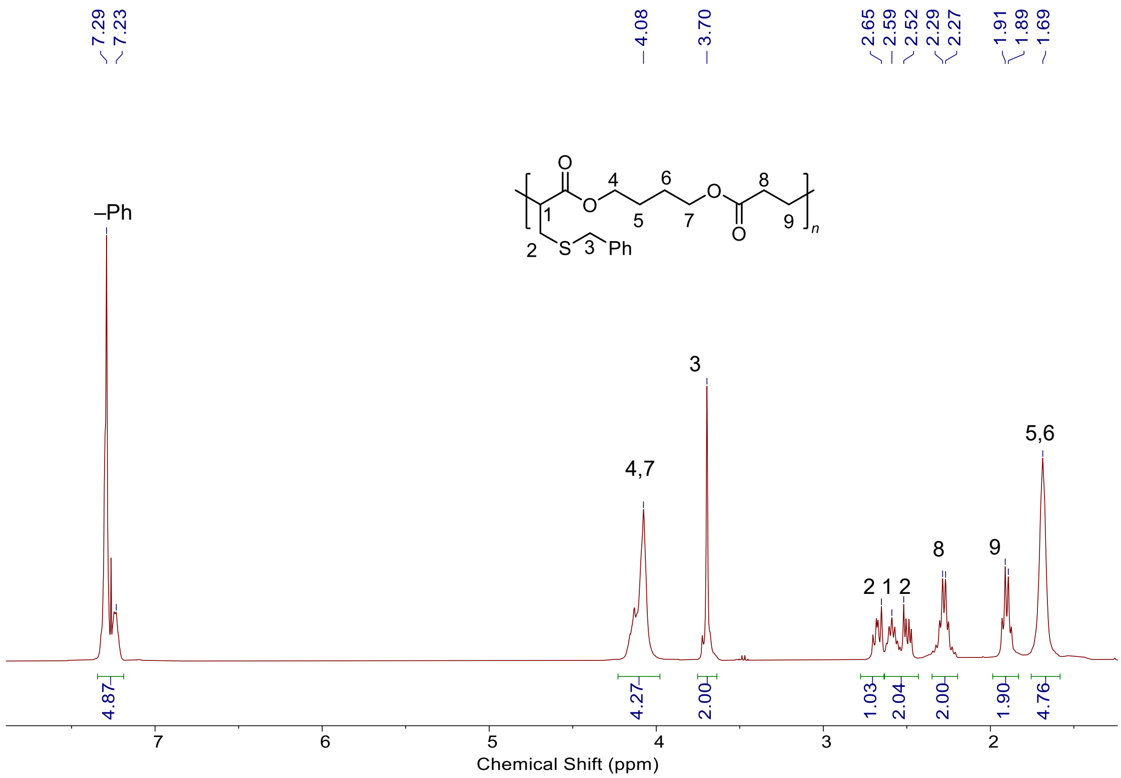

Figure S64. ^{1}H NMR (400 MHz, $CDCl_3$) spectrum of PBDA–BnSH.

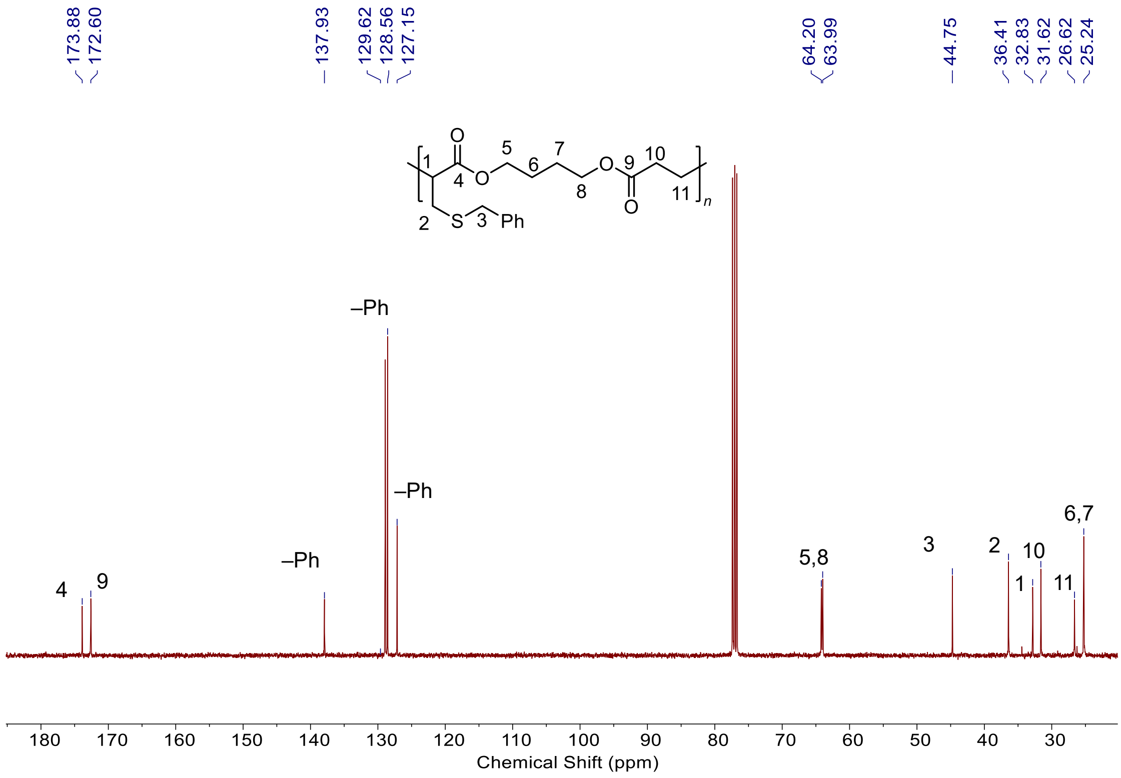

Figure S65. ^{13}C NMR (101 MHz, $CDCl_3$) spectrum of PBDA–BnSH.

One-Pot Synthesis of PDoDA–ME

The polymerization condition was the same as it was stated for Entry 6, Table 2. When the conversion of DoDA reached 78% in 12 hours, the polymerization system was diluted with toluene (500 μL), and then 2-mercaptoethanol (350 μL, 10 equivalent to DoDA monomer) was added to the reaction mixture. After 4 more hours, the reaction was quenched by adding $CHCl_3$ (2 mL), and then the diluted reaction mixture was precipitated into an excess of diethyl ether and centrifugated. The precipitate was then dissolved in $CHCl_3$ (2 mL) again, precipitated into an excess of ether and centrifugated for two more cycles. The precipitate was finally dried under vacuum.

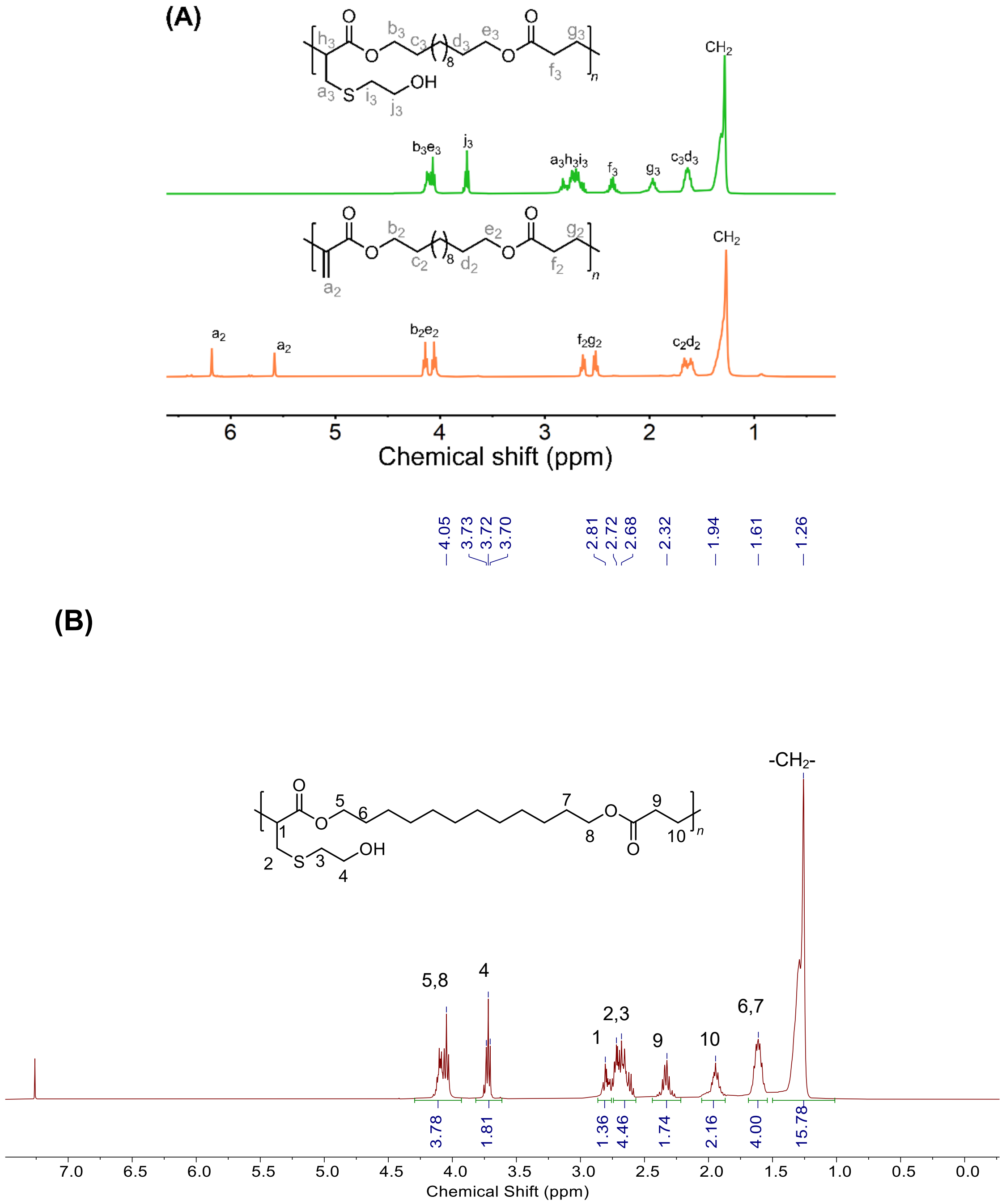

Figure S66. (A) The overlaid ¹H NMR (400 MHz, $CDCl_3$) spectra of PDoDA and PDoDA–ME. **(B)** ¹H NMR (400 MHz, $CDCl_3$) spectrum of PDoDA–ME.

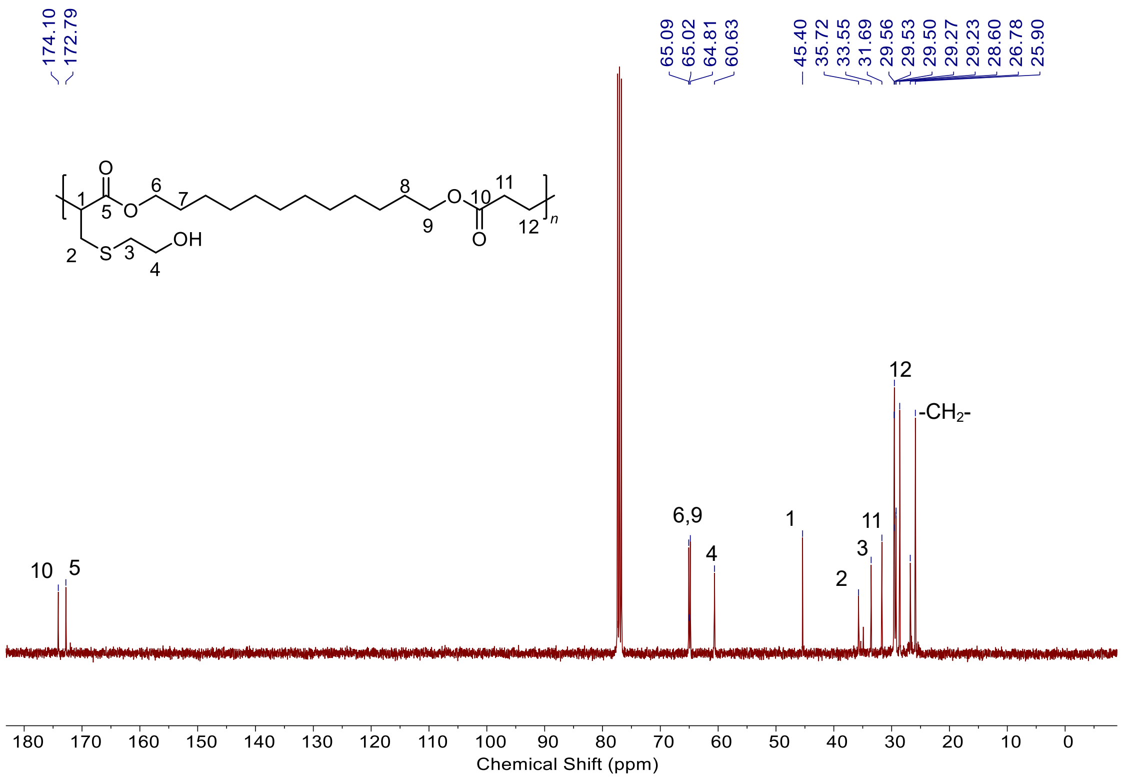

Figure S67. ^{13}C NMR (101 MHz, $CDCl_3$) spectrum of PDoDA–ME.

Synthesis of PDoDA1 and PDoDA2

In an argon-filled glovebox, DoDA (155 mg, 0.5 mmol) was dissolved in toluene (190 μL) in a 5 mL vial containing a stir bar, then PCy_3 (50 μL, 0.5 mol·L^{-1} in toluene) was added to the above solution to set the initial concentration of monomer to 1.5 mol L^{-1} and [monomer]:[catalyst] ratio to 20:1. For PDoDA1, the reaction time was 6 h. For PDoDA2, the reaction time was 9 h. The vial was sealed and left to stir in the glovebox under room temperature. The polymerization was quenched by adding $CHCl_3$, and then the diluted reaction mixture was precipitated into an excess of n-pentane and centrifugated. The precipitate was then dissolved in $CHCl_3$ (2 mL) again, precipitated into an excess of n-pentane and centrifugated for three more cycles. The precipitate was finally dried under vacuum.

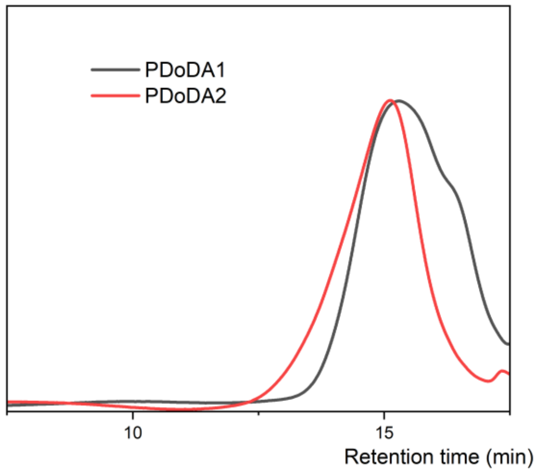

Figure S68. SEC traces (THF as the eluent) of PDoDA1 and PDoDA2.

Preparation of Bottlebrush Copolymers PDoDA-*g*-PLLA$_m$

Taking the synthesis of PDoDA-*g*-PLLA$_{188}$ as a representative example. To a flame-dried Schlenk tube equipped with a magnetic stir bar, L-LA (720 mg, 5 mmol), a stock solution of PDoDA–ME (250 µL, THF as solvent, [–OH] = 0.1 mol/L), a stock solution of stannous octoate (10 µL, toluene as solvent, [Sn(Oct)$_2$] = 0.5 mol/L), and 2 mL toluene was added. Then the tube was sealed with a Teflon lined cap, taken out from the glovebox and placed in a 120 °C heating block. The reaction mixture was left to stir for 12 hours. Then the tube was taken out of the heating block, cooled to room temperature to quench the polymerization. The residue was dissolved in DCM (10 mL) and then precipitated into an excess of cold methanol. The obtained precipitate was washed with methanol several times and then dried under vacuum at room temperature.

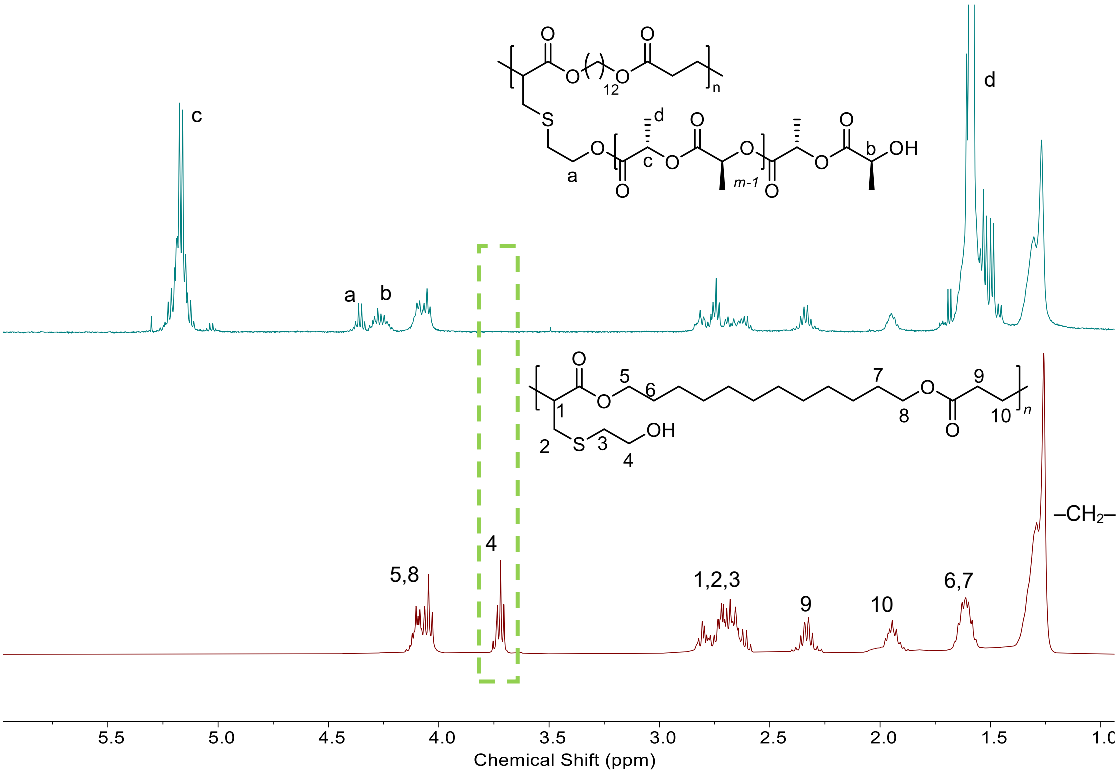

Figure S69. Overlaid ^{1}H NMR spectra (400 MHz, CDCl$_3$) of PDoDA–ME and PDoDA-*g*-PLLA$_m$. The disappearance of the peak at 3.72 ppm suggested the grafting density is close to 100%.

Preparation of PLLA Homopolymer

To a flame-dried Schlenk tube equipped with a magnetic stir bar was added L-LA (720 mg, 5 mmol), a stock solution of stannous octoate (10 µL, toluene as solvent, [Sn(Oct)$_2$] = 0.5 mol/L) and 2 mL toluene was added. Then the tube was sealed with a Teflon lined cap, taken out from the glovebox and placed in a 110 °C heating block. The reaction mixture was left to stir for 15 hours. Then the tube was taken out of the heating block, cooled to room temperature to quench the polymerization. The residue was diluted with DCM (10 mL) and then precipitated into an excess of cold methanol. The obtained precipitate was washed with methanol several times and then dried under vacuum at room temperature.

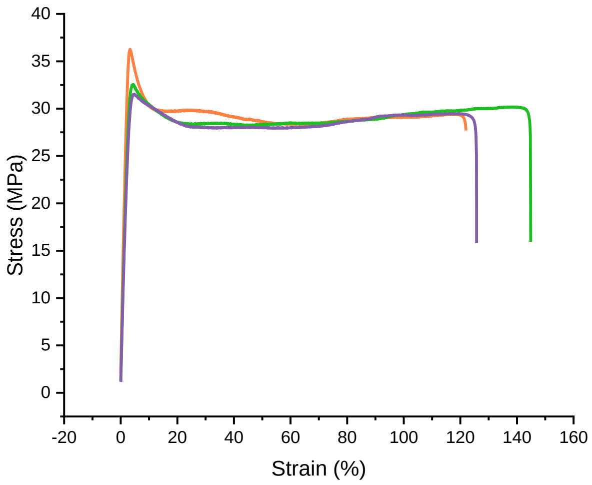

Figure S70. Tensile tests for PDoDA-*g*-PLLA$_{188}$.

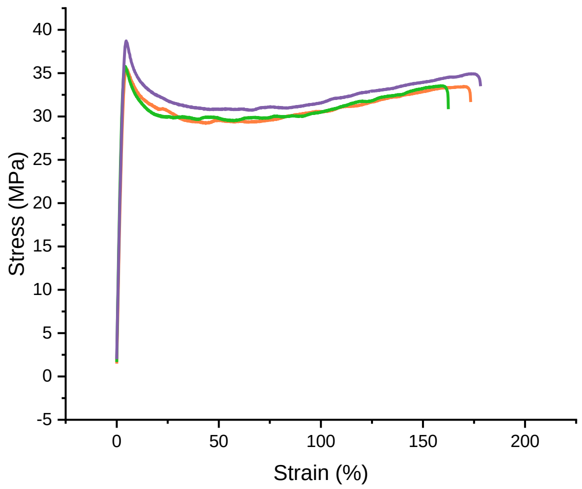

Figure S71. Tensile tests for PDoDA-*g*-PLLA$_{465}$.

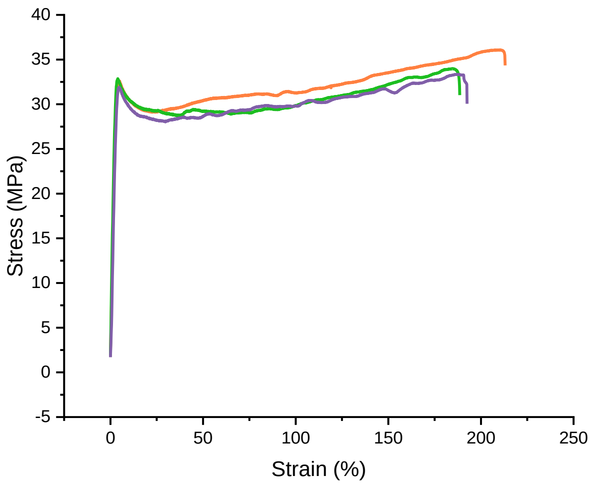

Figure S72. Tensile tests for PBDA-*graft*-PLLA$_{924}$.

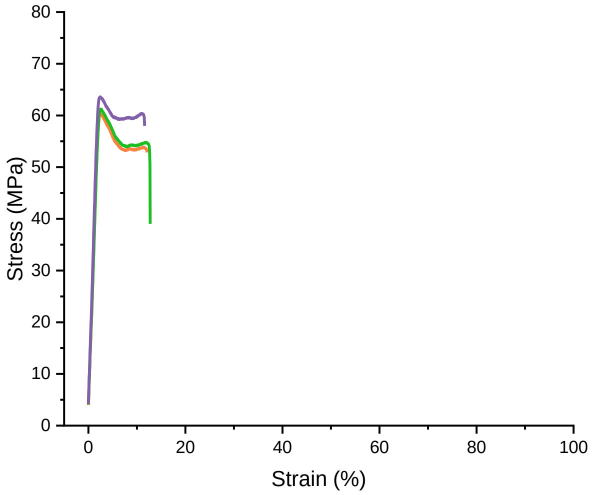

Figure S73. Tensile tests for PLLA homopolymer.

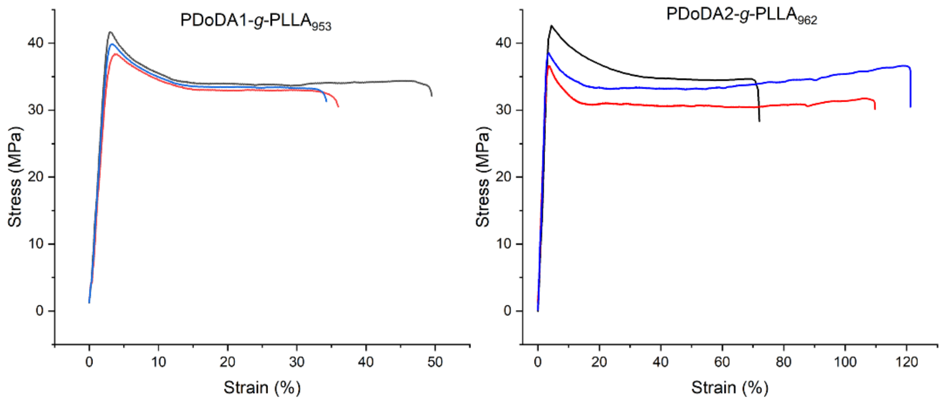

Figure S74. Tensile tests for PDoDA1-*g*-PLLA$_{953}$ and PDoDA2-*g*-PLLA$_{962}$.

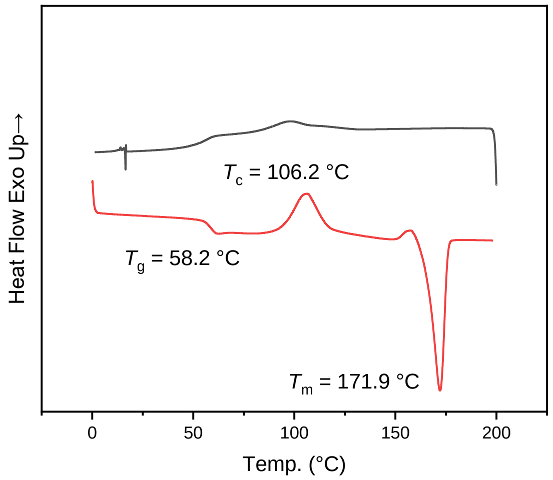

Figure S75. DSC curves of PDoDA-*g*-PLLA$_{188}$.

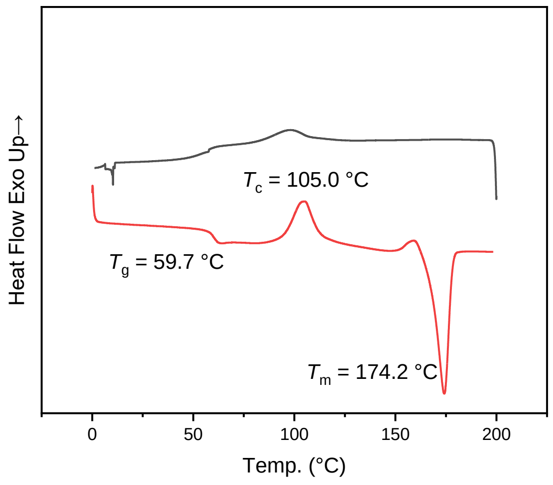

Figure S76. DSC curves of PDoDA-*g*-PLLA$_{465}$.

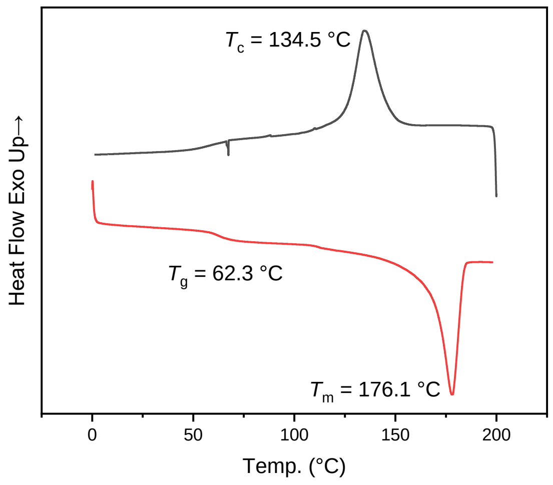

Figure S77. DSC curves of PDoDA-*g*-PLLA$_{924}$.

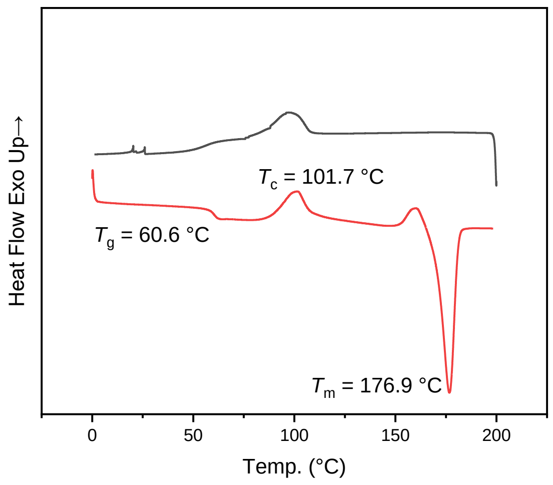

Figure S78. DSC curves of homopolymer PLLA.

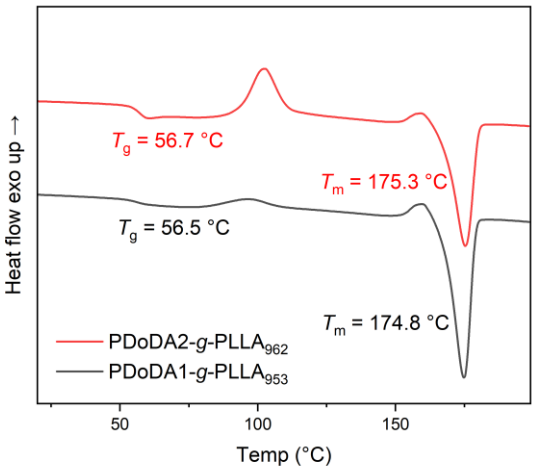

Figure S79. DSC curves of PDoDA1-*g*-PLLA$_{953}$ and PDoDA2-*g*-PLLA$_{962}$.

Table S2. The comparison of mechanical and thermal properties for PLLA and graft polymers PDoDA-*g*-PLLA$_m$ with different main-chain lengths.

Polymer	E (GPa)	ε_b (%)	$U_T{}^c$ (MPa)	T_m (°C)	ΔH_f (J/g)
PDoDA1-*g*-PLLA$_{953}$	1.48±0.10	37.5±10.4	13.3±3.1	174.8	36.13
PDoDA2-*g*-PLLA$_{962}$	1.29±0.07	101±25.0	33.4±8.0	175.3	34.90
PDoDA-*g*-PLLA$_{924}$	1.22±0.07	198±11	61.1±5.0	176.1	30.24
PLLA	2.97±0.08	12.1±0.5	6.4 ± 0.2	176.9	36.76

References

1. Sun M, Hong C-Y, Pan C-Y. A Unique Aliphatic Tertiary Amine Chromophore: Fluorescence, Polymer Structure, and Application in Cell Imaging. *J Am Chem Soc*. 2012; **134**: 20581–4

2. Parrott MC, Luft JC, Byrne JD *et al.* Tunable Bifunctional Silyl Ether Cross-Linkers for the Design of Acid-Sensitive Biomaterials. *J Am Chem Soc*. 2010; **132**: 17928–32

3. Petersen SR, Prydderch H, Worch JC *et al.* Ultra-Tough Elastomers from Stereochemistry-Directed Hydrogen Bonding in Isosorbide-Based Polymers. *Angew Chem, Int Ed*. 2022; **61**: e202115904

4. Flory PJ. Statistical Mechanics of Swelling of Network Structures. *J Chem Phys*. 1950; **18**: 108–11

5. Phillip WA, Hillmyer MA, Cussler EL. Cylinder Orientation Mechanism in Block Copolymer Thin Films Upon Solvent Evaporation. *Macromolecules*. 2010; **43**: 7763–70
